# Visible-Light Photoredox-Catalyzed Amidation of Benzylic Alcohols

**DOI:** 10.1021/acs.joc.0c01320

**Published:** 2020-07-14

**Authors:** Silvia Gaspa, Andrea Farina, Mariella Tilocca, Andrea Porcheddu, Luisa Pisano, Massimo Carraro, Ugo Azzena, Lidia De Luca

**Affiliations:** † Dipartimento di Chimica e Farmacia, Università degli Studi di Sassari, Via Vienna 2, 07100 Sassari, Italy; ‡ Dipartimento di Scienze Chimiche e Geologiche, Università degli Studi di Cagliari, Cittadella Universitaria, 09042 Monserrato, Italy

## Abstract

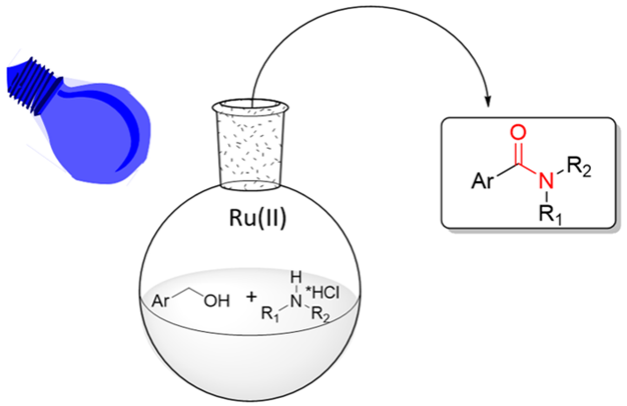

A new photocatalyzed route to amides from alcohols and amines mediated by visible light is presented. The reaction is carried out in ethyl acetate as a solvent. Ethyl acetate can be defined a green and bio-based solvent. The starting materials such as the energy source are easily available, stable, and inexpensive. The reaction has shown to be general and high yielding.

## Introduction

The amide bond is contained in many both synthetic compounds, such as drugs, polymers, detergents, and natural compounds, such as peptides and proteins.^1^ The amide bond formation reactions are among the most studied in organic chemistry and the most frequent in biochemistry due to the widespread occurrence of these compounds. Classically, amide bonds are synthesized by acylation of amines with carboxylic acid derivatives (activated esters, acyl chlorides, carboxylic anhydrides): indeed, this is the most common synthetic pathway employed in the industrial synthesis of pharmaceuticals. This approach presents many disadvantages such as two additional steps with consequent increasing production of byproducts and reduction in the yield of final products, use of highly hazardous reagents, and production of a stoichiometric amount of waste products.^2^ One of the most challenging research topics in organic synthesis is the development of new methodologies induced by visible light.^3^ Photosynthesis can be defined as the transformation of sunlight into chemical energy effectively used to promote chemical reaction, allowing the development of sustainable and efficient procedures. Visible light can be considered as a clean reagent: it activates the substrates leaving no residues in the reaction mixture, with considerable simplification of the workup and purification processes. For these reasons, visible-light-mediated organic synthesis has achieved strong priority because of the development of sustainable chemistry routes.^4^

**Scheme 1:**
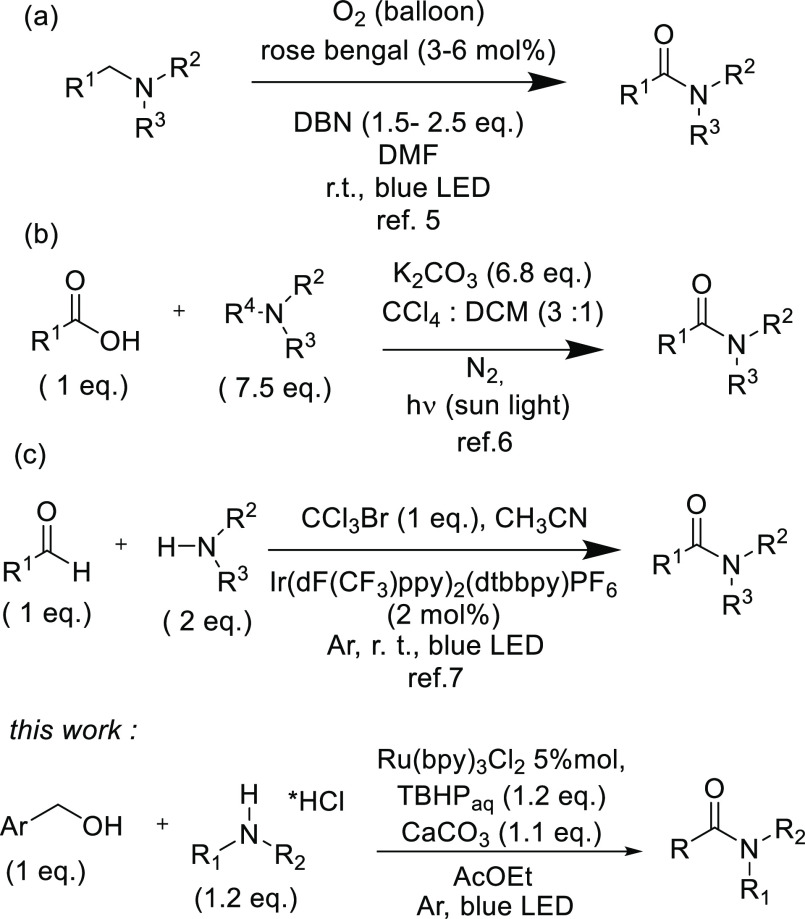
Synthesis of Amides Mediated by Visible Light

In this context, the synthesis of amides mediated by visible light has recently been studied and few methods have been mentioned in the literature. Recently, Das and co-workers have reported an α-oxygenation of tertiary amines to tertiary amides (Scheme 1; path a).^5^ The methodology makes use of O_2_ as an oxidant, 1,5-diazabicyclo[4.3.0]non-5-ene (DBN) as a base, *N*,*N*-dimethylformamide (DMF) as a solvent, and Rose Bengal as a photocatalyst. Even if the reaction scope is intimately linked to the nature and availability of tertiary amines, the work is pioneering for the visible-light-mediated amide synthesis. In this context, an interesting dealkylative condensation of tertiary amines with carboxylic acids to amides has been proposed by Szpilman (Scheme 1; path b).^6^ The reaction proceeds through a charge-transfer complex between the amine and carbon tetrachloride, and the large excesses of reagents and solvents should be easily recyclable in an industrial setting. Aldehydes have also been employed as starting materials for the visible-light-mediated synthesis of amides (Scheme 1; path c).^7^ Pandey has reported a direct amidation of aldehydes by a cross-dehydrogenative coupling catalyzed by an iridium-based catalyst in the presence of 1 equiv of CCl_3_Br used as a stoichiometric oxidant. Aldehydes are normally obtained by selective oxidation of alcohols, which are easily available and stable compounds, and are contained in many naturally occurring organic molecules. For these reasons, the direct conversion of alcohols to amides is a highlight of green and sustainable chemistry.^8^ The only paper reporting a visible-light-mediated conversion of alcohols to amides was presented by Cai.^9^ It is an oxidative cross-coupling reaction of benzyl alcohols with *N*,*N*-dimethyl acetamide, used both as a solvent and as a reagent, to obtain exclusively *N*,*N*-dimethyl cinnamides. In this work, we report a new photocatalyzed route to amides from alcohols and amines mediated by visible light.

## Results and Discussion

In relation to our interest in the synthesis of amides,^10^ we have checked the possibility to explore a new visible-light-mediated method, which would give a broad access to amides from alcohols. We started our investigation using benzyl alcohol **1a** and *N*,*N*-dimethylamine hydrochloride **2a** as the model substrates and blue LEDS as the light source.

Benzyl alcohol (Table 1, **1a**, 1 mmol), *N*,*N*-dimethylamine hydrochloride (Table 1, **2a**, 1.2 mmol), aqueous 70% TBHP (Table 1, 2.2 mmol), CaCO_3_ (Table 1, 1.1 mmol), and [Ru(bpy)_3_Cl_2_] (Table 1, 5 mol %) in 0.5 mL of acetonitrile were irradiated for 72 h with blue LED_S_, but no product was formed (Table 1, entry 1). The same reaction was carried out in cyclopentyl methyl ether (CPME) as a solvent, and the product **3aa** was formed in 49% yield (Table 1, entry 2). To improve the yield, AcOEt was employed as a solvent and the product **3aa** was formed in 83% yield (Table 1, entry 3). If the reaction was carried out by TBHP 5.5 M in decane as an oxidant, the yield decreased to 16% (Table 1, entry 4). A different oxidizing reagent as H_2_O_2_ was tested, in respectively CPME (Table 1, entry 5), acetonitrile (Table 1, entry 6), and AcOEt (Table 1, entry 7), but no product was detected in all cases. Subsequently, O_2_ was tested in CPME (Table 1, entry 8) and AcOEt (Table 1, entry 9), but no product was formed. Using Eosin Y as a catalyst, instead of [Ru(bpy)_3_Cl_2_], both under blue LED (Table 1, entry 10) and green LED (Table 1, entry 11) irradiation, the product was obtained in low yields of 36 and 35%, respectively. The reaction performed with Rose Bengal as a catalyst and, respectively, TBHP_aq_ and O_2_ as oxidants, the product was isolated only in traces (Table 1, entries 12 and 13). Finally, the reaction was carried out in the dark for 72 h (Table 1, entry 14) and 96 h (Table 1, entry 15), and in both cases, the product was obtained in a low yield (33%).

**Table 1:**
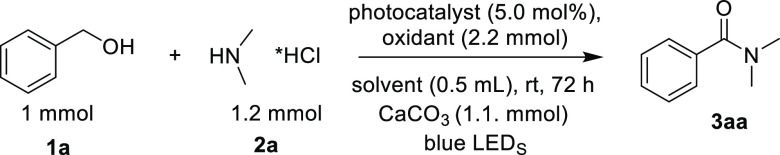
Screening of Reaction Conditions

entry^a^	photocatalyst	oxidant	solvent	yield (%)^b^
1	[Ru(bpy)_3_Cl_2_]	TBHP_aq_	acetonitrile	
2	[Ru(bpy)_3_Cl_2_]	TBHP_aq_	CPME	49
3	[Ru(bpy)_3_Cl_2_]	TBHP_aq_	AcOEt	83
4	[Ru(bpy)_3_Cl_2_]	TBHP_dec_	AcOEt	16
5	[Ru(bpy)_3_Cl_2_]	H_2_O_2_	CPME	
6	[Ru(bpy)_3_Cl_2_]	H_2_O_2_	acetonitrile	
7	[Ru(bpy)_3_Cl_2_]	H_2_O_2_	AcOEt	
8	[Ru(bpy)_3_Cl_2_]	O_2_	CPME	
9	[Ru(bpy)_3_Cl_2_]	O_2_	AcOEt	
10	Eosin Y	TBHP_aq_	AcOEt	36
11^c^	Eosin Y	TBHP_aq_	AcOEt	35
12	Rose Bengal	TBHP_aq_	AcOEt	7
13	Rose Bengal	O_2_	AcOEt	
14	[Ru(bpy)_3_Cl_2_]	TBHP_aq_	AcOEt	33
15^d^	[Ru(bpy)_3_Cl_2_]	TBHP_aq_	AcOEt	33

a General reaction conditions: **1a** (1 mmol), **2a** (1.2 mmol), photocatalyst (5.0 mol %), oxidant (2.2 mmol), and CaCO_3_ (1.1 mmol) in solvent (0.5 mL) at rt in argon for 72 h with 9 W blue LED_S_.

b Yield of the isolated product.

c The reaction was performed with 9 W green LED_S_.

d The reaction was carried out for 96 h.

The use of AcOEt as a solvent is profitable. AcOEt highly is a recommended solvent by Sanofi’s solvent selection guide^11^ because it is biodegradable and non bioaccumulable and has an ecotox >100 mg/L, with ICH limits (ppm) of 5000. It is included in the greener solvent GSK’s list^12^ too, and its use is high recommended. Moreover, ethyl acetate is commercially available, easily accessible, and inexpensive.

**Scheme 2:**
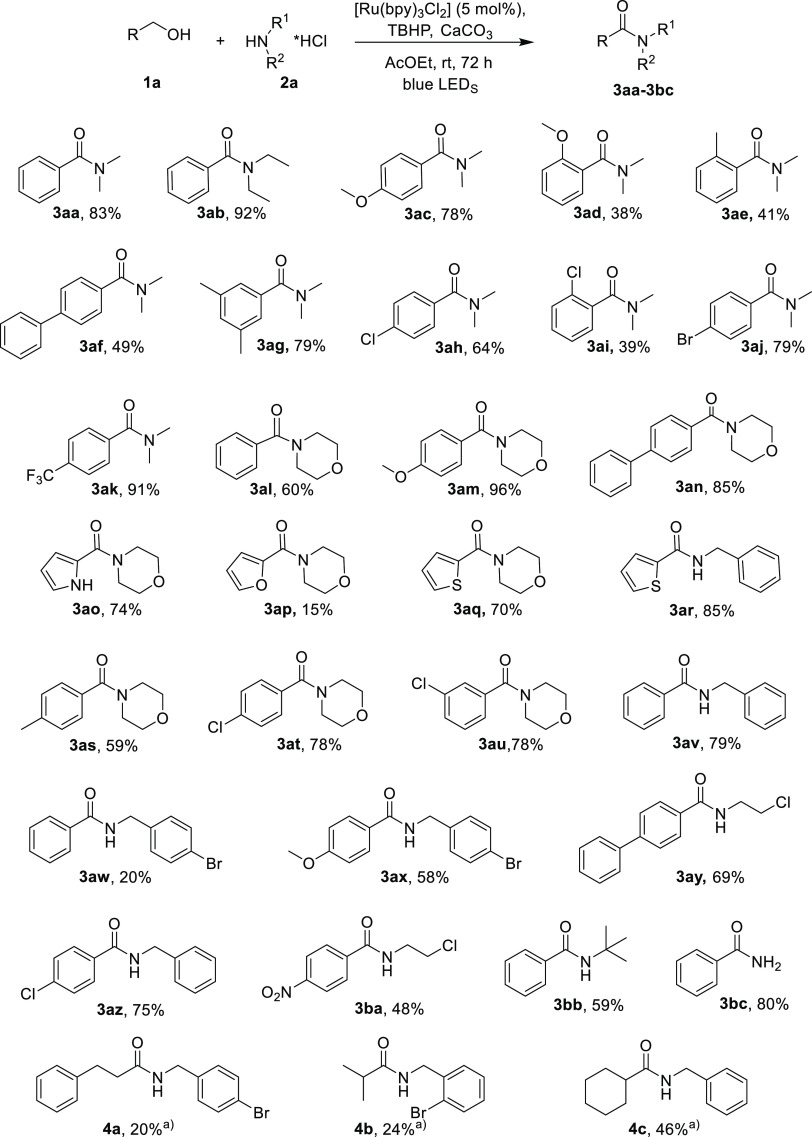
Investigation of the Alcohol Scope of the Reaction Reaction condition: aliphatic aldehyde (1 mmol), amine hydrochloride salt (1.2 mmol), Ru(bpy)_3_Cl_2_*6H_2_O (0.05 mmol, 5.0 mol %), CaCO_3_ (1.1 mmol), and *tert*-butyl hydroperoxide (70 wt % in H_2_O, 1.1 mmol) in ethyl acetate (0.5 mL), under argon atmosphere and blue LED for 48 h.

With the optimized conditions in hand (Table 1, entry 3), the reaction scope was investigated. An array of substituted benzyl alcohols and amine hydrochloride was tested. In general, the corresponding amides were obtained in good yields (Scheme 2; **3aa**–**bc**). Different functional groups on aromatic rings both electron-donating and electron-withdrawing were tested. Neither the electronic properties nor the steric effects of substituents on the aromatic ring of benzylic alcohols were found to have any effect on the reaction and products yield.

Strong electron-donating groups as OMe (Scheme 2; **3ac**, **3ad**, **3am**, and **3ax**) showed good results. Benzyl alcohols with moderate electron-donating substituents as phenyl (Scheme 2; **3af, 3an**, and **3ay**) and methyl (Scheme 2; **3ae, 3ag**, and **3as**) were tested with satisfactory results. Benzyl alcohols with halide substituents, such as chlorine and bromide, in different positions were subjected to this procedure giving the corresponding amides, which could be further transformed by traditional cross-coupling reactions (Scheme 2; **3ah**, **3ai**, **3aj**, **3at**, **3au**, and **3az**). Strong electron-withdrawing groups such as NO_2_ (Scheme 2; **3ba**) and CF_3_ (Scheme 2; **3ak**) were well tolerated providing the desired amides in good results. The reactions with sterically hindered ortho-substituted benzylic alcohols were performed, and the corresponding amides (Scheme 2; **3ad**, **3ae**, and **3ai**) were obtained in low yields as expected because of steric hindrance. To prove the synthetic utility of the procedure, (1*H*-pyrrol-2-yl) methanol, furan-2-ylmethanol, and thiophen-2-ylmethanol were subjected to optimized reactions conditions, giving the desired heteroaryl amides (Scheme 2; **3ao**, **3ap**, **3aq**, and **3ar**).

To investigate the scope of the method, the reaction was investigated with an array of primary and secondary hydrochloride ammines showing excellent tolerance. Secondary aliphatic acyclic amines are *N*,*N*-dimethylamine (Scheme 2; **3aa**, **3ac**, **3ad**, **3ae, 3af, 3ag, 3ah, 3ai, 3aj**, and **3ak**) and *N*,*N*-diethyl amine (Scheme 2; **3ab**); secondary aliphatic cyclic amines are morpholine (Scheme 2; **3al**, **3am**, **3an**, **3ao**, **3ap**, **3aq**, **3as**, **3at**, and **3au**); and primary aliphatic amines are *N*-benzylamine (Scheme 2; **3ar**, **3av**, and **3az**), *N*-4-bromobenzylamine (Scheme 2; **3aw** and **3ax**), 2-chloroethylamine (Scheme 2; **3ay** and **3ba**), and *tert*-butylamine (Scheme 2; **3bb**). The reaction carried out with hydrochloride ammonium salts was performed, and the corresponding primary amide, benzamide (Scheme 2; **3bc**), was obtained in a very good yield. The reaction was investigated with aniline, but no product was detected. The procedure was applied to aliphatic alcohols, but at the end of the entire procedure, the corresponding alcohols were recovered unreacted. Finally, aliphatic aldehydes were tested, giving the desired amides in acceptable yields (Scheme 2; **4a**, **4b**, and **4c**).^13^

**Scheme 3:**
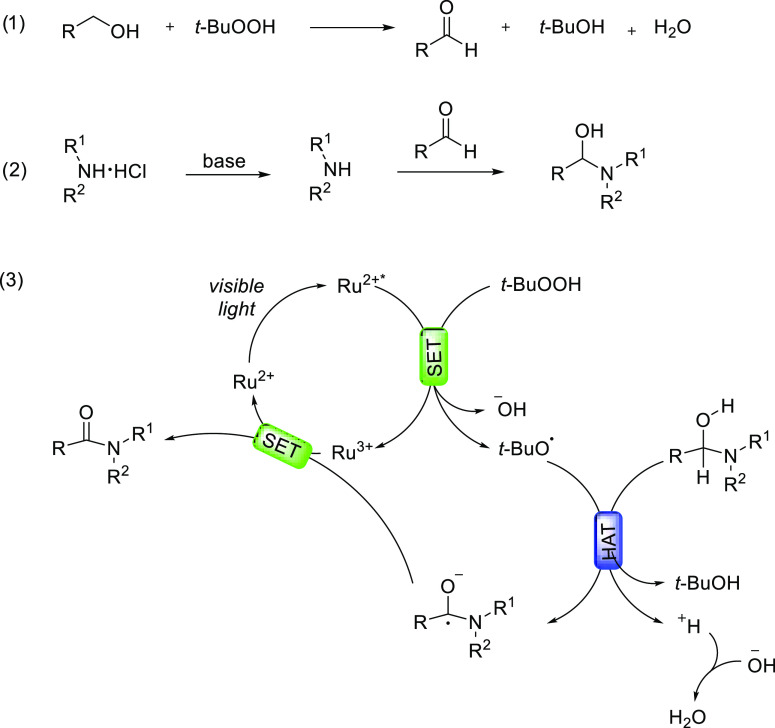
Proposed Reaction Mechanism

A plausible reaction mechanism is reported in Scheme 3. On the basis of previously published paper,^8c,14^ alcohol is oxidized by TBHP to aldehyde. The aldehyde reacts with the amine to form hemiaminal.^15^

The reaction proceeds by photoexcitation of [Ru(bpy)_3_Cl_2_], which is known to undergo single electron transfer (SET) in the presence of *tert*-butyl hydroperoxide, thus generating two species: *tert*-butoxy radical *t*-BuO^•^ and hydroxyl anion HO^–^. Subsequently, *t*-BuO^•^ may abstract by hydrogen atom transfer (HAT) a hydrogen from the hemiaminal. The C-radical will be deprotonated to give the ketyl radical anion, which then reduces the Ru(III) complex by an SET, which completes the photoredox catalytic cycle of Ru catalyst and hence its regeneration.

Quantum yield measurements were performed to provide insight into the effective reaction mechanism,^16^ and the results indicate a light-initiated chain propagation mechanism.

Briefly, standard chemical actinometry using potassium ferrioxalate allowed us to determine the photon flux of a blue LED at 455 nm. After 180 s of irradiation of reaction mixture at 455 nm, a 5% conversion to amide **3aa** is observed. This yield corresponds to 51 equiv of product formed per absorbed photon (ϕ = 51), thus indicating that this reaction proceeds through a chain propagation mechanism.

## Conclusions

In conclusion, the first example of photocatalyzed amides synthesis from alcohols and amines mediated by visible light was reported. The reaction is carried out in ethyl acetate as a green, eco-sustainable, and bio-based solvent. The starting materials are stable, easily accessible, and inexpensive. The reaction conditions are mild, the stoichiometric ratio of the reactants is optimal, and the use of visible light as a source of energy is very appealing from an ecological point of view. The methodology has shown a big versatility and applicability, while various functional groups, on both alcohols and amines, are well tolerated, providing a new approach to visible-light-mediated amide synthesis.

## Experimental Section

### General Information

All reagents and solvents were used as obtained from a commercial source. All solvents were dried by usual methods and distilled under argon. Column chromatography was generally performed on silica gel (pore size, 60 Å; 32–63 nm particle size), and reactions were monitored by thin-layer chromatography (TLC) analysis performed with Merck Kieselgel 60 F254 plates and visualized using UV light at 254 nm, KMnO_4_, and 2,4-DNP staining. For irradiation with blue light, an OSRAM Oslon SSL 80 LDCQ7P-1U3U (blue, λ_max_ = 455 nm, *I*_max_ = 1000 mA, 1.12 W) was used. ^1^H NMR and ^13^C NMR spectra were measured on a Bruker Avance III 400 spectrometer (400 or 100 MHz, respectively) using CDCl_3_ solutions and TMS as an internal standard. Chemical shifts are reported in parts per million (ppm, δ) relative to internal tetramethylsilane standard (TMS, δ 0.00). The peak patterns are indicated as follows: s, singlet; d, doublet; t, triplet; m, multiplet; q, quartet; dd, doublet of doublets; brs, broad. The coupling constants, *J*, are reported in hertz (Hz). High-resolution mass spectra HRMS (HESI-FT-ORBITRAP) were recorded on a Q-Exactive Thermo Scientific mass spectrometer. UV–vis spectra were obtained on a T80+ UV/vis spectrometer (PG Instruments Ltd). Melting points were determined in open capillary tubes and are uncorrected.

### General Procedure to Compounds **3aa**–**3bc**

To a mixture of amine hydrochloride salt (1.2 mmol), Ru(bpy)_3_Cl_2_*6H_2_O (37.4 mg, 0.05 mmol, 5.0 mol %), and CaCO_3_ (110 mg, 1.1 mmol) in ethyl acetate (0.5 mL) were added alcohol (1 mmol) and *tert*-butyl hydroperoxide (70 wt % in H_2_O, 0.31 mL, 2.2 mmol) under an argon atmosphere at room temperature. The reaction mixture was irradiated under blue LED and stirred at room temperature for 72 h (monitored by TLC until disappearance of the alcohol). Then, the reaction mixture was quenched with water and extracted with AcOEt. The combined organic layers were washed three times with a solution of 5% citric acid and then three times with a solution of 5% NaHCO_3_; the organic phase was dried over anhydrous Na_2_SO_4_, and the solvent was evaporated under reduced pressure. The crude products were purified by flash chromatography on silica gel.

### Compound Characterizations

#### *N*,*N*-Dimethylbenzamide (**3aa**)^17^

Yellow oil (124 mg, 83% yield); *R*_*f*_ = 0.33 (hexane/ethyl acetate 2/3). ^1^H NMR (400 MHz, CDCl_3_) δ: 7.40–7.39 (m, 5H), 3.11 (s, 3H), 2.97 (s, 3H). ^13^C{^1^H} NMR (100 MHz, CDCl_3_) δ: 171.7, 136.3, 129.5, 128.3, 127.0, 39.6, 35.3.

#### *N*,*N*-Diethylbenzamide (**3ab**)^18^

Colorless oil (163 mg, 92% yield); *R*_*f*_ = 0.37 (hexane/ethyl acetate 2/3). ^1^H NMR (400 MHz, CDCl_3_) δ: 7.35–7.31 (m, 5H), 3.49 (brs, 2H), 3.22 (brs, 2H), 1.22–1.08 (m, 6H). ^13^C{^1^H} NMR (100 MHz, CDCl_3_) δ: 171.1, 137.1, 128.9, 128.2, 126.1, 43.1, 39.1, 14.0, 12.8.

#### 4-Methoxy-*N*,*N*-dimethylbenzamide (**3ac**)^19^

Yellow oil (140 mg, 78% yield); *R*_*f*_ = 0.30 (hexane/ethyl acetate 2/3). ^1^H NMR (400 MHz, CDCl_3_) δ: 7.39 (d, *J* = 8.7 Hz, 2H), 6.89 (d, *J* = 8.7 Hz, 2H), 3.82 (s, 3H), 3.04 (s, 6H). ^13^C{^1^H} NMR (100 MHz, CDCl_3_) δ: 171.5, 160.6, 129.1, 128.3, 113.5, 55.3, 39.8, 35.6.

#### 2-Methoxy-*N*,*N*-dimethylbenzamide (3ad)^20^

Yellow oil (68 mg, 38% yield); *R*_*f*_ = 0.27 (hexane/ethyl acetate 2/3). ^1^H NMR (400 MHz, CDCl_3_) δ: 7.31 (t, *J* = 8.0 Hz, 1H), 7.20 (d, *J* = 7.4 Hz, 1H), 6.95 (t, *J* = 7.5 Hz, 1H), 6.88 (d, *J* = 8.3 Hz, 1H), 3.81 (s, 3H), 3.09 (s, 3H), 2.82 (s, 3H). ^13^C NMR (100 MHz, CDCl_3_) δ: 169.3, 155.2, 130.2, 127.8, 126.2, 120.8, 110.8, 55.4, 38.1, 34.6.

#### *N*,*N*,2-Trimethylbenzamide (**3ae**)^21^

Yellow oil (67 mg, 41% yield); *R*_*f*_ = 0.37 (hexane/ethyl acetate 2/3). ^1^H NMR (400 MHz, CDCl_3_) δ: 7.26–7.22 (m, 1H), 7.21–7.14 (m, 3H), 3.12 (s, 3H), 2.82 (s, 3H), 2.28 (s, 3H). ^13^C{^1^H} NMR (100 MHz, CDCl_3_) δ: 171.5, 136.7, 133.9, 130.3, 128.7, 125.9, 125.8, 38.3, 34.5, 18.9.

#### *N*,*N*-Dimethyl-[1,1’-biphenyl]-4-carboxamide (**3af**)^22^

White solid (110 mg, 49% yield); mp 104–105 °C,^22^*R*_*f*_ = 0.26 (hexane/ethyl acetate 2/3). ^1^H NMR (400 MHz, CDCl_3_) δ: 7.63–7.59 (m, 4H), 7.53–7.42 (m, 4H), 7.39–7.35 (m, 1H), 3.09 (brs, 6H). ^13^C{^1^H} NMR (100 MHz, CDCl_3_) δ: 171.5, 142.4, 140.3, 135.0, 128.9, 127.7, 127.7, 127.1, 127.0, 38.7, 35.3.

#### *N*,*N*,3,5-Tetramethylbenzamide (**3ag**)^23^

Yellow oil (140 mg, 79% yield); *R*_*f*_ = 0.41 (hexane/ethyl acetate 2/3). ^1^H NMR (400 MHz, CDCl_3_) δ: 7.00 (s, 1H), 6.98 (s, 2H), 3.07 (s, 3H), 2.96 (s, 3H), 2.31 (s, 6H). ^13^C NMR (100 MHz, CDCl_3_) δ: 172.0, 137.9, 136.2, 130.9, 124.5, 39.5, 35.2, 21.2.

#### 4-Chloro-*N*,*N*-dimethylbenzamide (**3ah**)^19^

Yellow oil (117 mg, 64% yield); *R*_*f*_ = 0.44 (hexane/ethyl acetate 2/3). ^1^H NMR (400 MHz, CDCl_3_) δ: 7.39–7.35 (m, 4H), 3.10 (s, 3H), 2.97 (s, 3H). ^13^C{^1^H} NMR (100 MHz, CDCl_3_) δ: 170.6, 135.6, 134.6, 128.6, 128.6, 39.6, 35.4.

#### 2-Chloro-*N*,*N*-dimethylbenzamide (**3ai**)^24^

Yellow oil (72 mg, 39% yield); *R*_*f*_ = 0.4 (hexane/ethyl acetate 2/3). ^1^H NMR (400 MHz, CDCl_3_) δ: 7.41–7.37 (m, 1H), 7.33–7.28 (m, 3H), 3.14 (s, 3H), 2.86 (s, 3H). ^13^C NMR (100 MHz, CDCl_3_) δ: 168.4, 136.4, 130.3, 130.0, 129.5, 127.8, 127.1, 38.0, 34.7.

#### 4-Bromo-*N*,*N*-dimethylbenzamide (**3aj**)^25^

White solid (180 mg, 79% yield); mp 76–77 °C, *R*_*f*_ = 0.23 (hexane/ethyl acetate 2/3). ^1^H NMR (400 MHz, CDCl_3_) δ: 7.56–7.51 (m, 2H), 7.32–7.28 (m, 2H), 3.08 (s, 3H), 2.99 (s, 3H). ^13^C{^1^H} NMR (100 MHz, CDCl_3_) δ: 170.6, 135.0, 131.6, 128.8, 123.8, 39.5, 35.4.

#### *N*,*N*-Dimethyl-4-(trifluoromethyl)benzamide (**3ak**)^19^

Yellow solid (198 mg, 91% yield); mp 95–96 °C, *R*_*f*_ = 0.26 (hexane/ethyl acetate 2/3). ^1^H NMR (400 MHz, CDCl_3_) δ: 7.67 (d, *J* = 8.1 Hz, 2H), 7.53 (d, *J* = 8.0 Hz, 2H), 3.13 (s, 3H), 2.96 (s, 3H). ^13^C{^1^H} NMR (100 MHz, CDCl_3_) δ: 170.2, 139.8, 131.5 (q, *J* = 32.6 Hz), 127.4, 125.5 (q, *J* = 3.4 Hz), 123.7 (q, *J* = 266.3 Hz), 39.4, 35.3.

#### Morpholino(phenyl)methanone (**3al**)^18^

Yellow oil (115 mg, 60% yield); *R*_*f*_ = 0.25 (hexane/ethyl acetate 2/3). ^1^H NMR (400 MHz, CDCl_3_) δ: 7.42–7.39 (m, 5H), 3.74–3.45 (m, 8H). ^13^C{^1^H} NMR (100 MHz, CDCl_3_) δ: 170.4, 135.3, 129.8, 128.5, 127.0, 66.9, 48.2, 42.6.

#### (4-Methoxyphenyl)(morpholino)methanone (**3am**)^26^

Yellow oil (212 mg, 96% yield); *R*_*f*_ = 0.16 (hexane/ethyl acetate 2/3). ^1^H NMR (400 MHz, CDCl_3_) δ: 7.37 (d, *J* = 8.7 Hz, 2H), 6.90 (d, *J* = 8.7 Hz, 2H), 3.82 (s, 3H), 3.68–3.62 (m, 8H). ^13^C{^1^H} NMR (100 MHz, CDCl_3_) δ: 170.4, 160.9, 129.1, 127.3, 113.7, 66.9, 55.3, 47.8, 43.9.

#### [1,1’-Biphenyl]-4-yl(morpholino)methanone (**3an**)^27^

White solid (227 mg, 85% yield); mp 86–87 °C, *R*_*f*_ = 0.25 (hexane/ethyl acetate 2.5/2.5). ^1^H NMR (400 MHz, CDCl_3_) δ: 7.64 (d, *J* = 8.3 Hz, 2H), 7.61–7.56 (m, 2H), 7.47–7.44 (m, 4H), 7.40–7.36 (m, 1H), 3.73–3.55 (m, 8H). ^13^C{^1^H} NMR (100 MHz, CDCl_3_) δ: 170.3, 142.9, 140.2, 134.0, 128.9, 127.9, 127.7, 127.3, 127.1, 66.9.

#### Morpholino(1*H*-pyrrol-2-yl)methanone (**3ao**)

White solid (133 mg, 74% yield); mp 73–74 °C, *R*_*f*_ = 0.21 (hexane/ethyl acetate 3/2). ^1^H NMR (400 MHz, CDCl_3_) δ: 9.93 (brs, 1H), 6.92 (td, *J* = 2.7, 1.3 Hz, 1H), 6.51 (ddd, *J* = 3.8, 2.5, 1.3 Hz, 1H), 6.23 (dt, *J* = 3.8, 2.6 Hz, 1H), 3.86 (t, *J* = 4.9 Hz, 4H), 3.75–3.72 (m, 4H). ^13^C{^1^H} NMR (100 MHz, CDCl_3_) δ: 161.9, 124.1, 121.3, 112.2, 109.5, 66.8, 45.2. HRMS (HESI-FT-ORBITRAP) *m*/*z*: [M + H]^+^ calcd for C_9_H_13_N_2_O_2_: 181,0972; found 181,0972.

#### Furan-2-yl(morpholino)methanone (**3ap**)^28^

Yellow oil (27 mg, 15% yield); *R*_*f*_ = 0.26 (hexane/ethyl acetate 2.5/2.5). ^1^H NMR (400 MHz, CDCl_3_) δ: 7.48 (s, 1H), 7.03 (d, *J* = 3.5 Hz, 1H), 6.49–6.48 (m, 1H), 3.85–3.72 (m, 8H). ^13^C NMR (100 MHz, CDCl_3_) δ: 159.1, 147.7, 143.8, 116.8, 111.4, 67.0.

#### Morpholino(thiophen-2-yl)methanone (**3aq**)^29^

Yellow oil (138 mg, 70% yield); *R*_*f*_ = 0.35 (hexane/ethyl acetate 2.5/2.5). ^1^H NMR (400 MHz, CDCl_3_) δ: 7.45 (d, *J* = 4.8 Hz, 1H), 7.28 (d, *J* = 3.5 Hz, 1H), 7.06–7.02 (m, 1H), 3.77–3.75 (m, 4H), 3.73–3.71 (m, 4H). ^13^C{^1^H} NMR (100 MHz, CDCl_3_) δ:163.6, 136.6, 128.9 128.8, 126.7, 66.8, 45.9.

#### *N*-Benzylthiophene-2-carboxamide (**3ar**)^8c^

White solid (185 mg, 85% yield); mp 114–115 °C, *R*_*f*_ = 0.29 (hexane/ethyl acetate 4/1). ^1^H NMR (400 MHz, CDCl_3_) δ: 7.52 (d, *J* = 3.5 Hz, 1H), 7.47 (d, *J* = 4.9 Hz, 1H), 7.34 (d, *J* = 4.4 Hz, 4H), 7.30 (dd, *J* = 5.0, 3.6 Hz, 1H), 7.09–7.00 (m, 1H), 6.47 (s, 1H), 4.60 (d, *J* = 5.8 Hz, 2H). ^13^C NMR (100 MHz, CDCl_3_) δ 161.9, 138.7, 138.0, 130.0, 128.7, 128.1, 127.9, 127.6, 43.9.

#### Morpholino(*p*-tolyl)methanone (**3as**)^26^

Yellow oil (121 mg, 59% yield); *R*_*f*_ = 0.23 (hexane/ethyl acetate 2/3). ^1^H NMR (400 MHz, CDCl_3_) δ: 7.30 (d, *J* = 8.1 Hz, 2H), 7.20 (d, *J* = 7.8 Hz, 2H), 3.61 (m, 8H), 2.37 (s, 3H). ^13^C{^1^H} NMR (100 MHz, CDCl_3_) δ: 170.6, 140.1, 132.3, 129.1, 127.2, 66.9, 48.3, 42.9, 21.3.

#### (4-Chlorophenyl)(morpholino)methanone (**3at**)^30^

White solid (176 mg, 78% yield); mp 74–76 °C, *R*_*f*_ = 0.26 (hexane/ethyl acetate 2/3). ^1^H NMR (400 MHz, CDCl_3_) δ: 7.36–7.30 (m, 4H), 3.65–3.40 (m, 8H). ^13^C{^1^H} NMR (100 MHz, CDCl_3_) δ: 169.2, 135.8, 133.5, 128.7, 128.5, 66.6, 48.0, 42.6.

#### (3-Chlorophenyl)(morpholino)methanone (**3au**)^31^

Brown oil (176 mg, 78% yield); *R*_*f*_ = 0.48 (hexane/ethyl acetate 2/3). ^1^H NMR (400 MHz, CDCl_3_) δ: 7.43–7.23 (m, 3H), 7.21–7.19 (m, 1H), 3.86–3.18 (m, 8H). ^13^C{^1^H} NMR (100 MHz, CDCl_3_) δ: 168.8, 137.0, 134.7, 130.0, 129.9, 127.2, 125.1, 66.8, 48.1, 42.6.

#### *N*-Benzylbenzamide (**3av**)^32^

Brown solid (167 mg, 79% yield); mp 104–105 °C, *R*_*f*_ = 0.42 (hexane/ethyl acetate 3.5/1.5). ^1^H NMR (400 MHz, CDCl_3_) δ: 7.79 (d, *J* = 7.3 Hz, 2H), 7.48 (d, *J* = 7.4 Hz, 1H), 7.40 (t, *J* = 7.5 Hz, 2H), 7.34 (d, *J* = 4.4 Hz, 4H), 7.31–7.26 (m, 1H), 6.68 (s, 1H), 4.62 (d, *J* = 5.7 Hz, 2H). ^13^C{^1^H} NMR (100 MHz, CDCl_3_) δ: 167.4, 138.2, 134.3, 131.5, 128.7, 128.5, 127.8, 127.5, 126.9, 44.0.

#### *N*-(4-Bromobenzyl)benzamide (**3aw**)^33^

White solid (58 mg, 20% yield); mp 72–74 °C, *R*_*f*_ = 0.28 (hexane/ethyl acetate 4.5/0.5). ^1^H NMR (400 MHz, CDCl_3_) δ: 7.81–7.75 (m, 2H), 7.52–7.41 (m, 5H), 7.24 (d, *J* = 8.4 Hz, 2H), 6.43 (s, 1H), 4.60 (d, *J* = 5.7 Hz, 2H). ^13^C{^1^H} NMR (100 MHz, CDCl_3_) δ: 167.4, 137.3, 134.1, 131.8, 131.7, 129.5, 128.6, 126.9, 121.4, 43.4.

#### *N*-(4-Bromobenzyl)-4-methoxybenzamide (**3ax**)^34^

Yellow solid (186 mg, 58% yield); mp 176–177 °C, *R*_*f*_ = 0.28 (hexane/ethyl acetate 3/2). ^1^H NMR (400 MHz, CDCl_3_) δ: 7.75 (d, *J* = 8.8 Hz, 2H), 7.46 (d, *J* = 8.4 Hz, 2H), 7.22 (d, *J* = 8.4 Hz, 2H), 6.92 (d, *J* = 8.8 Hz, 2H), 6.36 (s, 1H), 4.58 (d, *J* = 5.7 Hz, 2H), 3.85 (s, 3H). ^13^C{^1^H} NMR (100 MHz, CDCl_3_) δ: 166.9, 162.3, 137.5, 131.8, 129.5, 128.8, 126.4, 121.4, 113.8, 55.4, 43.4.

#### *N*-(2-Chloroethyl)-[1,1’-biphenyl]-4-carboxamide (**3ay**)

Yellow solid (179 mg, 69% yield); mp 123–124 °C,^35^*R*_*f*_ = 0.28 (hexane/ethyl acetate 4.5/0.5). ^1^H NMR (400 MHz, CDCl_3_) δ: 7.87 (d, *J* = 8.4 Hz, 2H), 7.67 (d, *J* = 8.4 Hz, 2H), 7.66–7.61 (m, 2H), 7.47 (t, *J* = 7.5 Hz, 2H), 7.41–7.37 (m, 1H), 6.61 (s, 1H), 3.87–3.81 (m, 2H), 3.79–3.74 (m, 2H).^35^^13^C{^1^H} NMR (100 MHz, CDCl_3_) δ: 167.3, 144.6, 139.9, 128.9, 128.1, 127.5, 127.3, 127.2, 44.2, 41.7. HRMS (HESI-FT-ORBITRAP) *m*/*z*: [M + H]^+^ calcd for C_15_H_15_ClNO: 260,0837; found 260,0835.

#### *N*-Benzyl-4-chlorobenzamide (**3az**)^36^

White solid (184 mg, 75% yield); mp 139–141 °C, *R*_*f*_ = 0.38 (hexane/ethyl acetate 3.7/1.3). ^1^H NMR (400 MHz, CDCl_3_) δ: 7.73 (d, *J* = 8.6 Hz, 2H), 7.40 (d, *J* = 8.6 Hz, 2H), 7.36–7.32 (m, 5H), 6.40 (s, 1H), 4.63 (d, *J* = 5.7 Hz, 2H). ^13^C{^1^H} NMR (100 MHz, CDCl_3_) δ: 166.3, 137.9, 137.8, 132.7, 128.8, 128.4, 127.9, 127.7, 44.3.

#### *N-*(2-Chloroethyl)-4-nitrobenzamide (**3ba**)^37^

White solid (110 mg, 48% yield); mp 123–124 °C,^38^*R*_*f*_ = 0.29 (hexane/ethyl acetate 3/2). ^1^H NMR (400 MHz, CDCl_3_) δ: 8.30 (d, *J* = 8.8 Hz, 2H), 7.96 (d, *J* = 8.8 Hz, 2H), 6.66 (s, 1H), 3.87–3.81 (m, 2H), 3.79–3.74 (m, 2H). ^13^C{^1^H} NMR (100 MHz, CDCl_3_) δ: 165.6, 149.8, 139.6, 128.2, 123.9, 43.8, 41.9.

#### *N*-(*tert*-Butyl)benzamide (**3bb**)^18^

Yellow solid (105 mg, 59% yield); mp 134–135 °C, *R*_*f*_ = 0.354 (hexane/ethyl acetate 4/1). ^1^H NMR (400 MHz, CDCl_3_) δ: 7.71 (d, *J* = 6.9 Hz, 2H), 7.45 (d, *J* = 7.2 Hz, 1H), 7.40 (t, *J* = 7.2 Hz, 2H), 5.96 (s, 1H), 1.47 (s, 9H). ^13^C{^1^H} NMR (100 MHz, CDCl_3_) δ: 166.9, 135.9, 131.0, 128.4, 126.7, 51.6, 28.8.

### General Procedure to Compounds **3bc**

To a mixture of NH_4_Cl (96.3 mg, 1.8 mmol), Ru(bpy)_3_Cl_2_*6H_2_O (37.4 mg, 0.05 mmol, 5.0 mol %), and CaCO_3_ (110 mg, 1.1 mmol) in CH_3_CN (0.5 mL) were added benzyl alcohol (108.1 mg, 1 mmol) and *tert*-butyl hydroperoxide (70 wt % in H_2_O, 0.31 mL, 2.2 mmol) under an argon atmosphere at room temperature. The reaction vessel was capped, stirred, and irradiated under blue LED at room temperature for 72 h (monitored by TLC until disappearance of the alcohol). Then, the reaction mixture was evaporated under reduced pressure. The crude products were purified by flash chromatography on silica gel.

#### Benzamide (**3bc**)

White solid (97 mg, 80% yield); mp 126–127 °C,^39^*R*_*f*_ = 0.4 (hexane/ethyl acetate 1.5/3.5). ^1^H NMR (400 MHz, CDCl_3_) δ: 7.83 (d, *J* = 7.0 Hz, 2H), 7.53 (t, *J* = 7.4 Hz, 1H), 7.44 (t, *J* = 7.5 Hz, 2H), 6.46 (brs, 2H).^40^^13^C{^1^H} NMR (100 MHz, CDCl_3_) δ: 169.9, 132.9, 132.2, 128.6, 127.4.^40^

The reaction was investigated with aliphatic aldehydes:

### General Procedure to Compounds **4a**–**4c**

To a mixture of amine hydrochloride salt (1.2 mmol), Ru(bpy)_3_Cl_2_*6H_2_O (37.4 mg, 0.05 mmol, 5.0 mol %), and CaCO_3_ (110 mg, 1.1 mmol) in ethyl acetate (0.5 mL) were added aliphatic aldehyde (1 mmol) and *tert*-butyl hydroperoxide (70 wt % in H_2_O, 0.156 mL, 1.1 mmol) under an argon atmosphere at room temperature. The reaction mixture was irradiated under blue LED and stirred at room temperature for 48 h (monitored by TLC until disappearance of the alcohol). Then, the reaction mixture was quenched with water and extracted with AcOEt. The combined organic layers were washed three times with a solution of 5% citric acid and then three times with a solution of 5% NaHCO_3_; the organic phase was dried over anhydrous Na_2_SO_4_, and the solvent was evaporated under reduced pressure. The crude products were purified by flash chromatography on silica gel.

### Compound Characterizations

#### *N-*(4-Bromobenzyl)-3-phenylpropanamide (**4a**)

Yellow oil (63.6 mg, 20% yield); *R*_*f*_ = 0.33 (hexane/ethyl acetate 2.5/2.5). ^1^H NMR (400 MHz, CDCl_3_) δ: 7.41 (d, *J* = 8.1 Hz, 2H), 7.29 (d, *J* = 7.0 Hz, 2H), 7.22 (dd, *J* = 16.2, 7.1 Hz, 3H), 6.99 (d, *J* = 8.1 Hz, 2H), 5.83 (s, 1H), 4.34 (d, *J* = 5.4 Hz, 2H), 3.00 (t, *J* = 7.4 Hz, 2H), 2.54 (t, *J* = 7.5 Hz, 2H). ^13^C{^1^H} NMR (100 MHz, CDCl_3_) δ: 172.0, 140.5, 137.2, 131.6, 129.3, 128.5, 128.4, 126.3, 121.2, 42.8, 38.4, 31.6. *m*/*z*: [M + H]^+^ calcd for C_16_H_17_BrNO: 318,0488; found 318,0491.

#### *N*-(2-Bromobenzyl)isobutyramide (**4b**)^41^

Colorless oil (61.5 mg, 24% yield); *R*_*f*_ = 0.313 (hexane/ethyl acetate 3.5/1.5). ^1^H NMR (400 MHz, CDCl_3_) δ: 7.55 (d, *J* = 7.8 Hz, 1H), 7.36 (dd, *J* = 7.6, 1.8 Hz, 1H), 7.28 (t, *J* = 7.2 Hz, 1H), 7.14 (td, *J* = 7.7, 1.8 Hz, 1H), 6.16 (t, *J* = 6.3 Hz, 1H), 4.49 (d, *J* = 6.0 Hz, 2H), 2.45–2.38 (m, 1H), 1.17 (d, *J* = 6.9 Hz, 6H). ^13^C {^1^H} NMR (100 MHz, CDCl_3_) δ: 176.8, 137.4, 132.7, 130.1, 129.0, 127.6, 123.6, 43.6, 35.5, 19.5.

#### *N*-Benzylcyclohexanecarboxamide (**4c**)^42^

White solid (99.9 mg, 46% yield); mp 112–114 °C, *R*_*f*_ = 0.44 (hexane/ethyl acetate 3.5/1.5). ^1^H NMR (400 MHz, CDCl_3_) δ: 7.26–7.17 (m, 5H), 5.82 (s, 1H), 4.34 (d, *J* = 5.6 Hz, 2H), 2.09–1.99 (m, 1H), 1.81 (dd, *J* = 13.1, 3.6 Hz, 2H), 1.72–1.68 (m, 2H), 1.62–1.55 (m, 1H), 1.45–1.33 (m, 2H), 1.22–1.13 (m, 3H). ^13^C NMR (100 MHz, CDCl_3_) δ 176.0, 138.5, 128.6, 127.6, 127.4, 45.5, 43.3, 29.7, 25.7.

### Scale-Up for the Synthesis of **3aa**

To a mixture of dimethylamine hydrochloride (0.9 g, 11.04 mmol), Ru(bpy)_3_Cl_2_*6H_2_O (0.34 g, 0.46 mmol, 5.0 mol %), and CaCO_3_ (1.0 g, 10.12 mmol) in ethyl acetate (5 mL) were added benzyl alcohol (1.0 g, 0.95 mL, 9.2 mmol) and *tert*-butyl hydroperoxide (70 wt % in H_2_O, 2.80 mL, 20.2 mmol) under an argon atmosphere at room temperature. The reaction mixture was irradiated under blue LED and stirred at room temperature for 72 h (monitored by TLC until disappearance of the alcohol). Then, the reaction mixture was quenched with water and extracted with AcOEt. The combined organic layers were washed three times with a solution of 5% citric acid and then three times with a solution of 5% NaHCO_3_; the organic phase was dried over anhydrous Na_2_SO_4_, and the solvent was evaporated under reduced pressure. The crude product was purified by flash chromatography on silica gel, and the product **3aa** was obtained as a yellow oil (822 mg, 60% yield).

### Determination of Reaction Quantum Yield

The quantum yield calculation of the reaction was determined in two steps:^16^

#### Determination of the Light Intensity Obtained from the Blue LED

The photon flux of the blue LED was determined by the standard potassium ferrioxalate actinometer method.^43^

For the evaluation of light intensity, an experiment was set by preparing a 0.15 M solution of ferrioxalate actinometer by dissolving 0.737 g of potassium trisoxalato ferrate trihydrate complex [K_3_Fe(C_2_O_4_)_3_]*3H_2_O in 10 mL of a 0.05 M H_2_SO_4_ solution. A buffered solution of 1,10-phenanthroline was prepared by dissolving 0.025 g of 1,10-phenanthroline and 5.63 g of sodium acetate in 25 mL of a 0.5 M solution H_2_SO_4_. Both solutions were stored in the dark.

For the actinometer measurement:

Ferrioxalate solution (2.0 mL) was placed in a cuvette and irradiated under blue LED for 90 s. After irradiation, 0.35 mL of the phenanthroline solution was added to the ferrioxalate solution and the mixture was stirred in the dark for 1.0 h to allow the ferrous ions to completely coordinate to the phenanthroline.

The absorbance of the solution at λ = 510 nm was measured. A similar procedure for a non irradiated sample was used (actinometer solution, buffer, and phenanthroline), and its absorbance at λ = 510 nm was measured. Conversion was determined according to the Beer’s laws using equation
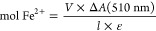

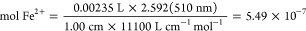
where *V* is the total volume of the solution (0.00235 L) after addition of all reagents, Δ*A* is the difference in absorbance at λ = 510 nm between the irradiated and nonirradiated actinometer solutions (2.924–0.332), l is the path length (1.00 cm), and ε is the molar absorptivity of the ferrioxalate actinometer at λ 510 nm (11100 L cm^–1^ mol^–1^).^44^ The photon flux of the blue LED can be calculated using the following equation
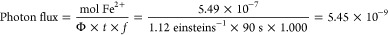
where Φ is the quantum yield for the ferrioxalate actinometer (1.12 einsteins^–1^),^43b^*t* is the irradiation time (90 s), and *f* is the fraction of light absorbed by the ferrioxalate actinometer. An absorption spectrum gave an absorbance value of >3, indicating that the fraction of absorbed light *f* is > 0.999. The photon flux was thus calculated to be 5.45 × 10^–9^ einsteins s^–1^.

#### Determination of the Reaction Quantum Yield

To a mixture of dimethylamine hydrochloride (97.8 mg, 1.2 mmol), Ru(bpy)_3_Cl_2_*6H_2_O (37.4 mg, 0.05 mmol, 5.0 mol %), and CaCO_3_ (110 mg, 1.1 mmol) in ethyl acetate (0.5 mL) were added benzyl alcohol (108 mg, 1 mmol) and *tert*-butyl hydroperoxide (70 wt % in H_2_O, 0.31 mL, 2.2 mmol) under an argon atmosphere at room temperature. The reaction mixture was irradiated under blue LED and stirred at room temperature for 180 s. After irradiation, the yield of product **3aa** formed was determined by ^1^H NMR analysis. The yield of **3aa** was determined to be 5% (5 × 10^–5^ mol). The reaction quantum yield (Φ) was determined using the following equation, where the photon flux is 5.45 × 10^–9^ einsteins s^–1^ (determined by actinometry as described in step 1), *t* is the reaction time (180 s), and *f* is the fraction of incident light absorbed by the reaction mixture.
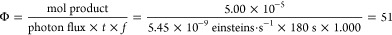
The reaction quantum yield (Φ) was thus determined to be 51.

## Supporting Information Available

Optimization of reaction conditions; experimental setup photograph; UV–vis, ^1^H NMR, and ^13^C NMR spectra of products (PDF)

## References

[ref1] aPattabiraman V. R.; Bode J. W. Rethinking amide bond synthesis. Nature 2011, 480, 471–479. 10.1038/nature10702. 22193101

[ref2] Valeur E.; Bradley M. Amide bond formation: beyond the myth of coupling reagents. Chem. Soc. Rev. 2009, 38, 606–631. 10.1039/B701677H. 19169468

[ref3] aOhkubo K.; Fujimoto A.; Fukuzumi S. Aromatic Monochlorination Photosensitized by DDQ with Hydrogen Chloride under Visible-Light Irradiation. Chem. Asian J. 2016, 11, 996–999. 10.1002/asia.201600083. 26892685

[ref4] aPrier C. K.; Rankic D. A.; MacMillan D. W. Visible Light Photoredox Catalysis with Transition Metal Complexes: Applications in Organic Synthesis. Chem. Rev. 2013, 113, 5322–5363. 10.1021/cr300503r. 23509883 PMC4028850

[ref5] Zhang Y.; Riemer D.; Schilling W.; Kollmann J.; Das S. Visible-Light-Mediated Efficient Metal-Free Catalyst for α-Oxygenation of Tertiary Amines to Amides. ACS Catal. 2018, 8, 6659–6664. 10.1021/acscatal.8b01897.

[ref6] aCohen I.; Mishra A. K.; Parvari G.; Edrei R.; Dantus M.; Eichen Y.; Szpilman A. M. Sunlight assisted direct amide formation via a charge-transfer complex. Chem. Commun. 2017, 53, 10128–10131. 10.1039/C7CC05300B. 28841217

[ref7] Pandey G.; Koley S.; Talukdar R.; Sahani P. K. Cross-Dehydrogenating Coupling of Aldehydes with Amines/R-OTBS Ethers by Visible-Light Photoredox Catalysis: Synthesis of Amides, Esters, and Ureas. Org. Lett. 2018, 20, 5861–5865. 10.1021/acs.orglett.8b02537. 30192550

[ref8] aGaspa S.; Porcheddu A.; Luca L. De. Metal-Free Oxidative Cross Esterification of Alcohols via Acyl Chloride Formation. Adv. Synth. Catal. 2016, 358, 154–158. 10.1002/adsc.201500912.

[ref9] Yang T.; Lu M.; Lin Z.; Huang M.; Cai S. Visible-light-promoted oxidation/condensation of benzyl alcohols with dialkylacetamides to cinnamides. Org. Biomol. Chem. 2019, 17, 449–453. 10.1039/C8OB02938E. 30564808

[ref10] aCadoni R.; Porcheddu A.; Giacomelli G.; De Luca L. One-Pot Synthesis of Amides from Aldehydes and Amines via C–H Bond Activation. Org. Lett. 2012, 14, 5014–5017. 10.1021/ol302175v. 22978698

[ref11] Prat D.; Pardigon O.; Flemming H.-W.; Letestu S.; Ducandas V.; Isnard P.; Guntrum E.; Senac T.; Ruisseau S.; Cruciani P.; Hasek P. Sanofi’s Solvent Selection Guide: A Step Toward More Sustainable Processes. Org. Process Res. Dev. 2013, 17, 1517–1525. 10.1021/op4002565.

[ref12] Henderson R. K.; Jiménez-Gonzalez C.; Constable D. J. C.; Alston S. R.; Inglis G. G. A.; Fisher G.; Sherwood J.; Binks S. P.; Curzons A. D. Expanding GSKʼs solvent selection guide – embedding sustainability into solvent selection starting at medicinal chemistry. Green Chem. 2011, 13, 854–862. 10.1039/c0gc00918k.

[ref13] See the Experimental Section.

[ref14] aChoudhary V. R.; Dumbre D. K.; Uphade B. S.; Narkhede V. S. Solvent-free oxidation of benzyl alcohol to benzaldehyde by tert-butyl hydroperoxide using transition metal containing layered double hydroxides and/or mixed hydroxides. J. Mol. Catal. A: Chem. 2004, 215, 129–135. 10.1016/j.molcata.2004.01.009.

[ref15] Ekoue-Kovi K.; Wolf C. Metal-Free One-Pot Oxidative Amination of Aldehydes to Amides. Org. Lett. 2007, 9, 3429–3432. 10.1021/ol7014626. 17655318

[ref16] aShort M. A.; Blackburn J. M.; Roizen J. L. Sulfamate Esters Guide Selective Radical-Mediated Chlorination of Aliphatic C-H Bonds. Angew. Chem., Int. Ed. 2018, 57, 296–299. 10.1002/anie.201710322. PMC5745255 29096044

[ref17] Xia Q.; Liu X.; Zhang Y.; Chen C.; Chen W. Copper-Catalyzed N-Methylation of Amides and O-Methylation of Carboxylic Acids by Using Peroxides as the Methylating Reagents. Org. Lett. 2013, 15, 3326–3329. 10.1021/ol401362k. 23789961

[ref18] Zhao Q.; Li H.; Wang L. The direct amidation of α-diketones with amines via TBHP-promoted oxidative cleavage of C(sp^2^)–C(sp^2^) bonds. Org. Biomol. Chem. 2013, 11, 6772–6779. 10.1039/c3ob41392f. 23999992

[ref19] aOng D. Y.; Yen Z.; Yoshii A.; Revillo Imbernon J.; Takita R.; Chiba S. Controlled Reduction of Carboxamides to Alcohols or Amines by Zinc Hydrides. Angew. Chem., Int. Ed. 2019, 58, 4992–4997. 10.1002/anie.201900233. 30761712

[ref20] Ju J.; Jeong M.; Moon J.; Jung H. M.; Lee S. Aminocarbonylation of Aryl Halides Using a Nickel Phosphite Catalytic System. Org. Lett. 2007, 9, 4615–4618. 10.1021/ol702058e. 17915887

[ref21] Bannwart L.; Abele S.; Tortoioli S. Metal-Free Amidation of Acids with Formamides and T3P. Synthesis 2016, 48, 2069–2078. 10.1055/s-0035-1561427.

[ref22] aAsghar S.; Tailor S. B.; Elorriaga D.; Bedford R. B. Cobalt-Catalyzed Suzuki Biaryl Coupling of Aryl Halides. Angew. Chem., Int. Ed. 2017, 56, 16367–16370. 10.1002/anie.201710053. PMC5767760 29135071

[ref23] Wang L.; Ackermann L. Ruthenium-catalyzed *ortho*-C–H halogenations of benzamides. Chem. Commun. 2014, 50, 1083–1085. 10.1039/C3CC47852A. 24317223

[ref24] Kumar P. S.; Kumar G. S.; Kumar R. A.; Reddy N. V.; Reddy K. R. Copper-Catalyzed Oxidative Coupling of Carboxylic Acids with *N,N*-Dialkylformamides: An Approach to the Synthesis of Amides. Eur. J. Org. Chem. 2013, 2013, 1218–1222. 10.1002/ejoc.201201544.

[ref25] Bao Y.-S.; Wang L.; Jia M.; Xu A.; Agula B.; Baiyin M.; Zhaorigetu B. Heterogeneous recyclable nano-palladium catalyzed amidation of esters using formamides as amine sources. Green Chem. 2016, 18, 3808–3814. 10.1039/C5GC02985F.

[ref26] Wang T.; Yuan L.; Zhao Z.; Shao A.; Gao M.; Huang Y.; Xiong F.; Zhang H.; Zhao J. Direct oxidative amidation between methylarenes and amines in water. Green Chem. 2015, 17, 2741–2744. 10.1039/C5GC00299K.

[ref27] Hua X.; Masson-Makdissi J.; Sullivan R. J.; Newman S. G. Inherent vs Apparent Chemoselectivity in the Kumada–Corriu Cross-Coupling Reaction. Org. Lett. 2016, 18, 5312–5315. 10.1021/acs.orglett.6b02631. 27696884

[ref28] Zhu J.; Zhang Y.; Shi F.; Deng Y. Dehydrogenative amide synthesis from alcohol and amine catalyzed by hydrotalcite-supported gold nanoparticles. Tetrahedron Lett. 2012, 53, 3178–3180. 10.1016/j.tetlet.2012.04.048.

[ref29] Rahman M. M.; Li G.; Szostak M. Metal-Free Transamidation of Secondary Amides by N – C Cleavage. J. Org. Chem. 2019, 84, 12091–12100. 10.1021/acs.joc.9b02013. 31430149

[ref30] Rushworth P. J.; Hulcoop D. G.; Fox D. J. Iron/Tetramethylethylenediamine-Catalyzed Ambient-Temperature Coupling of Alkyl Grignard Reagents and Aryl Chlorides. J. Org. Chem. 2013, 78, 9517–9521. 10.1021/jo4016612. 23984915

[ref31] Pilo M.; Porcheddu A.; Luca L. De. A copper-catalysed amidation of aldehydes via N-hydroxysuccinimide ester formation. Org. Biomol. Chem. 2013, 11, 8241–8246. 10.1039/c3ob41440j. 24165994

[ref32] Rolfe A.; Loh J. K.; Maity P. K.; Hanson P. R. High-load, hybrid Si-ROMP reagents. Org. Lett. 2011, 13, 4–7. 10.1021/ol102239h. 21128690 PMC3271940

[ref33] Zeng H.-T.; Huang J.-M. Copper-Catalyzed Ligand-Free Amidation of Benzylic Hydrocarbons and Inactive Aliphatic Alkanes. Org. Lett. 2015, 17, 4276–4279. 10.1021/acs.orglett.5b02063. 26295495

[ref34] Myers R. M.; Langston S. P.; Conway S. P.; Abell C. Reductive Cleavage of N–O Bonds Using Samarium(II) Iodide in a Traceless Release Strategy for Solid-Phase Synthesis. Org. Lett. 2000, 2, 1349–1352. 10.1021/ol0055162. 10814444

[ref35] Paudel S.; Cao Y.; Guo S.; An B.; Kim K.-M.; Cheon S. H. Design and synthesis of 4-benzylpiperidine carboxamides as dual serotonin and norepinephrine reuptake inhibitors. Bioorg. Med. Chem. 2015, 23, 6418–6426. 10.1016/j.bmc.2015.08.022. 26337019

[ref36] Howard E.-L.; Guzzardi N.; Tsanova V. G.; Stika A.; Patel B. Highly Efficient Copper-Catalyzed Amidation of Benzylic Hydrocarbons Under Neutral Conditions. Eur. J. Org. Chem. 2018, 2018, 794–797. 10.1002/ejoc.201701759.

[ref37] Kawagoe K.; Motoki K.; Odagiri T.; Suzuki N.; Chen C.-J.; Mimura T. Vol. WO2004/087641, 2006.

[ref38] Ben-Ishai D. The Reactions of β-Hydroxyethylamides and β-Hydroxyethylcarbamates with Phosgene. J. Am. Chem. Soc. 1956, 78, 4962–4965. 10.1021/ja01600a042.

[ref39] Vadagaonkar K. S.; Kalmode H. P.; Prakash S.; Chaskar A. C. Iodine-Mediated Domino Protocol for the Synthesis of Benzamides from Ethylarenes via sp^3^ C–H Functionalization. Synlett 2015, 26, 1677–1682. 10.1055/s-0034-1380210.

[ref40] Wu C.; Xin X.; Fu Z.-M.; Xie L.-Y.; Liu K.-J.; Wang Z.; Li W.; Yuan Z.-H.; He W.-M. Water-controlled selective preparation of α-mono or α,α′-dihalo ketones via catalytic cascade reaction of unactivated alkynes with 1,3-dihalo-5,5-dimethylhydantoin. Green Chem. 2017, 19, 1983–1989. 10.1039/C7GC00283A.

[ref41] Laidaoui N.; Miloudi A.; El Abed D.; Doucet H. Palladium-Catalysed Direct Heteroarylation of Bromobenzylacetamide Derivatives: A Simple Access to Heteroarylated Benzylamine Derivatives. Synthesis 2010, 12010, 2553–2566. 10.1055/s-0029-1218814.

[ref42] Lee C.-H.; Lee S.-M.; Min B.-H.; Kim D.-S.; Jun C.-H. Ferric (III) chloride catalyzed halogenation reaction of alcohols and carboxylic acids using αα-dichlorodiphenylmethane. Org. Lett. 2018, 20, 2468–2471. 10.1021/acs.orglett.8b00831. 29624066

[ref43] aCismesia M. A.; Yoon T. P. Characterizing chain processes in visible light photoredox catalysis. Chem. Sci. 2015, 6, 5426–5434. 10.1039/C5SC02185E. 26668708 PMC4676763

[ref44] Montalti M.; Credi A.; Prodi L.; Gandolfi M. T. Chemical Actinometry. In Handbook of Photochemistry, 3rd ed.; CRC press, 2006.

