# Supplementary material for: Visible-Light Photoredox-Catalyzed Amidation of Benzylic Alcohols
Source: J Org Chem. 2020 Jul 14;85(18):11679–87. doi: 10.1021/acs.joc.0c01320 (PMC8009506; doi:10.1021/acs.joc.0c01320)

Supporting Information

Visible-light photoredox catalyzed amidation of benzylic alcohols

Silvia Gaspa,[a] Andrea Farina,[a] Mariella Tilocca,[a] Andrea Porcheddu,[b] Luisa Pisano,[a] Massimo Carraro,[a] Ugo Azzena[a] and Lidia De Luca*[a]

a Dipartimento di Chimica e Farmacia, Università degli Studi di Sassari, via Vienna 2, 07100 Sassari, Italy Fax: (+39)-079-229559; phone: (+39)-079-229494; e-mail: ldeluca@uniss.it

b Dipartimento di Scienze Chimiche e Geologiche, Università degli Studi di Cagliari, Cittadella Universitaria, 09042 Monserrato, Italy

Table of contents

1. **Optimization of reaction conditions…………………………………………………….....S4**
2. **Uv-vis spectra ….………………………….…………………………..……………...S5**
3. **Experimental Set-up Photograph…………………………………………………………S6**
4. **NMR Spectra ^{1}H- ^{13}C………..………..…………………..………………………...………S7**

Optimization of reaction conditions

Table S1. Reaction base screening

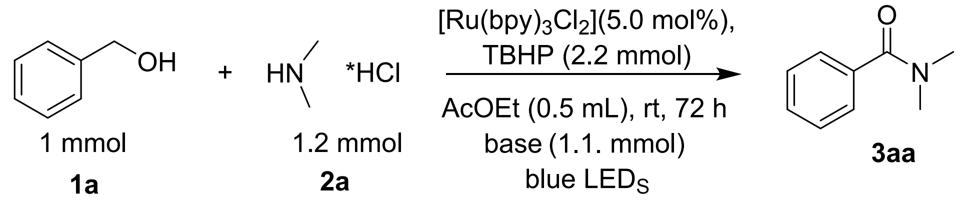

Entry[a]	Base	Yield (%)[b]
1	$CaCO_3$	83
2	NaOH	trace
3	K_2CO_3	trace
4	MgO	30
5	$NaHCO_3$	30
6	Et_3N	trace

[a] General reaction conditions: **1a** (1 mmol), **2a** (1.2 mmol), [Ru(bpy)$_3$Cl$_2$] (5.0 mol%), TBHP$_{aq}$ (2.2 mmol) and base (1.1 mmol) in AcOEt (0.5 mL) at rt in argon for 72 h with 9 W blue LED$_S$.

Uv-vis spectra:

The photocatalyst ($Ru(bpy)_3Cl_2*6H_2O$) concentration was 0.5 mM.

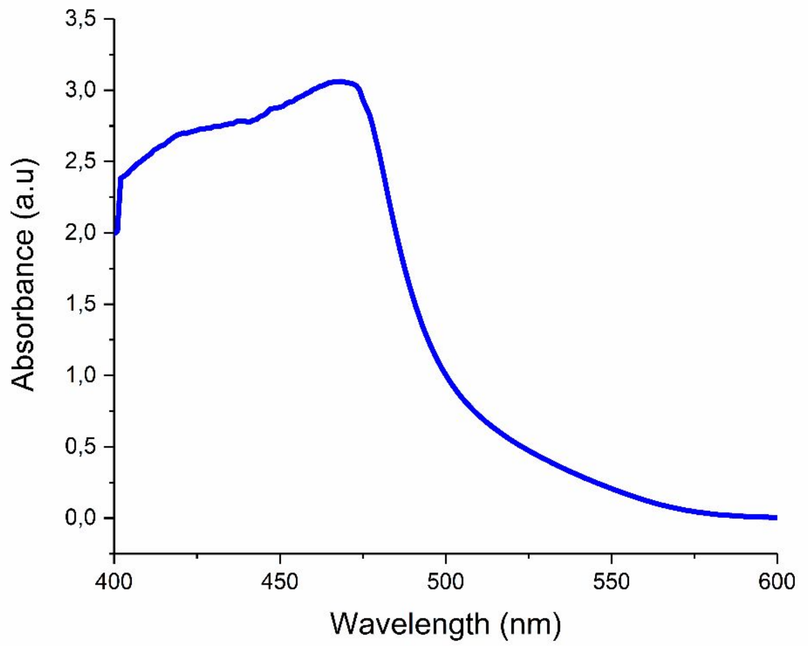

Experimental Set-up Photograph:

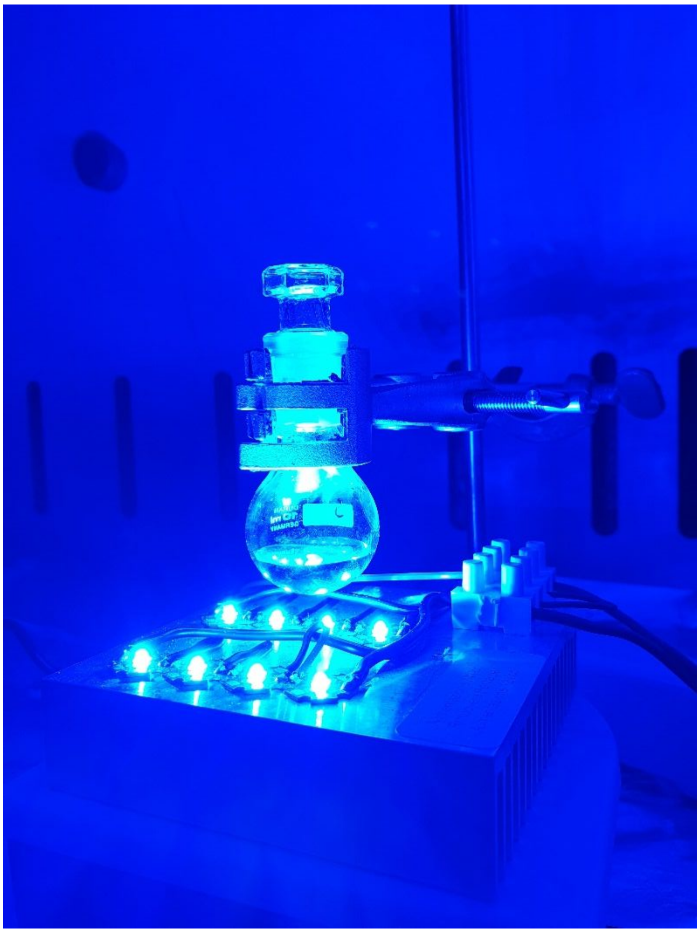

The light source used for photochemical experiments was Blue Led OSRAM Oslon;

Manufacturer: OSRAM Oslon;

Model：Oslon SSL 80 LDCQ7P-1U3U

λ max = 455 nm, I max = 1000 mA, 1.12 W

Material of the irradiation vessel：borosilicate.

Distance from the light source to the irradiation vessel: 2.0 cm

(Not use any filters)

NMR spectra:

***N*,*N*-dimethylbenzamide (3aa):**

^{1}H NMR (400 MHz, $CDCl_3$)

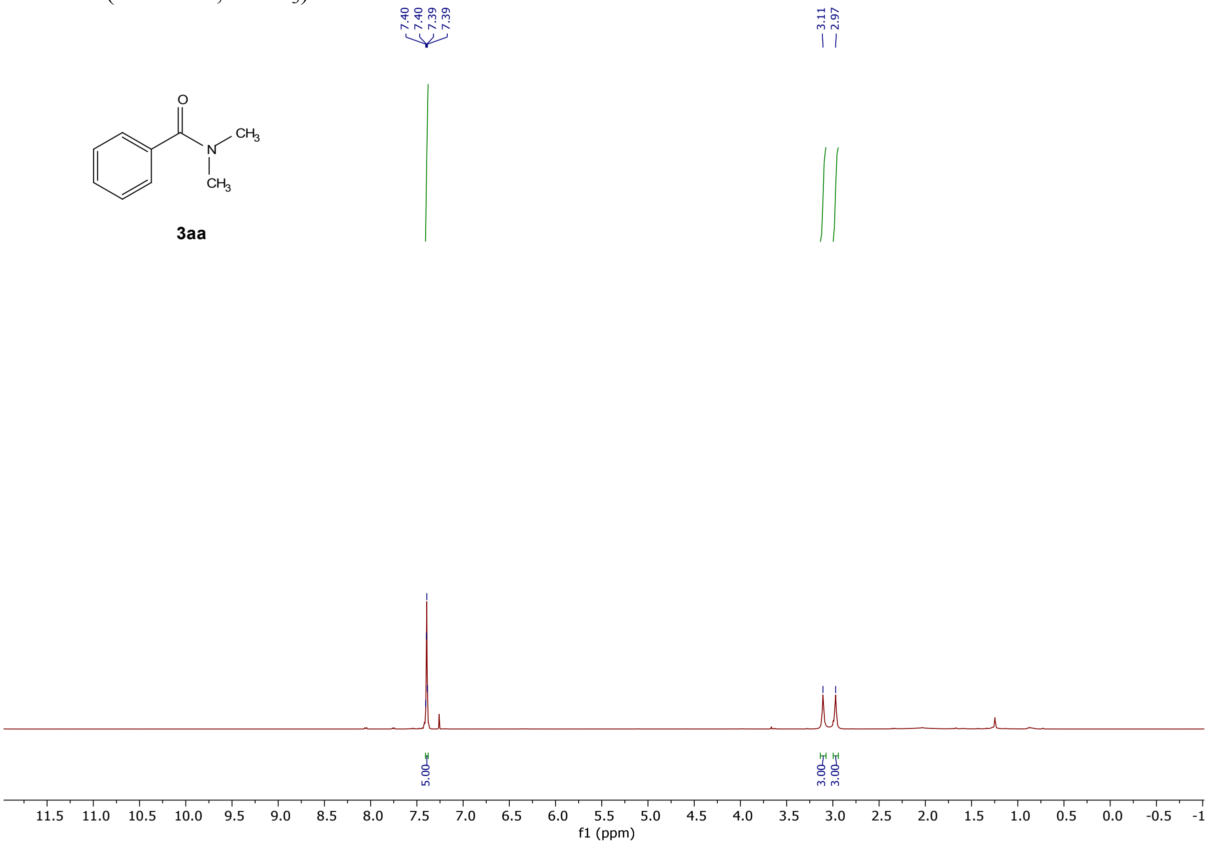

^{13}C{^{1}H} NMR (100 MHz, $CDCl_3$)

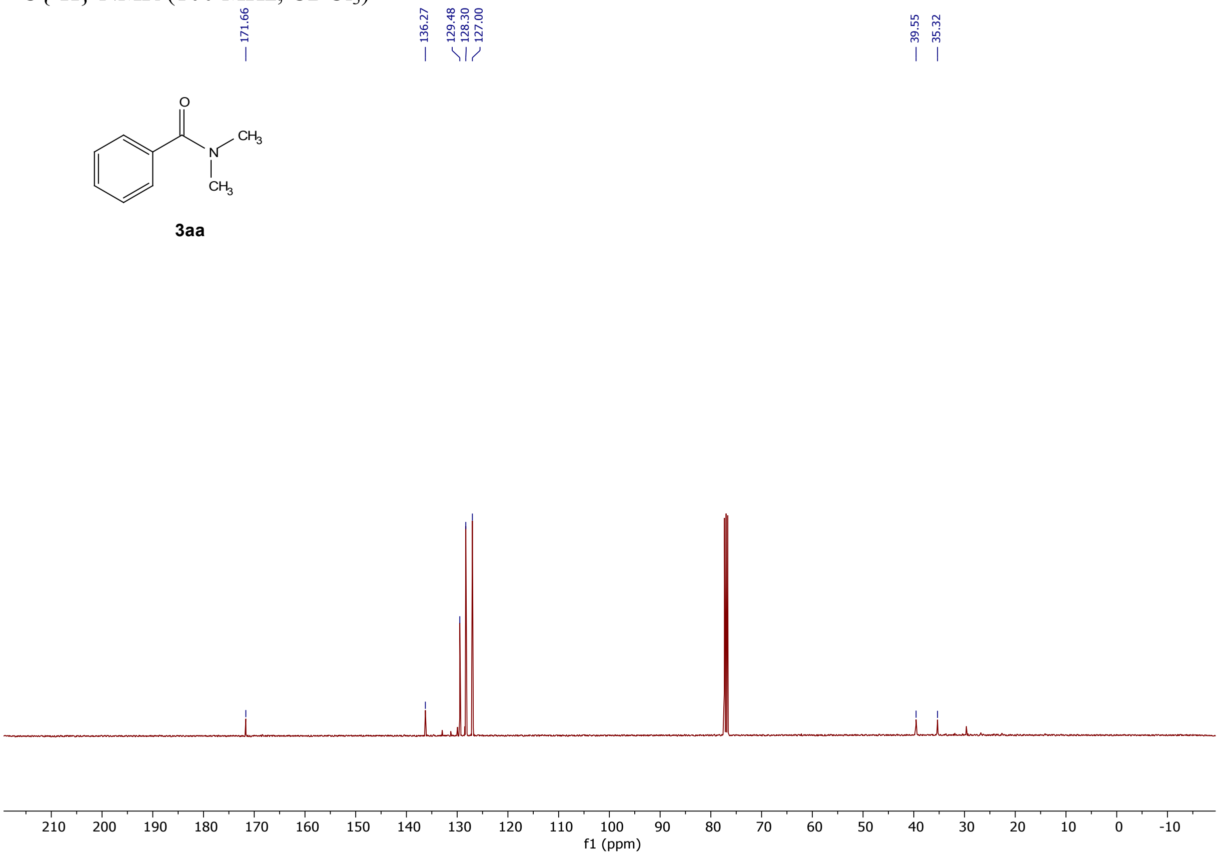

_N,N_-diethylbenzamide (3ab):

^{1}H NMR (400 MHz, $CDCl_3$)

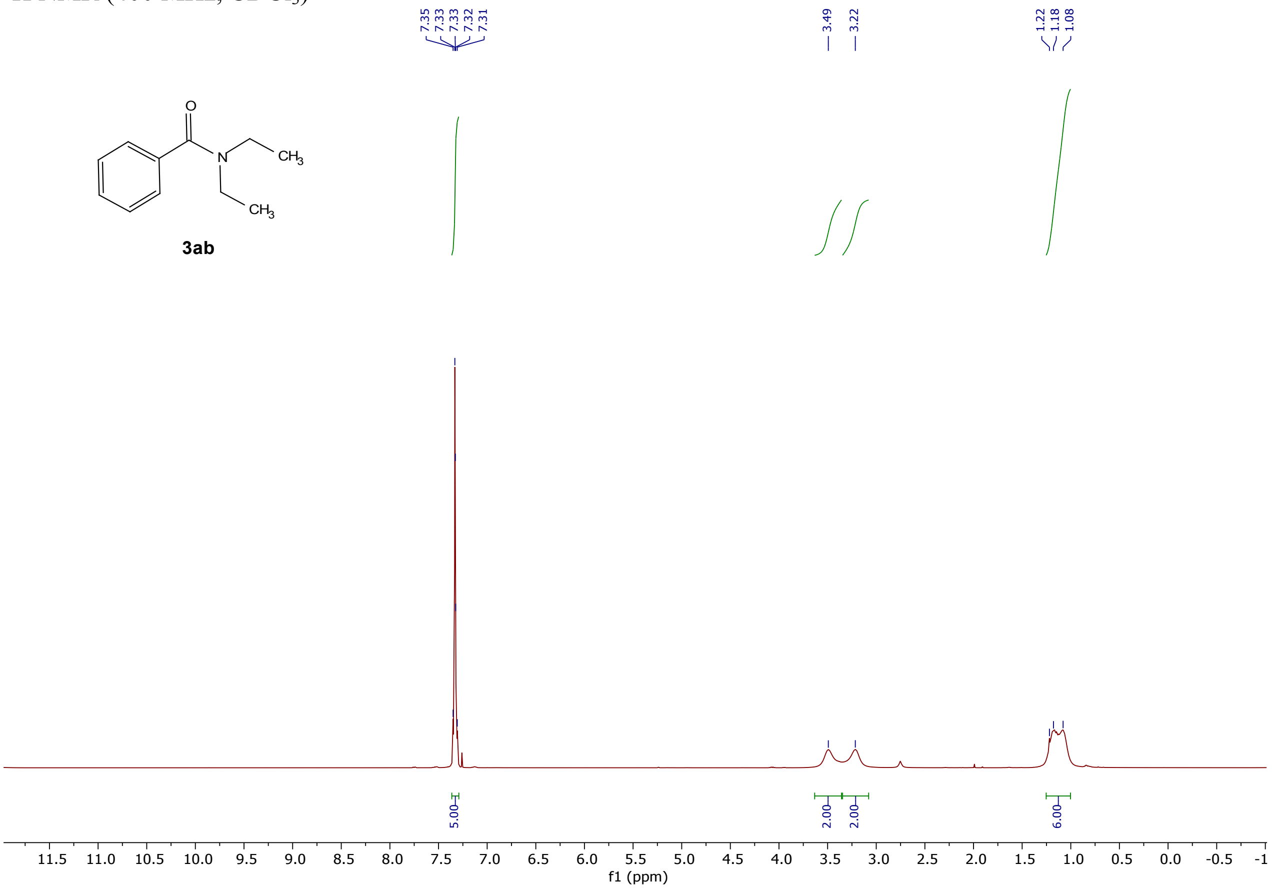

^{13}C{^{1}H} NMR (100 MHz, $CDCl_3$)

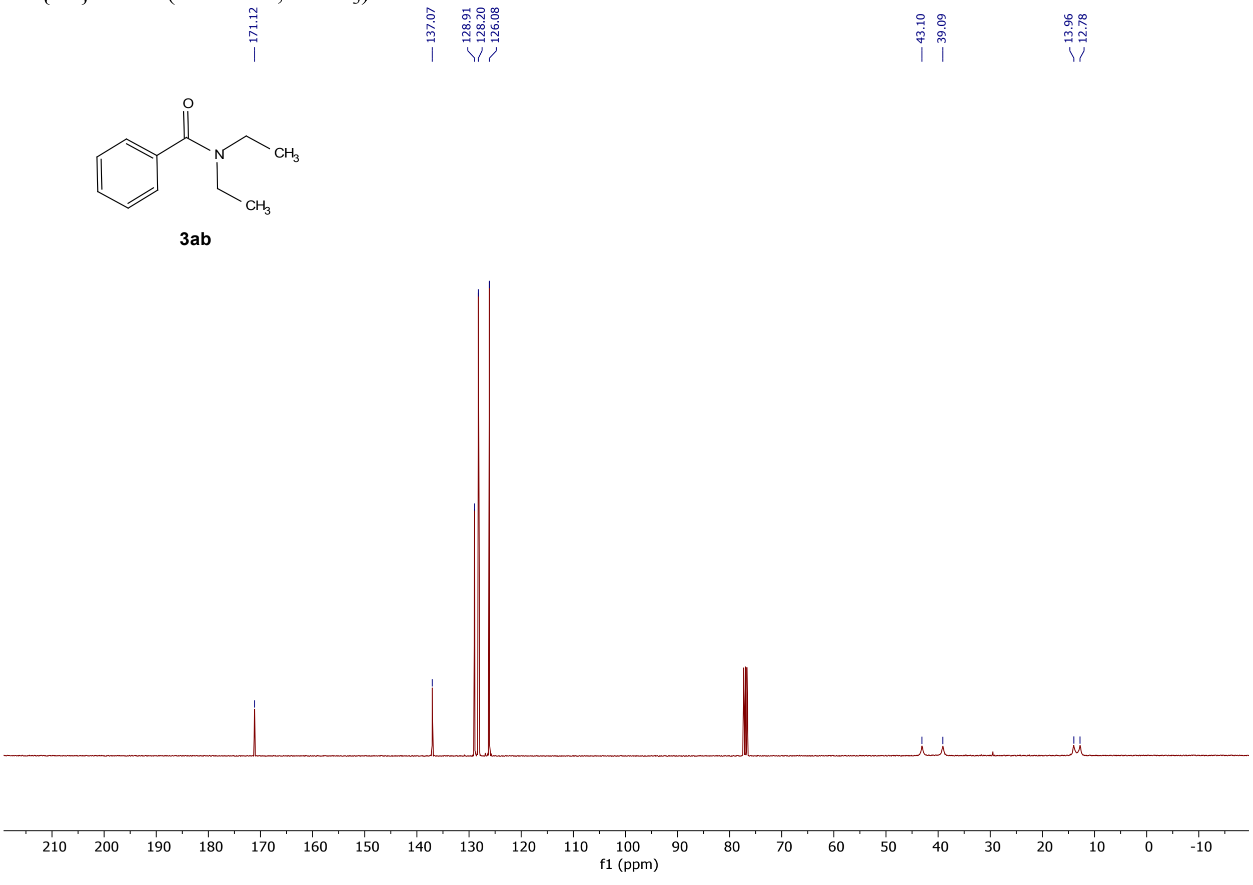

4-methoxy-*N,N*-dimethylbenzamide (3ac):

^{1}H NMR (400 MHz, $CDCl_3$)

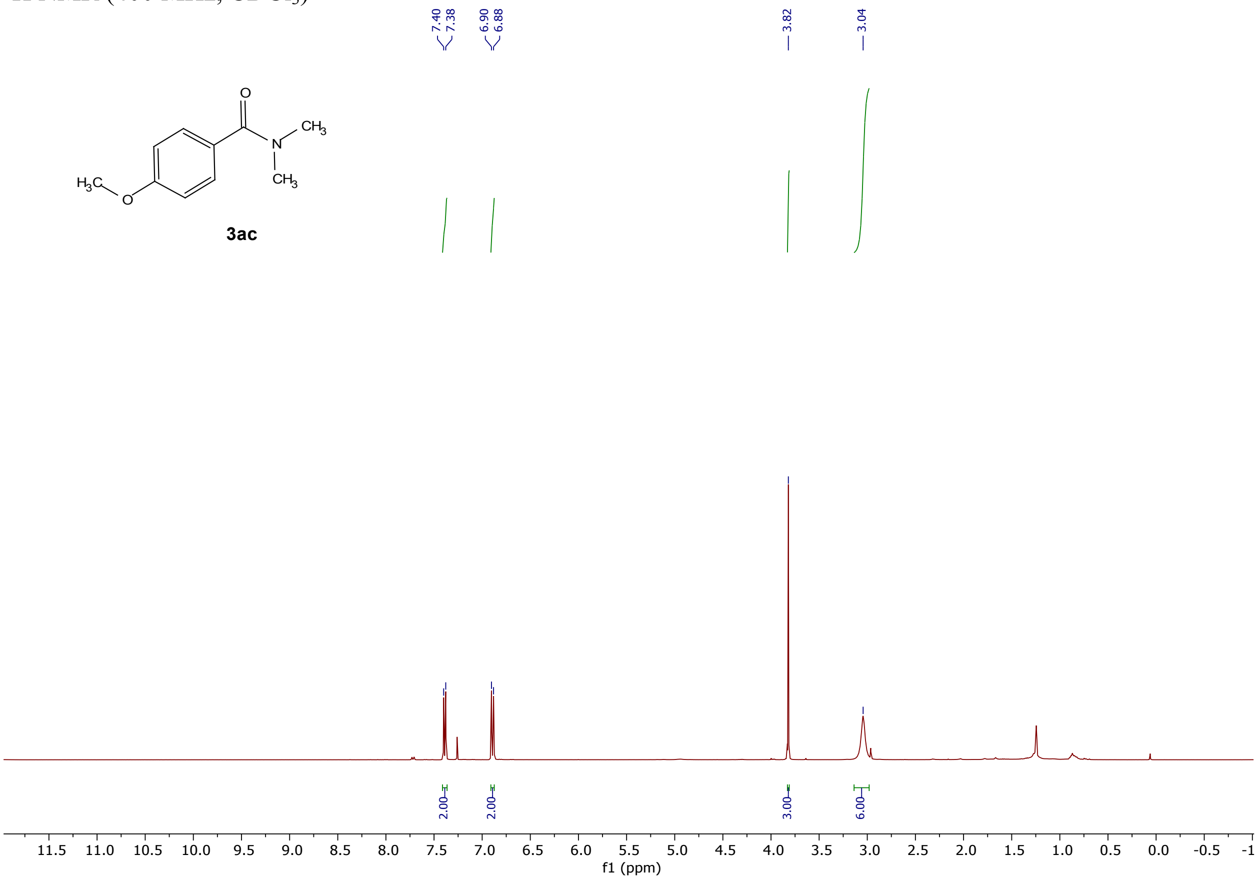

^{13}C{^{1}H} NMR (100 MHz, $CDCl_3$)

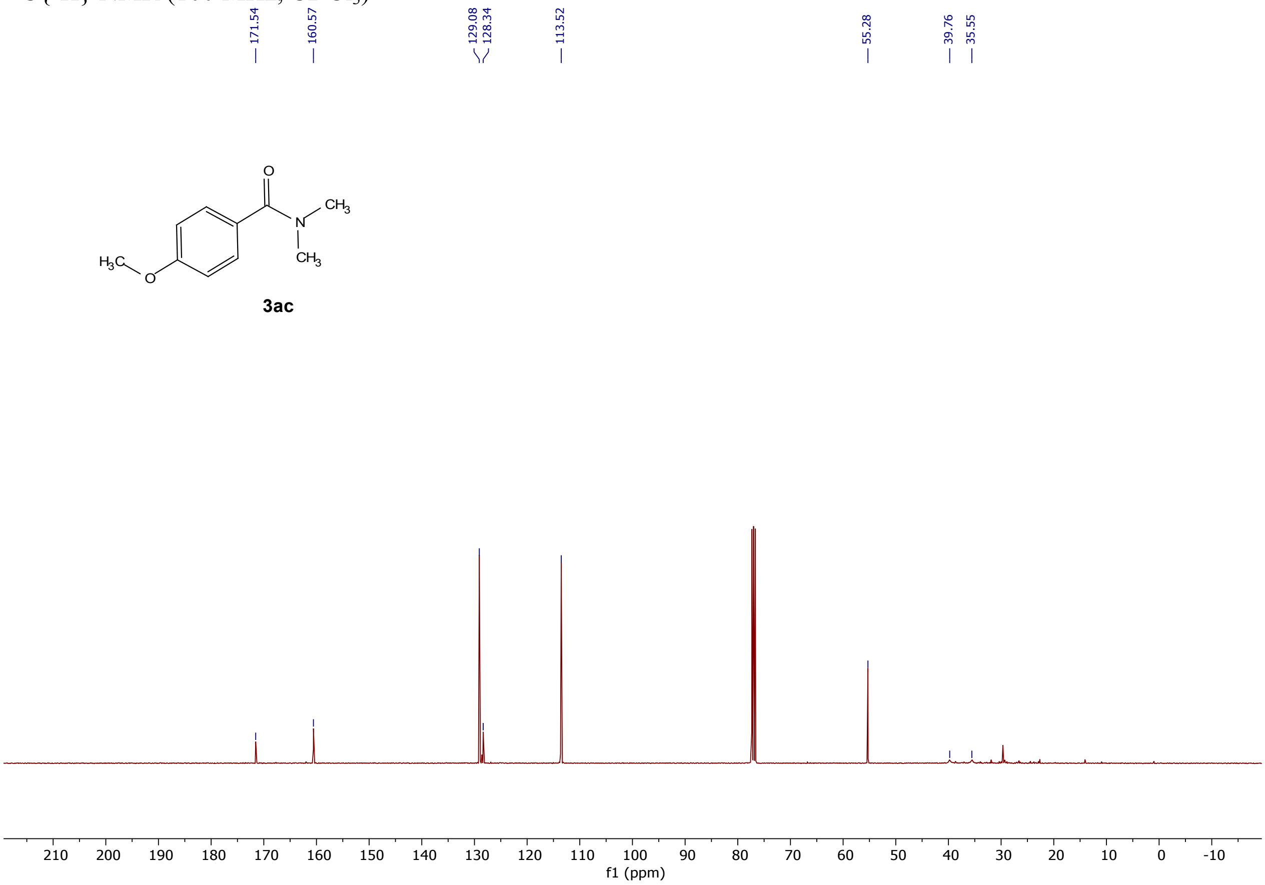

2-methoxy-*N,N*-dimethylbenzamide (3ad)

^{1}H NMR (400 MHz, $CDCl_3$)

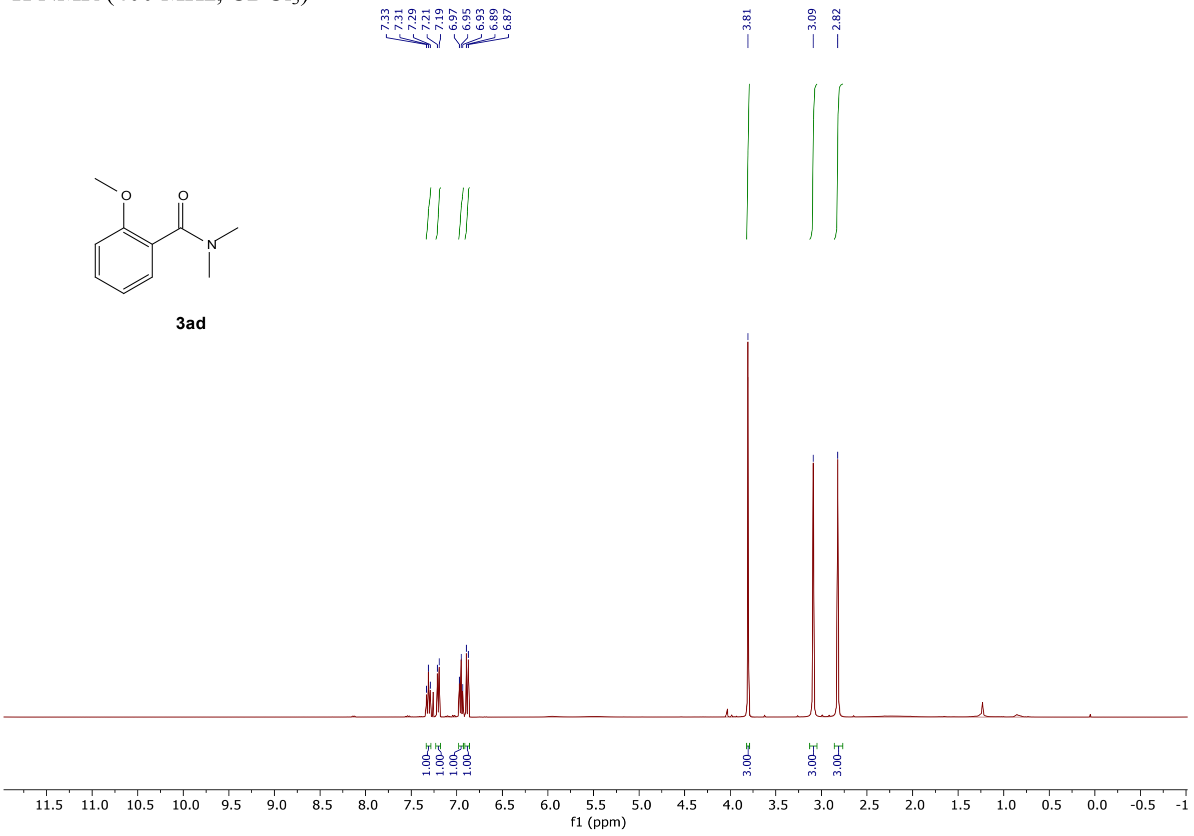

$^{13}C\{^{1}H\}$ NMR (100 MHz, $CDCl_3$)

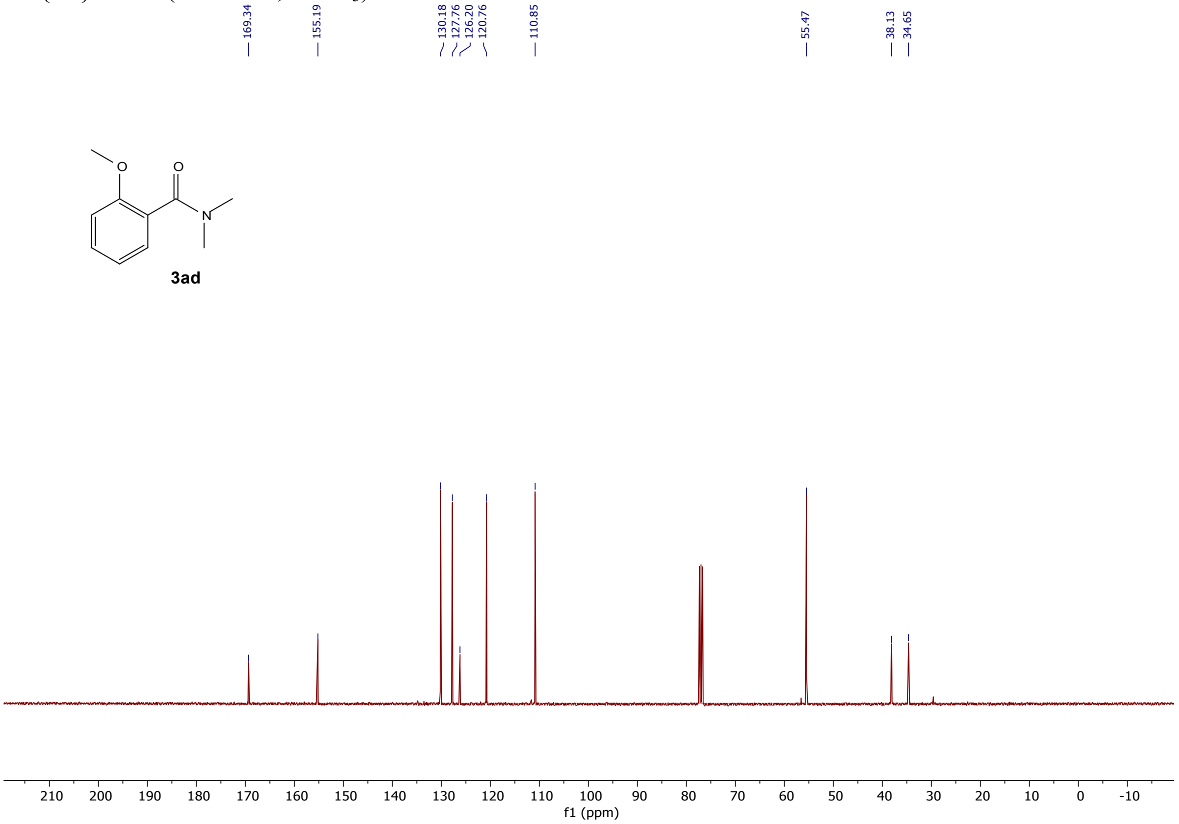

***N*,*N*,2-trimethylbenzamide (3ae)**

^{1}H NMR (400 MHz, $CDCl_3$)

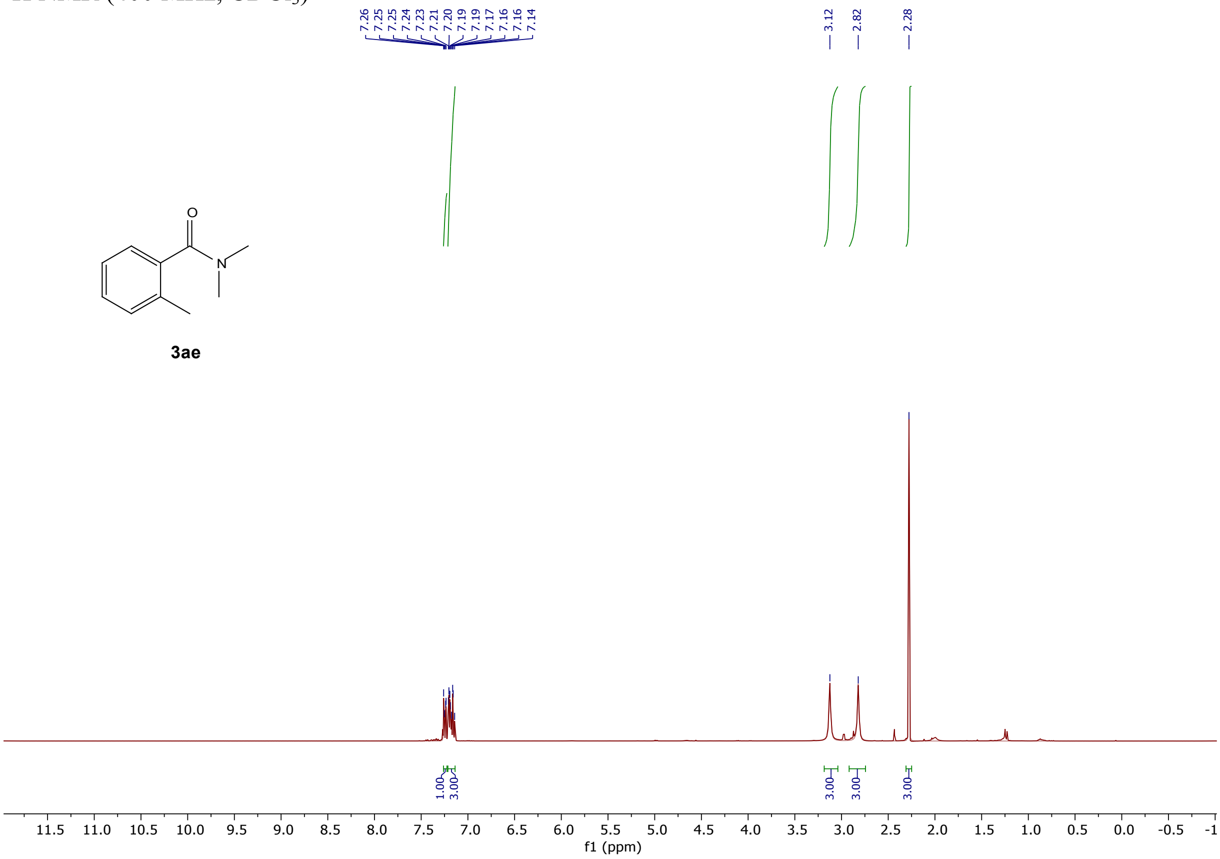

3ae

$^{13}C\{^{1}H\}$ NMR (100 MHz, $CDCl_3$)

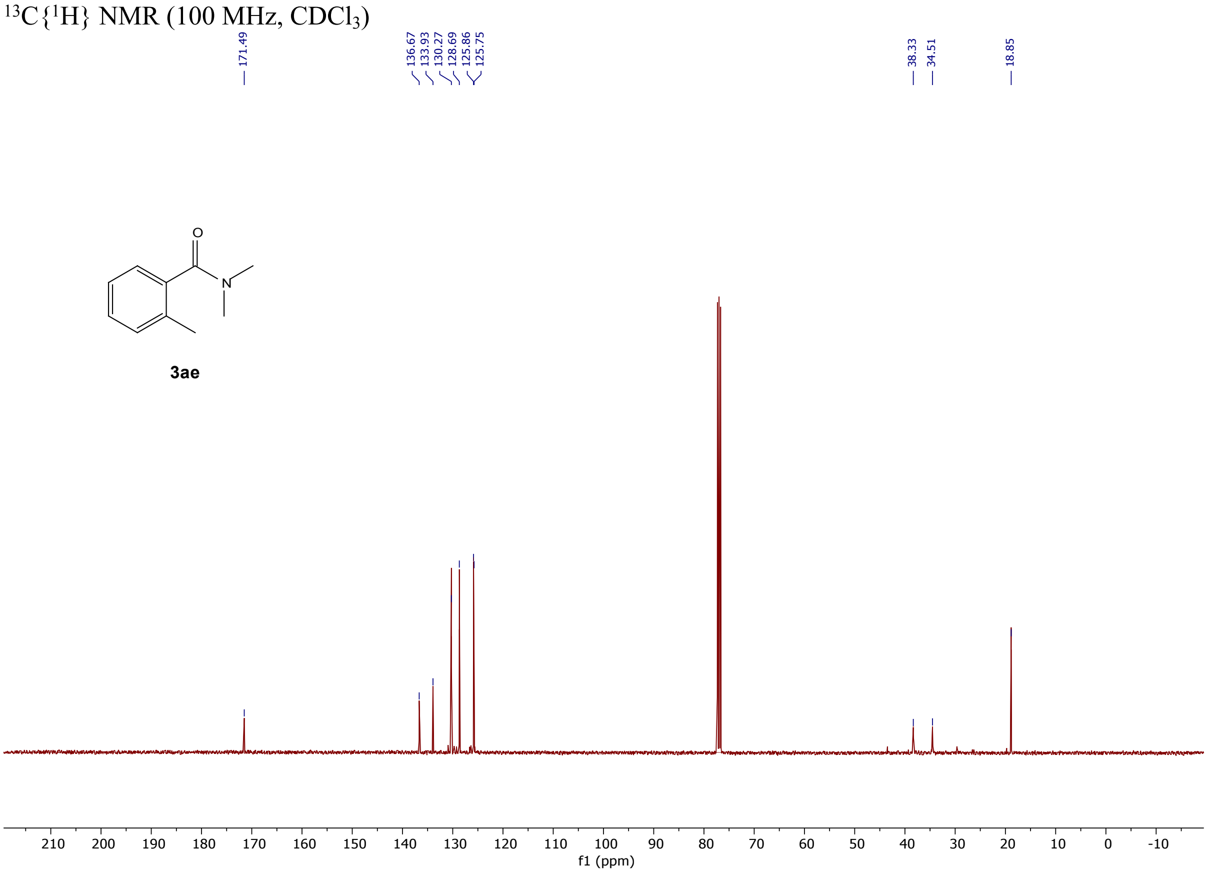

3ae

***N,N*-dimethyl-[1,1'-biphenyl]-4-carboxamide (3af):**

^{1}H NMR (400 MHz, $CDCl_3$)

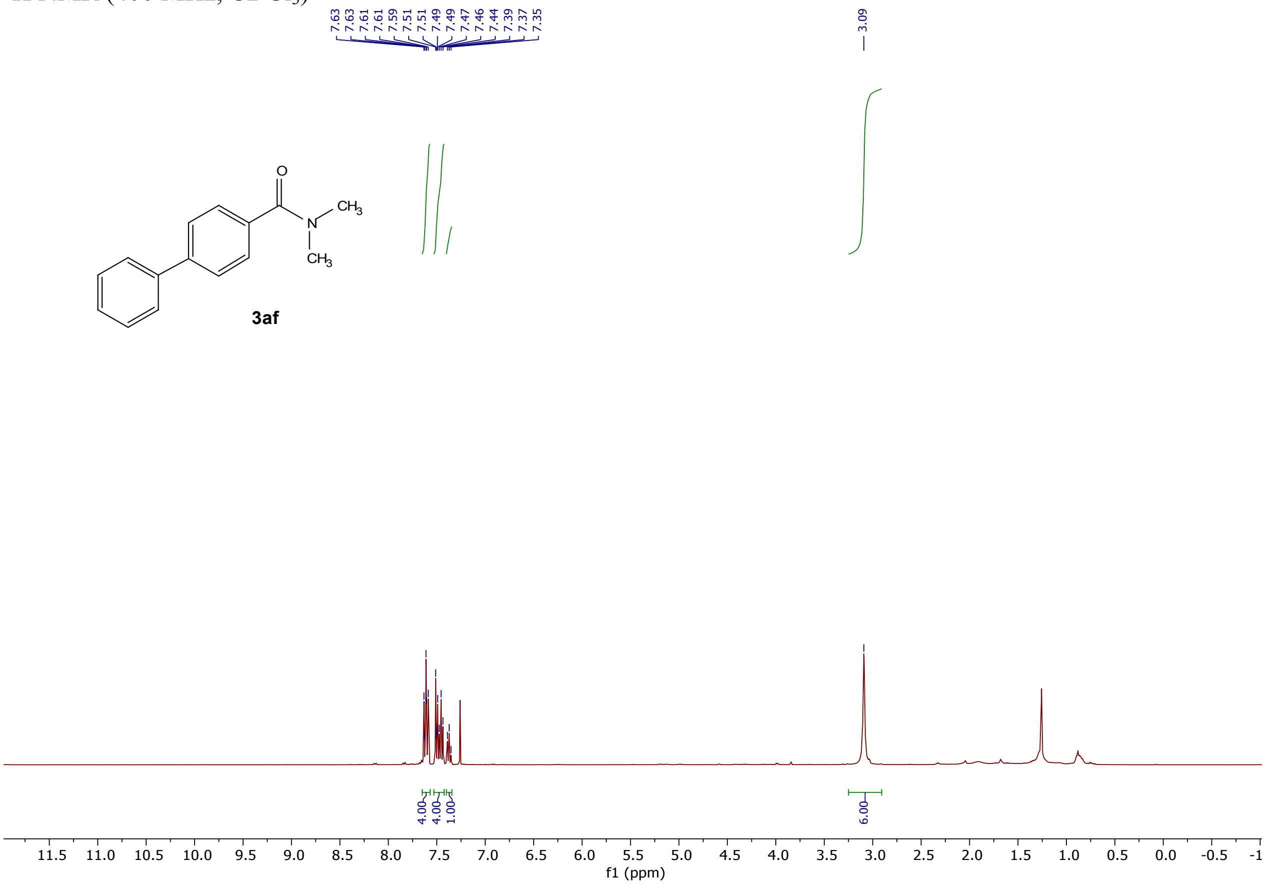

^{13}C{^{1}H} NMR (100 MHz, $CDCl_3$)

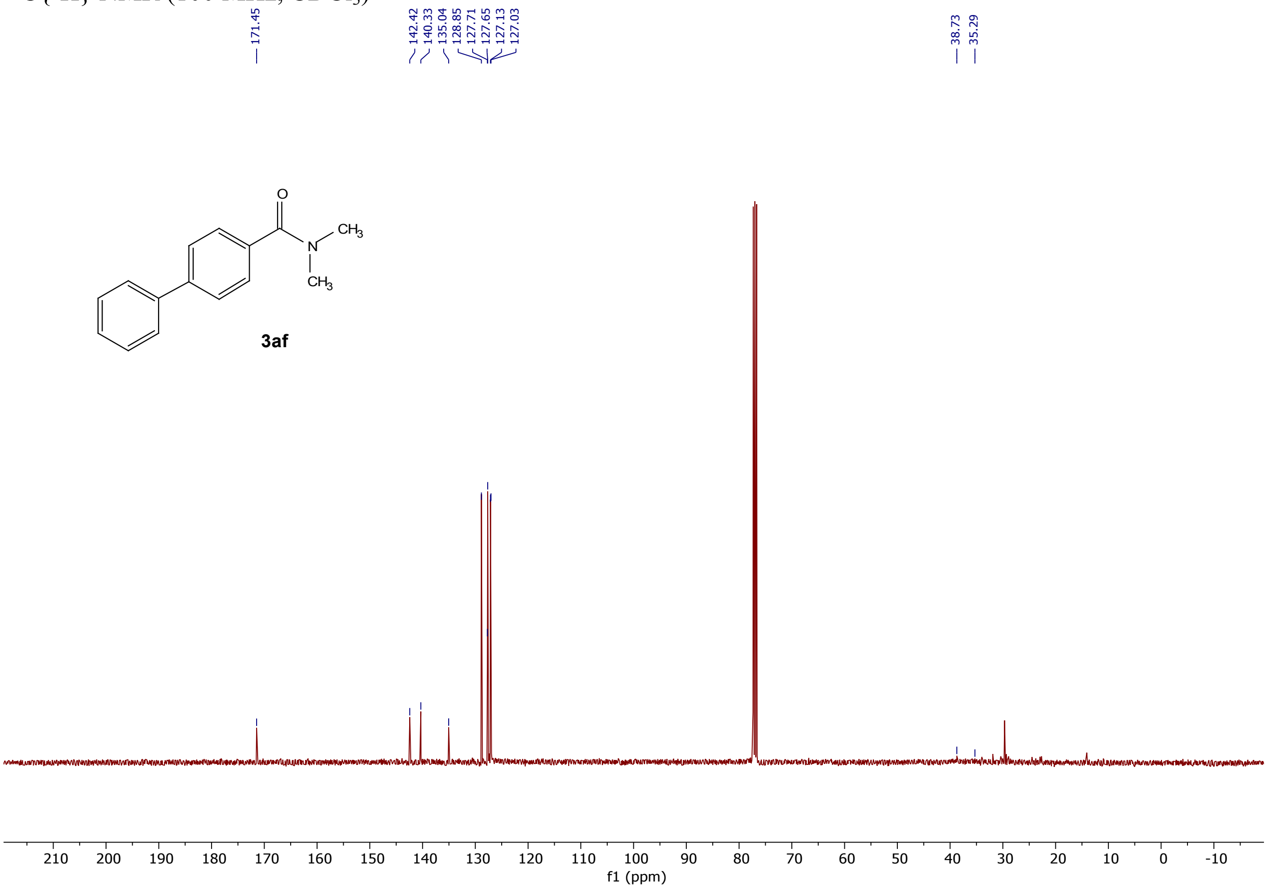

***N*,*N*,3,5-tetramethylbenzamide (3ag)**

^{1}H NMR (400 MHz, $CDCl_3$)

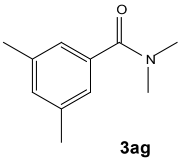

3ag

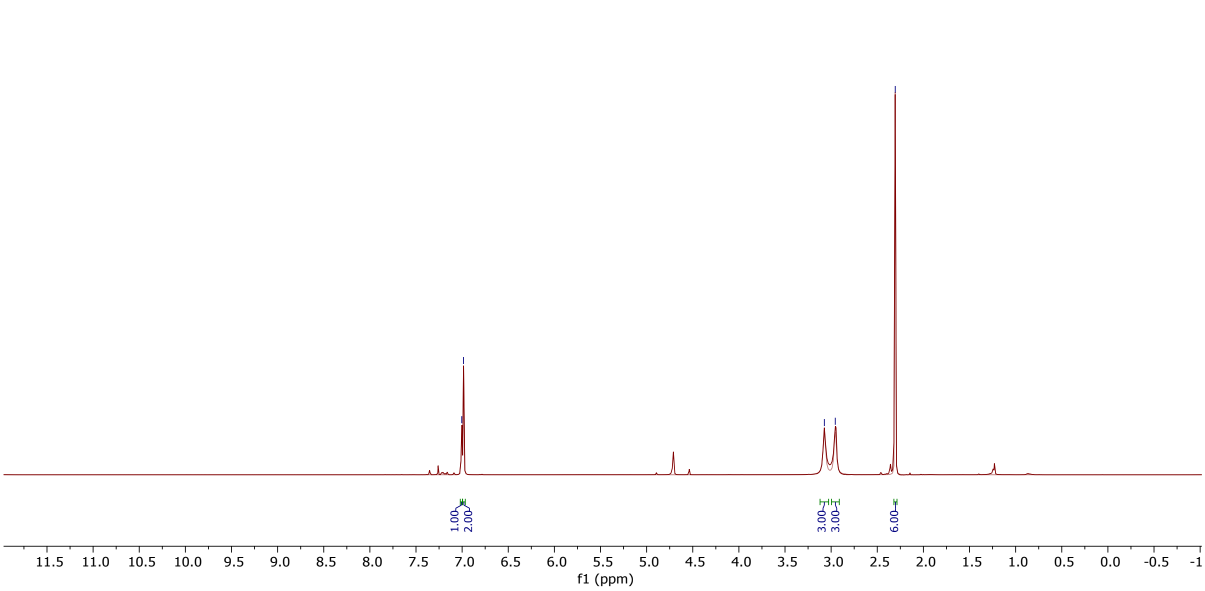

^{13}C{^{1}H} NMR (100 MHz, $CDCl_3$)

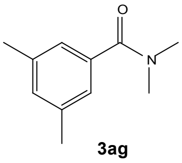

3ag

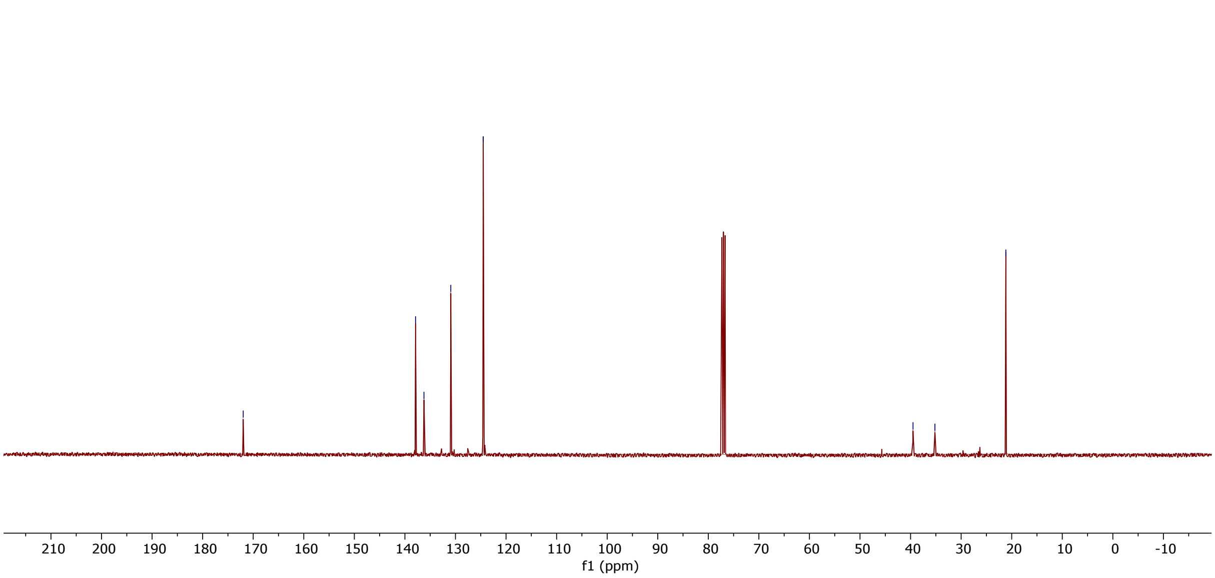

4-chloro-*N,N*-dimethylbenzamide (3ah):

^{1}H NMR (400 MHz, $CDCl_3$)

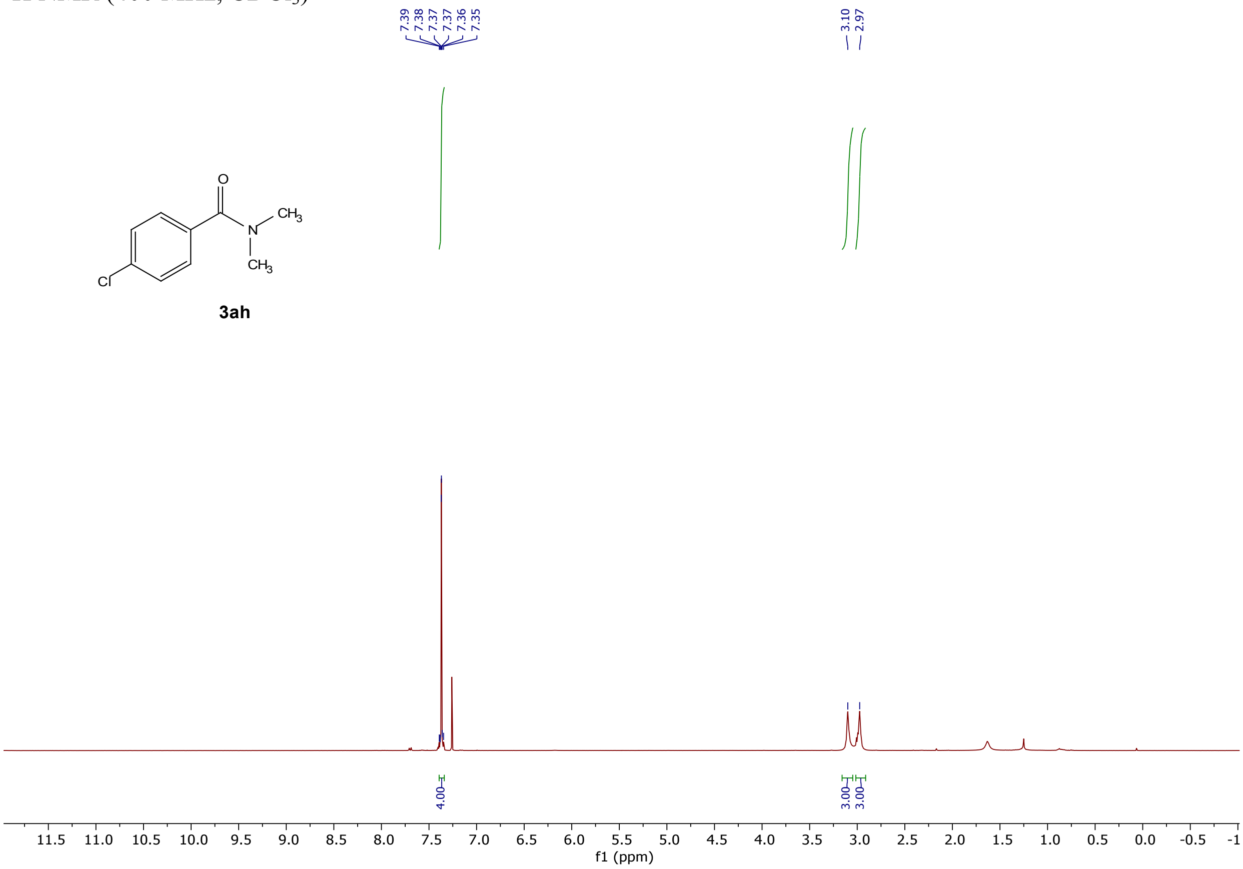

$^{13}C\{^{1}H\}$ NMR (100 MHz, $CDCl_3$)

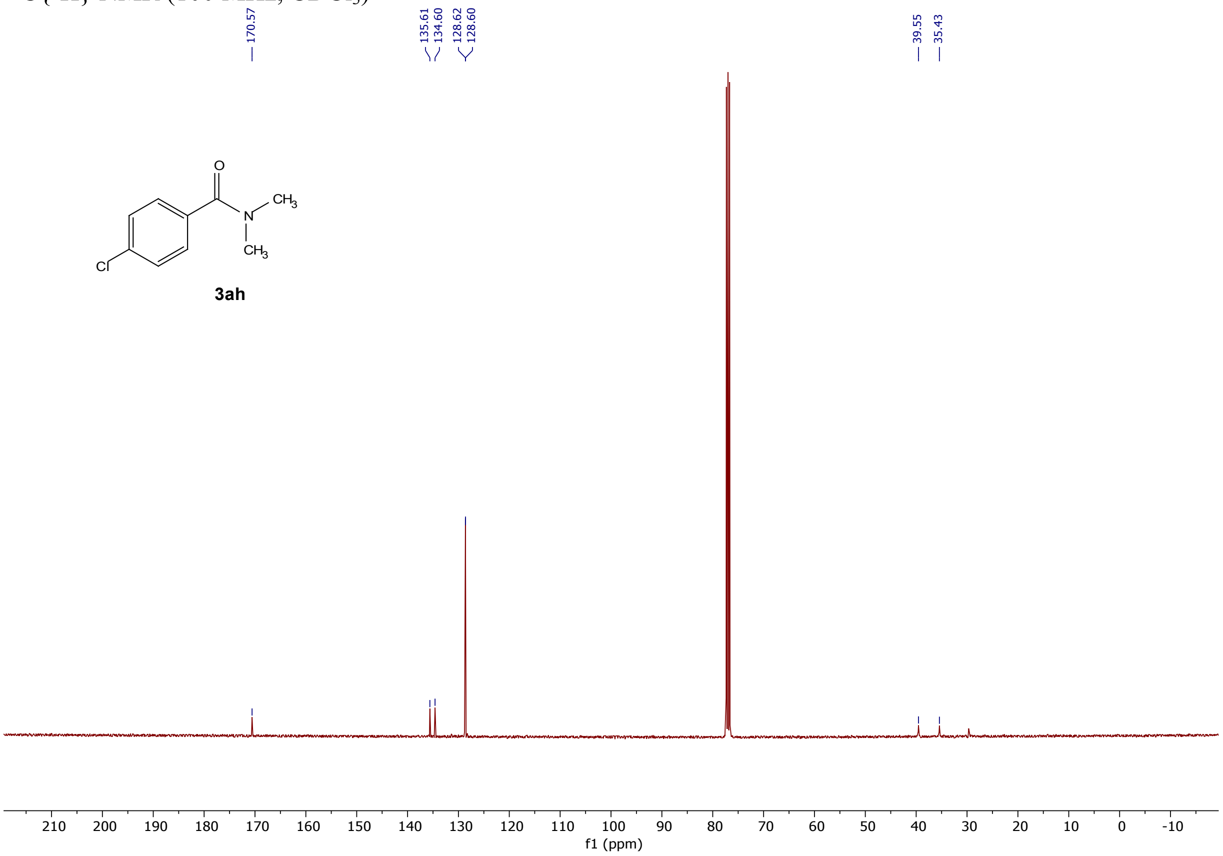

2-chloro-*N*,*N*-dimethylbenzamide (3ai):

^{1}H NMR (400 MHz, $CDCl_3$)

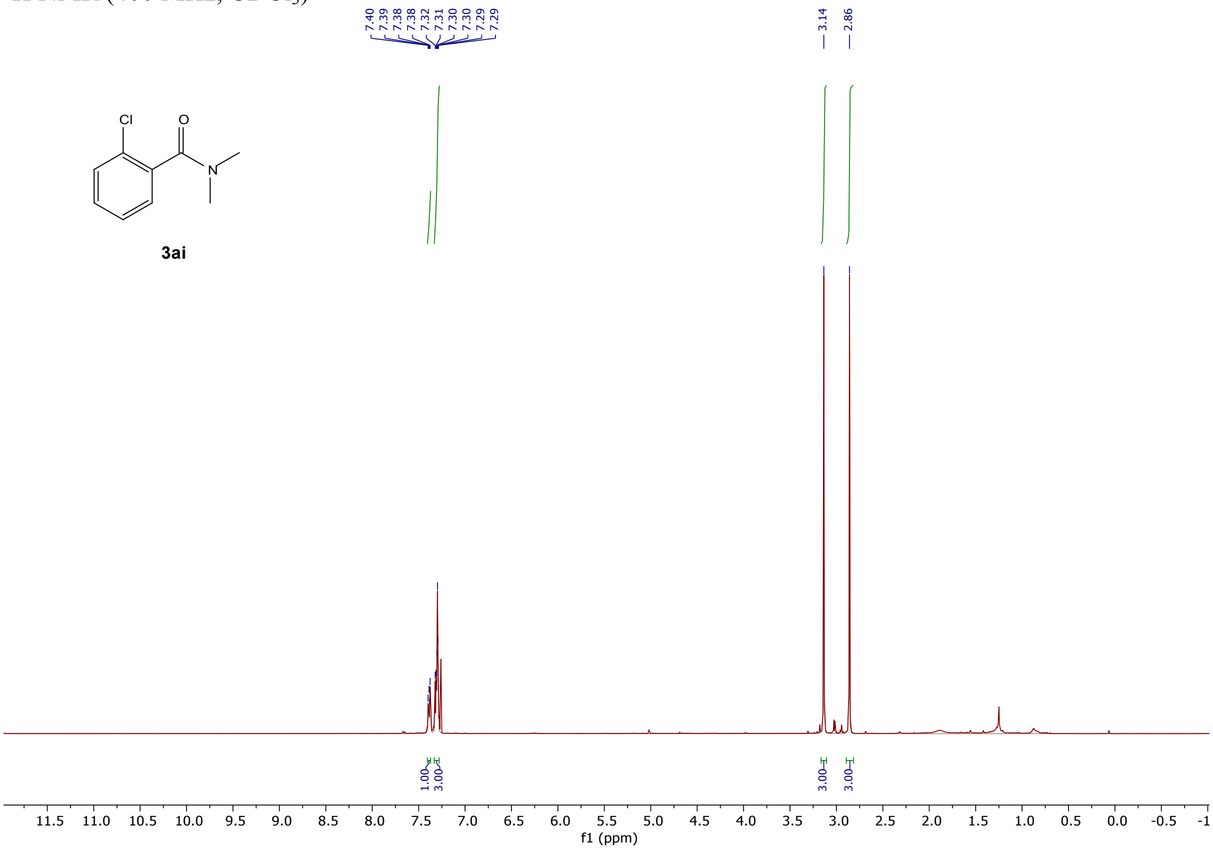

$^{13}C\{^{1}H\}$ NMR (100 MHz, $CDCl_3$)

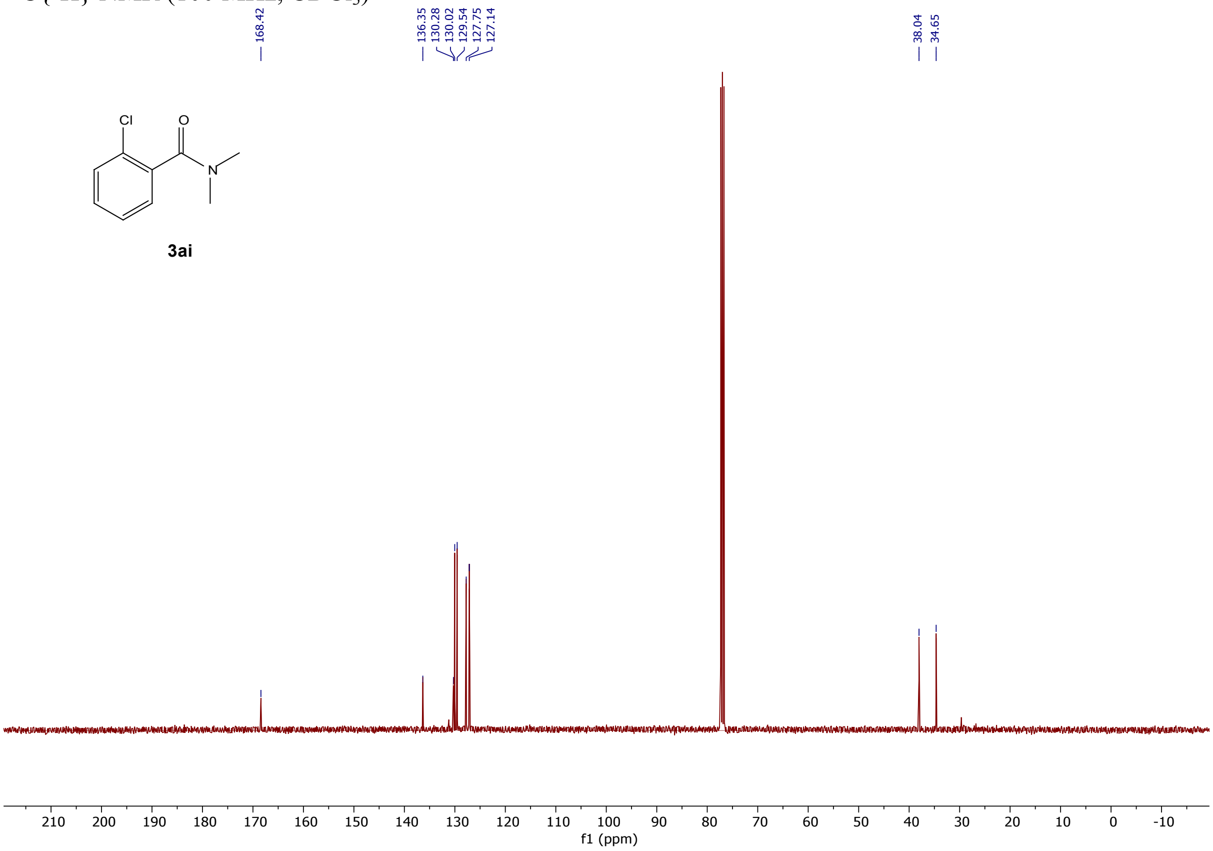

4-bromo-*N*,*N*-dimethylbenzamide (3aj):

^{1}H NMR (400 MHz, $CDCl_3$)

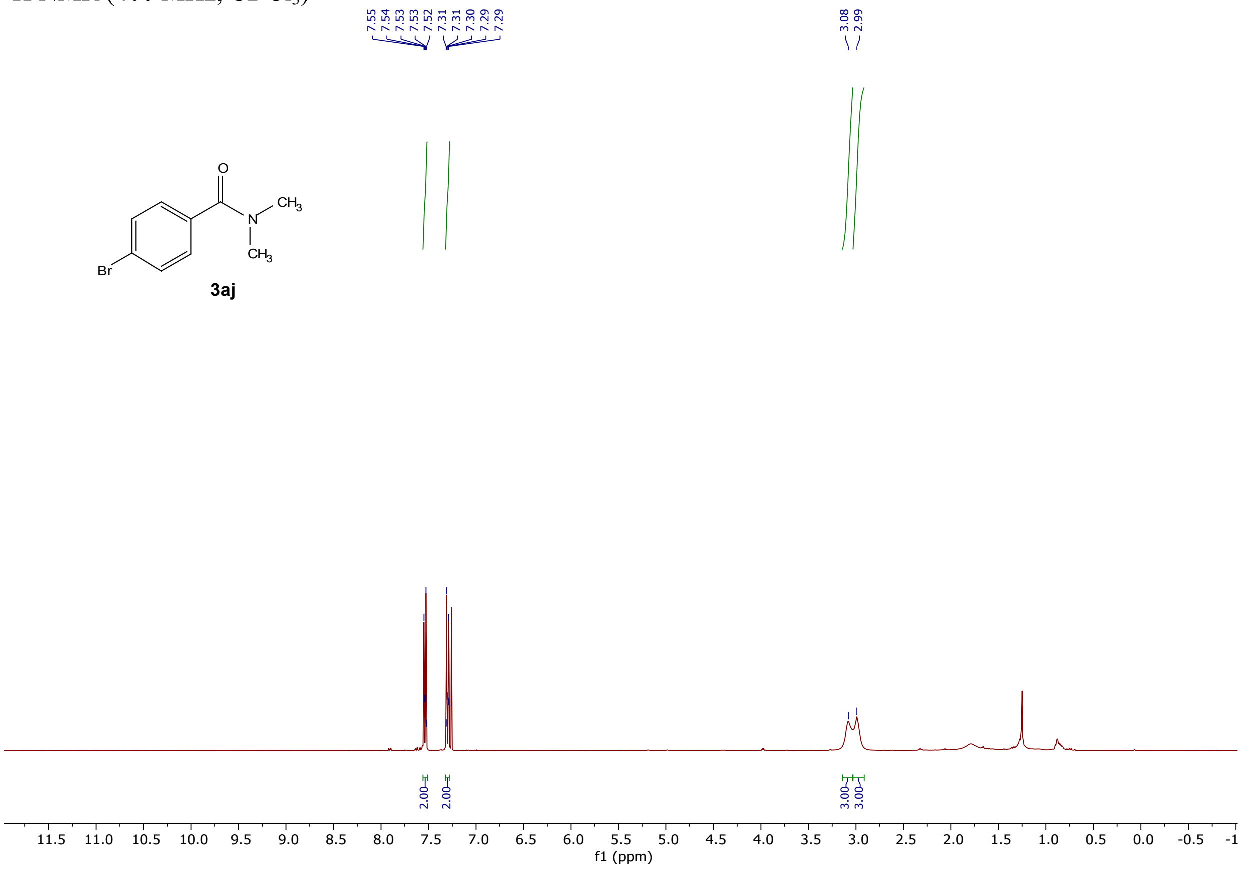

^{13}C{^{1}H} NMR (100 MHz, $CDCl_3$)

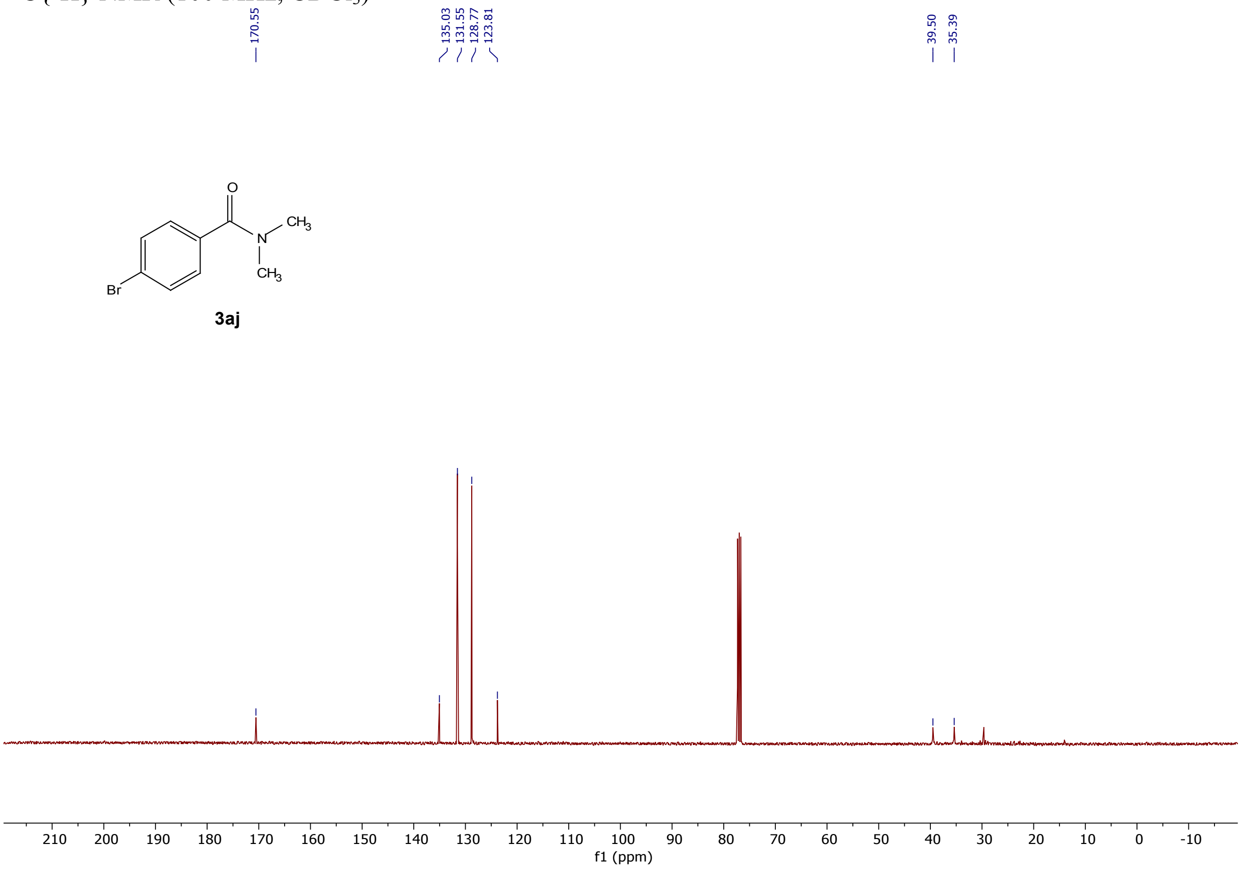

***N,N*-dimethyl-4-(trifluoromethyl)benzamide (3ak):**

^{1}H NMR (400 MHz, $CDCl_3$)

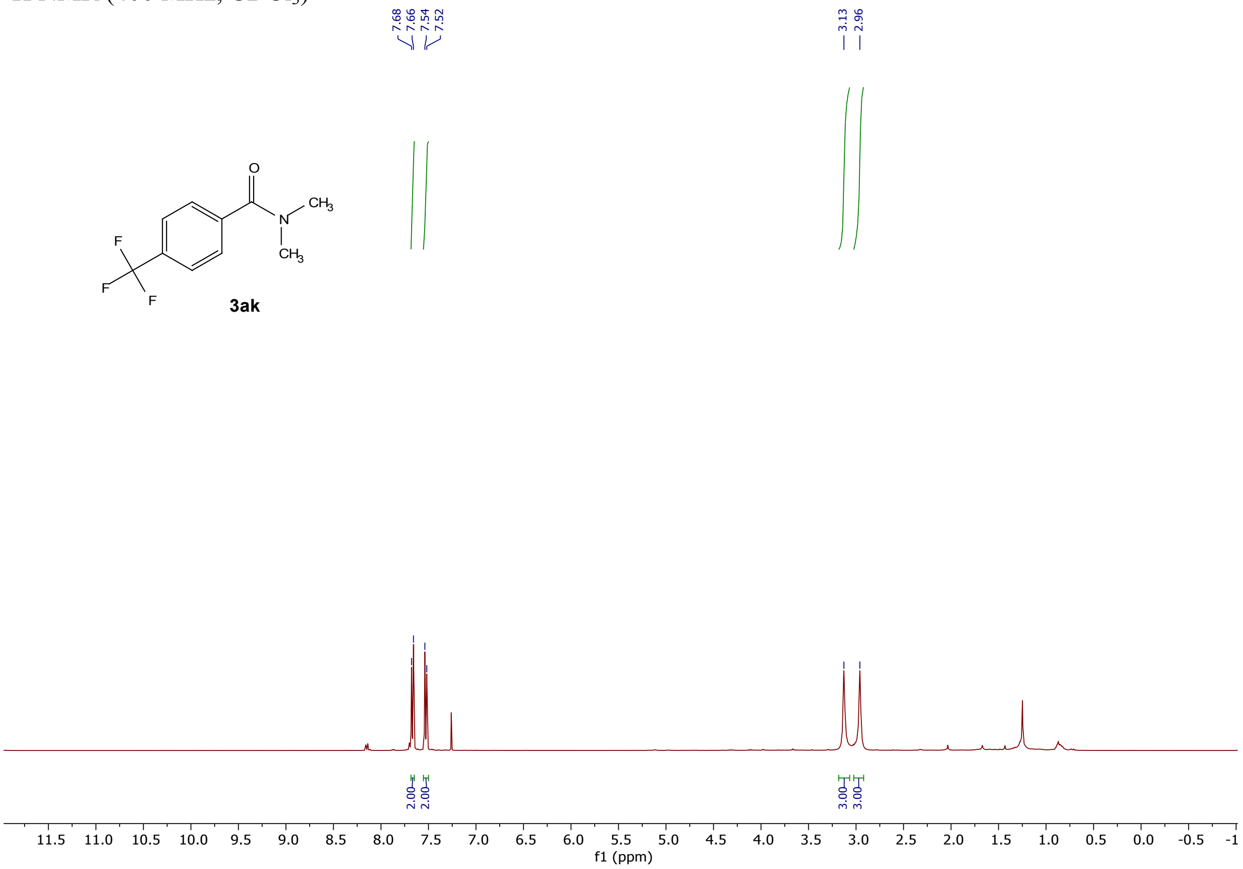

$^{13}C\{^{1}H\}$ NMR (100 MHz, $CDCl_3$)

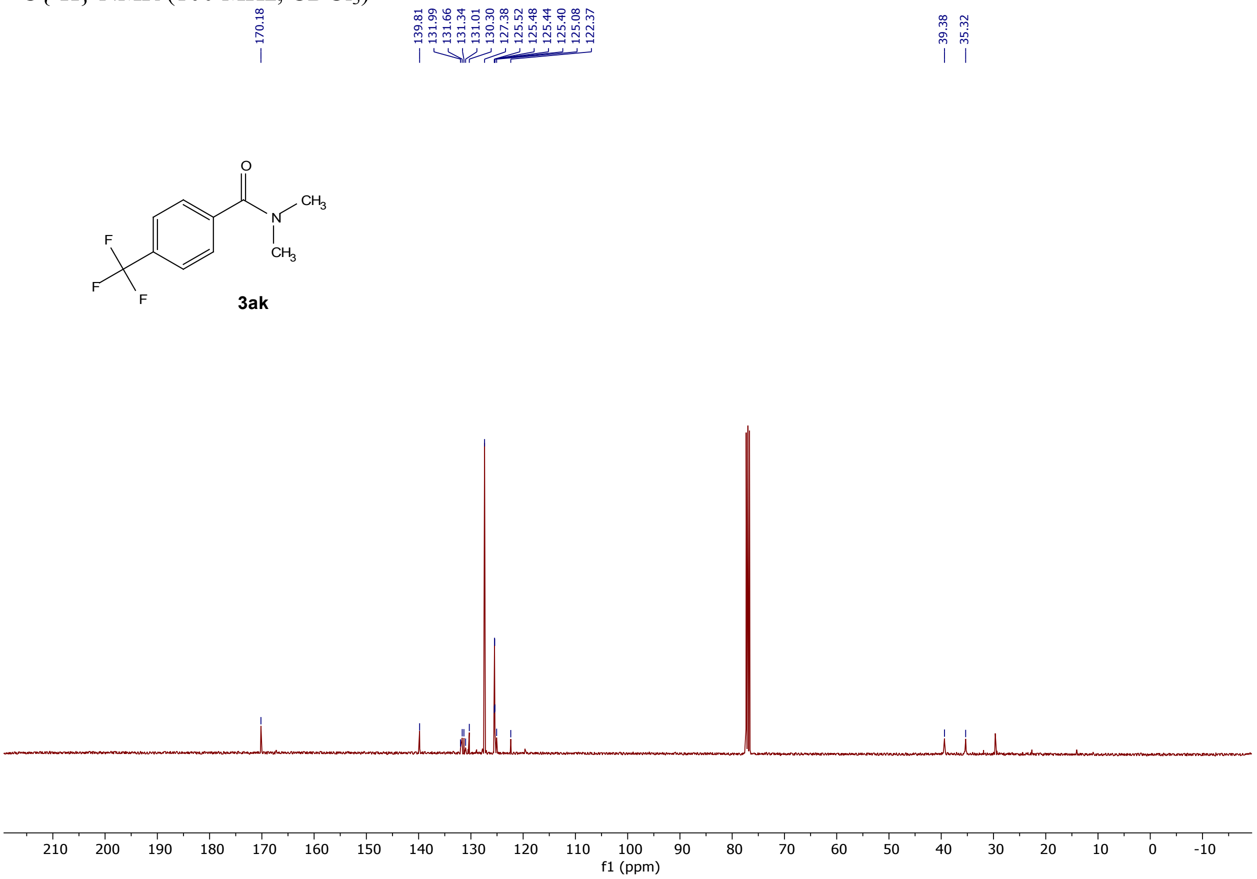

Morpholino(phenyl)methanone (3al):

^{1}H NMR (400 MHz, $CDCl_3$)

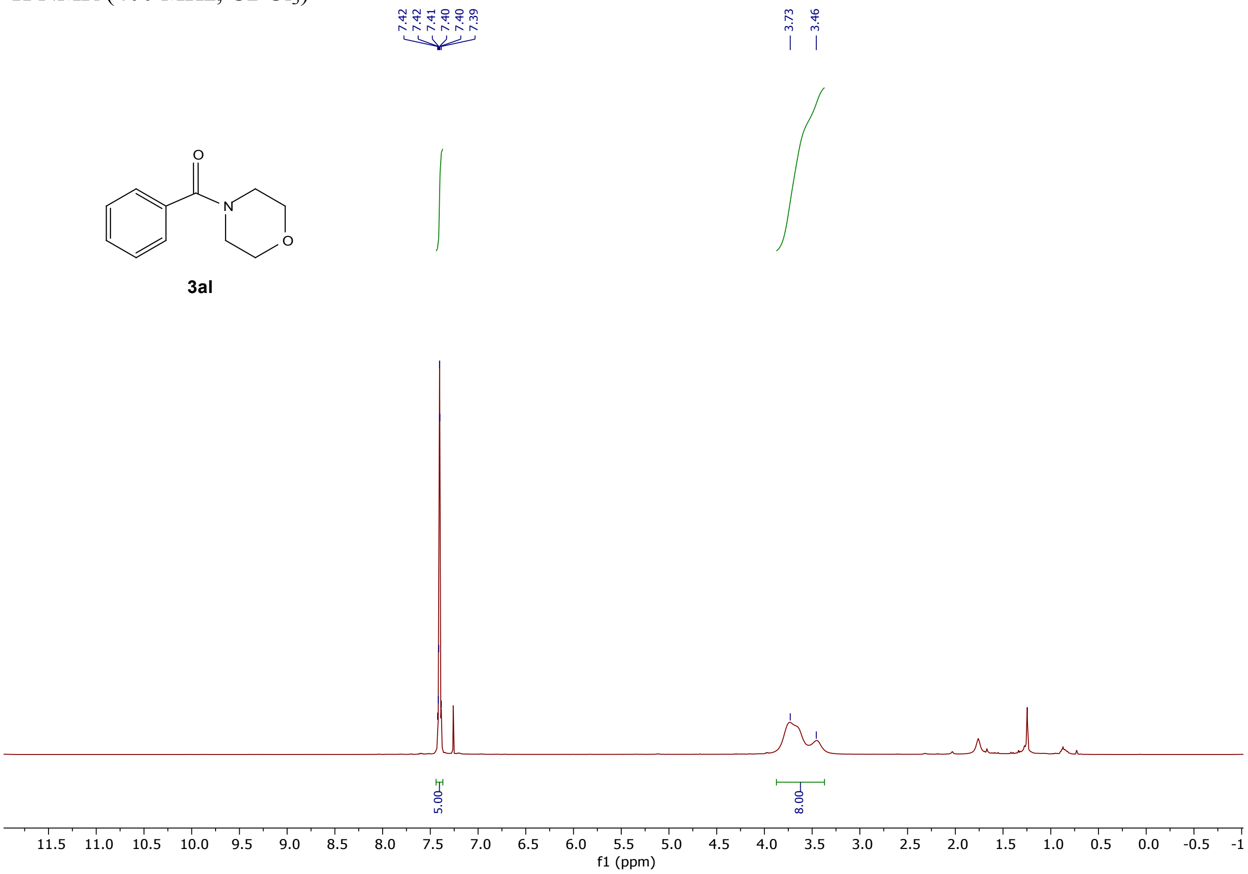

$^{13}C\{^{1}H\}$ NMR (100 MHz, $CDCl_3$)

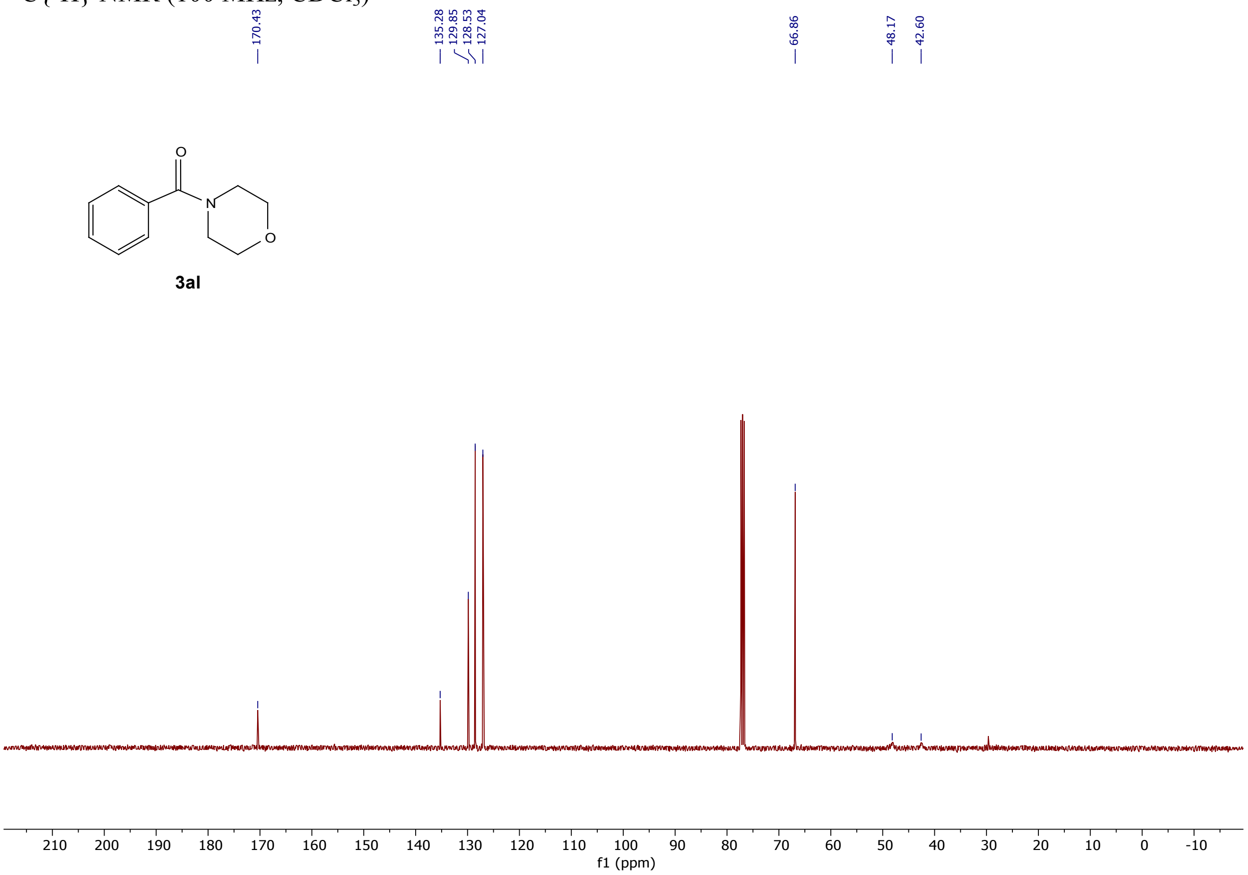

(4-methoxyphenyl)(morpholino)methanone (3am):

^{1}H NMR (400 MHz, $CDCl_3$)

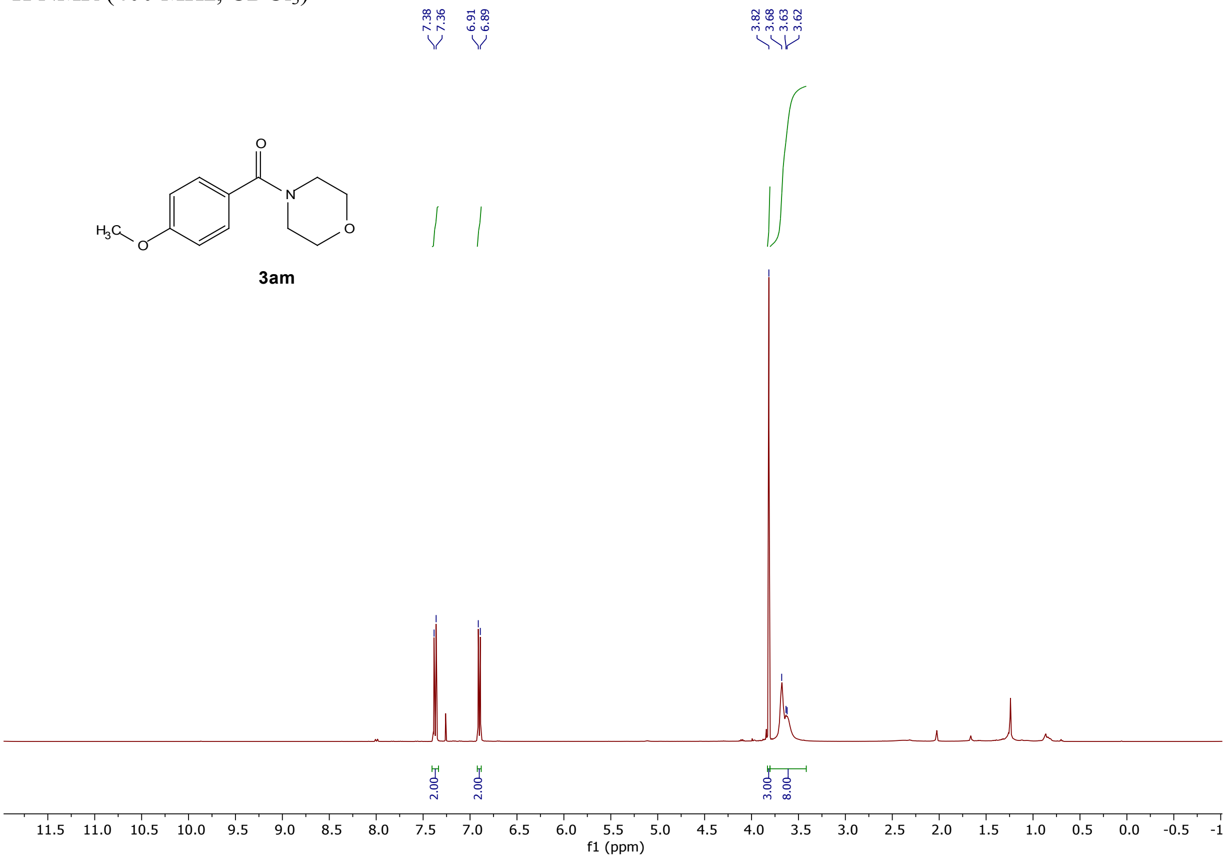

$^{13}C\{^{1}H\}$ NMR (100 MHz, $CDCl_3$)

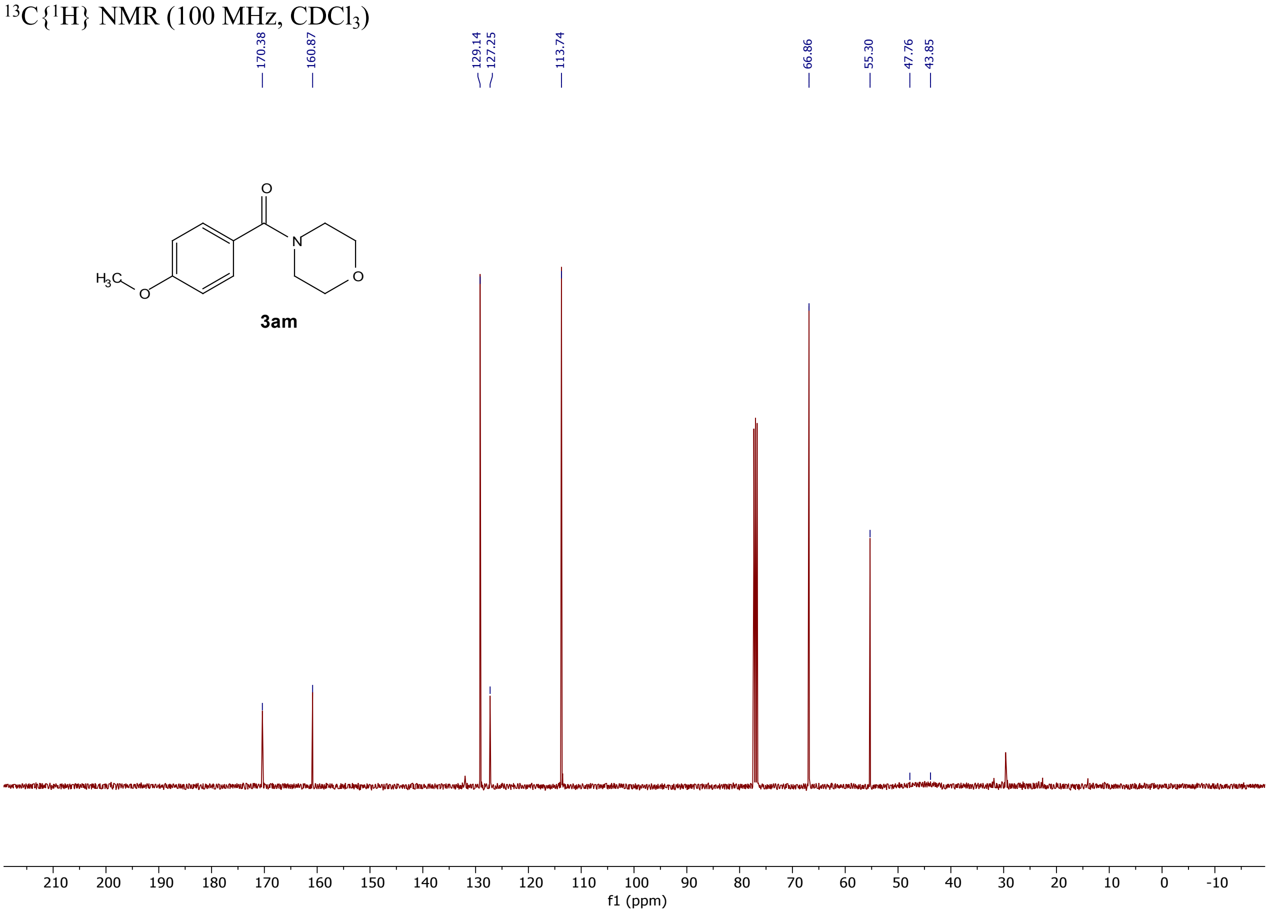

[1,1'-biphenyl]-4-yl(morpholino)methanone (3an):

^{1}H NMR (400 MHz, $CDCl_3$)

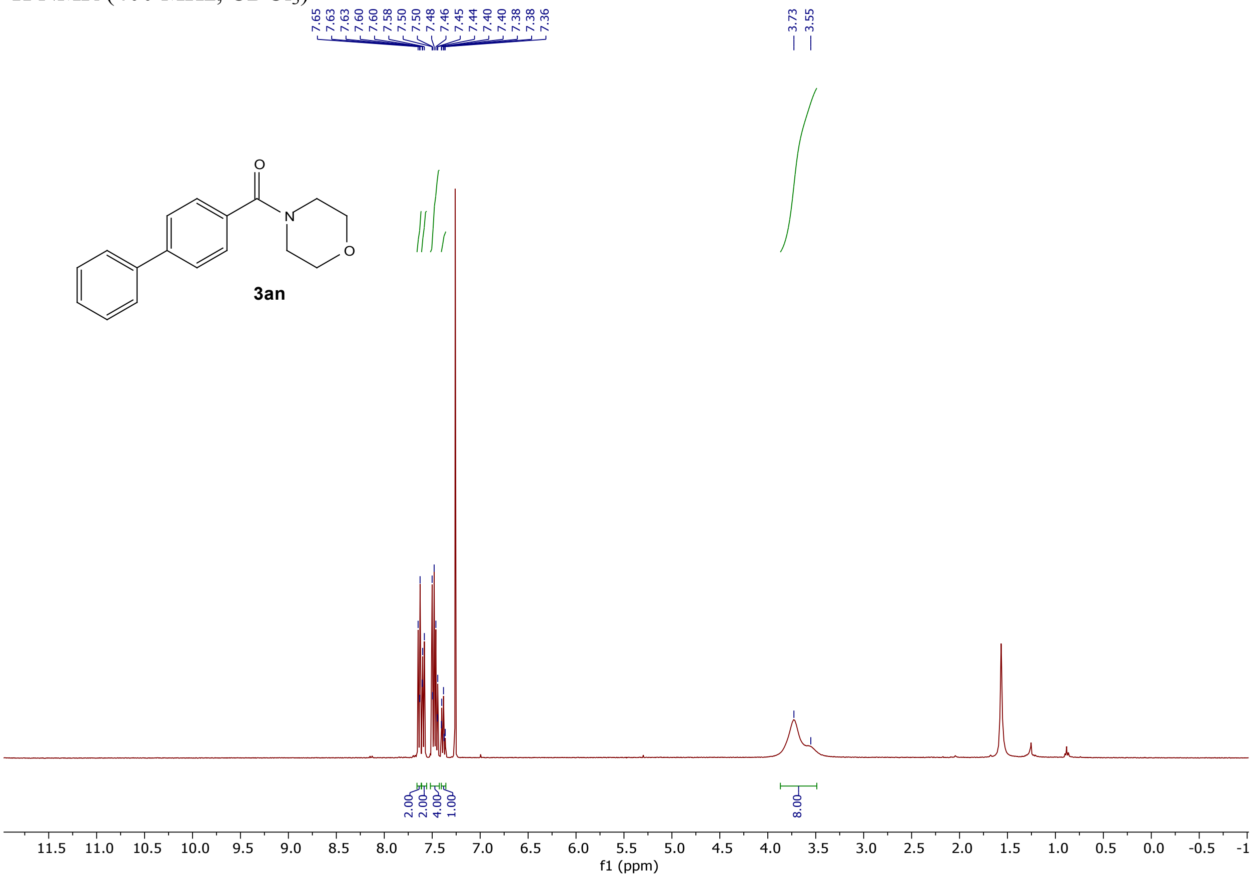

$^{13}C\{^{1}H\}$ NMR (100 MHz, $CDCl_3$)

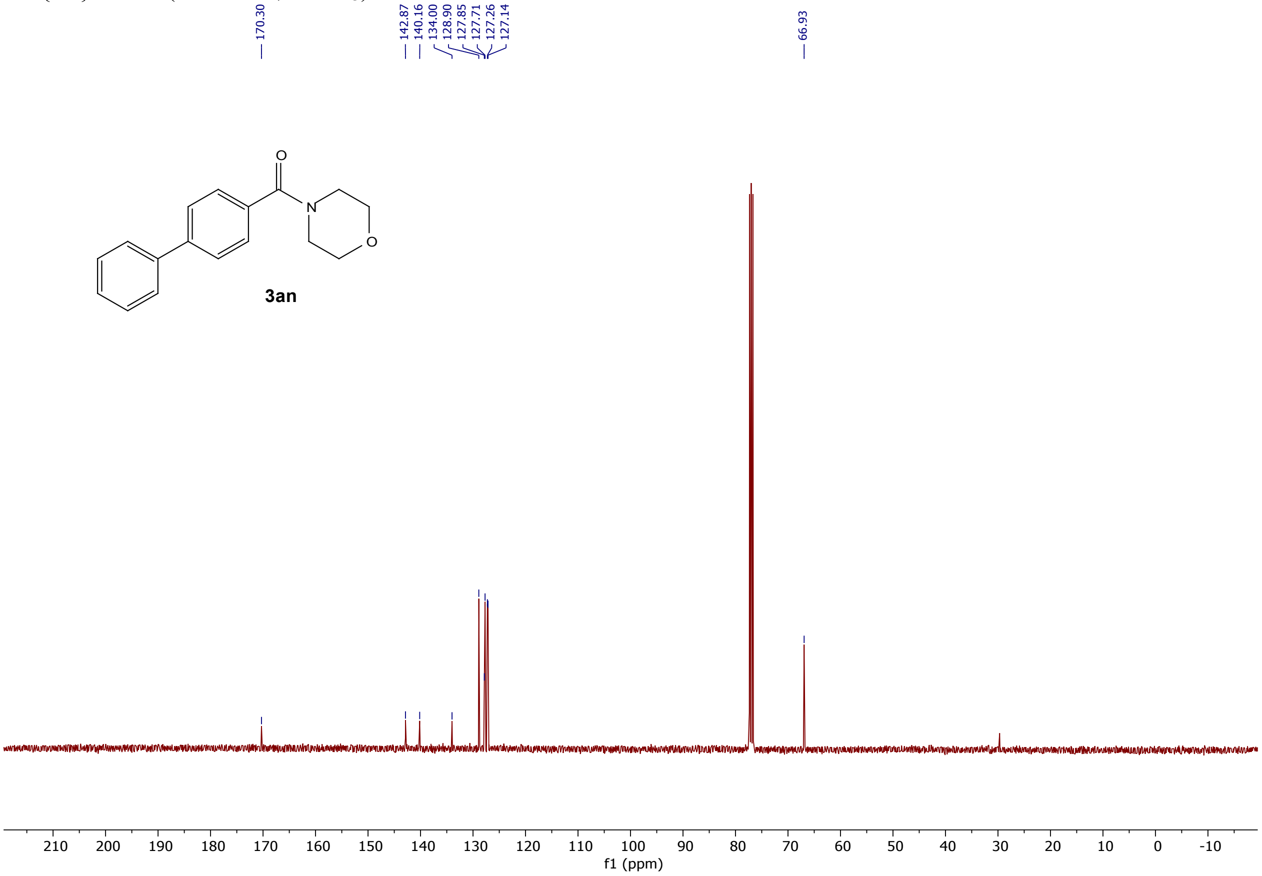

Morpholino(1*H*-pyrrol-2-yl)methanone (3ao):

^{1}H NMR (400 MHz, $CDCl_3$)

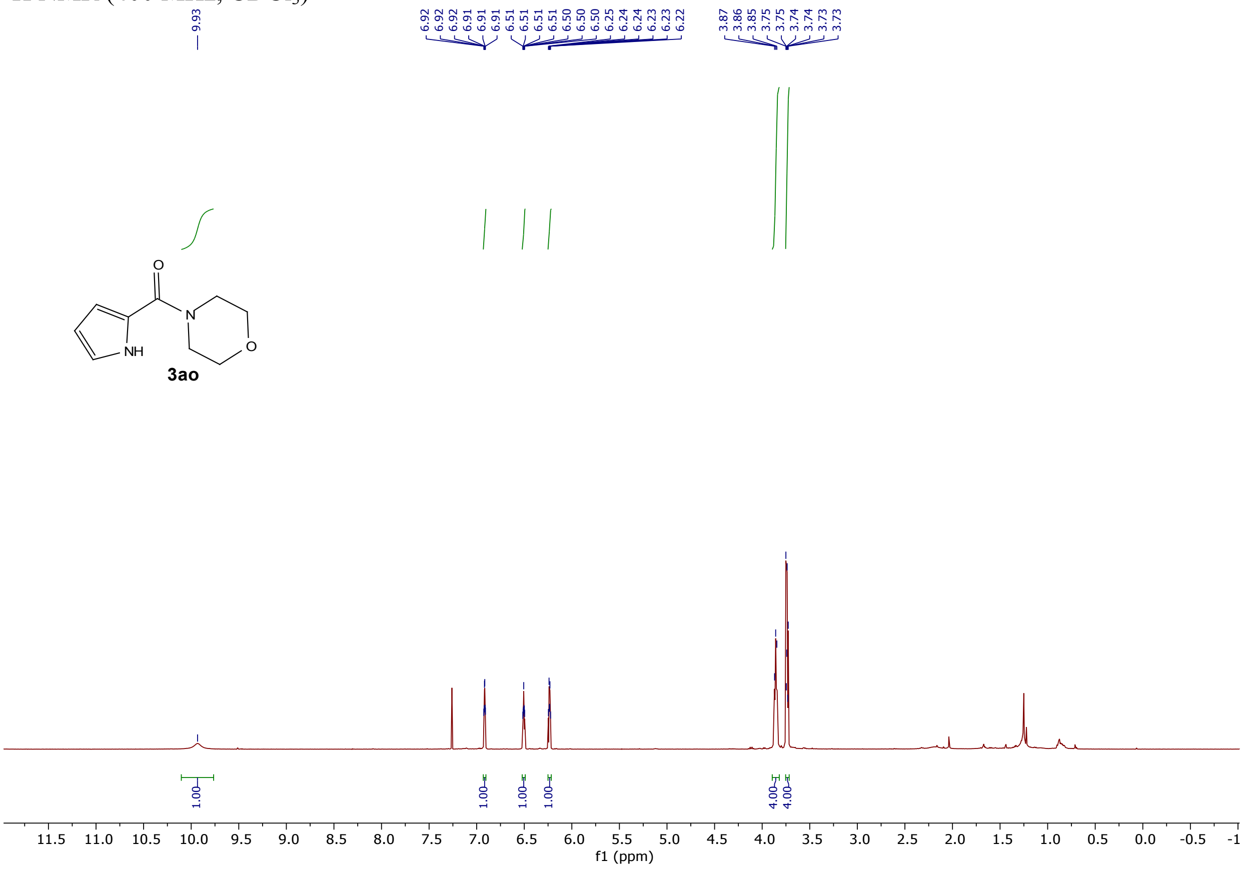

^{13}C{^{1}H} NMR (100 MHz, $CDCl_3$)

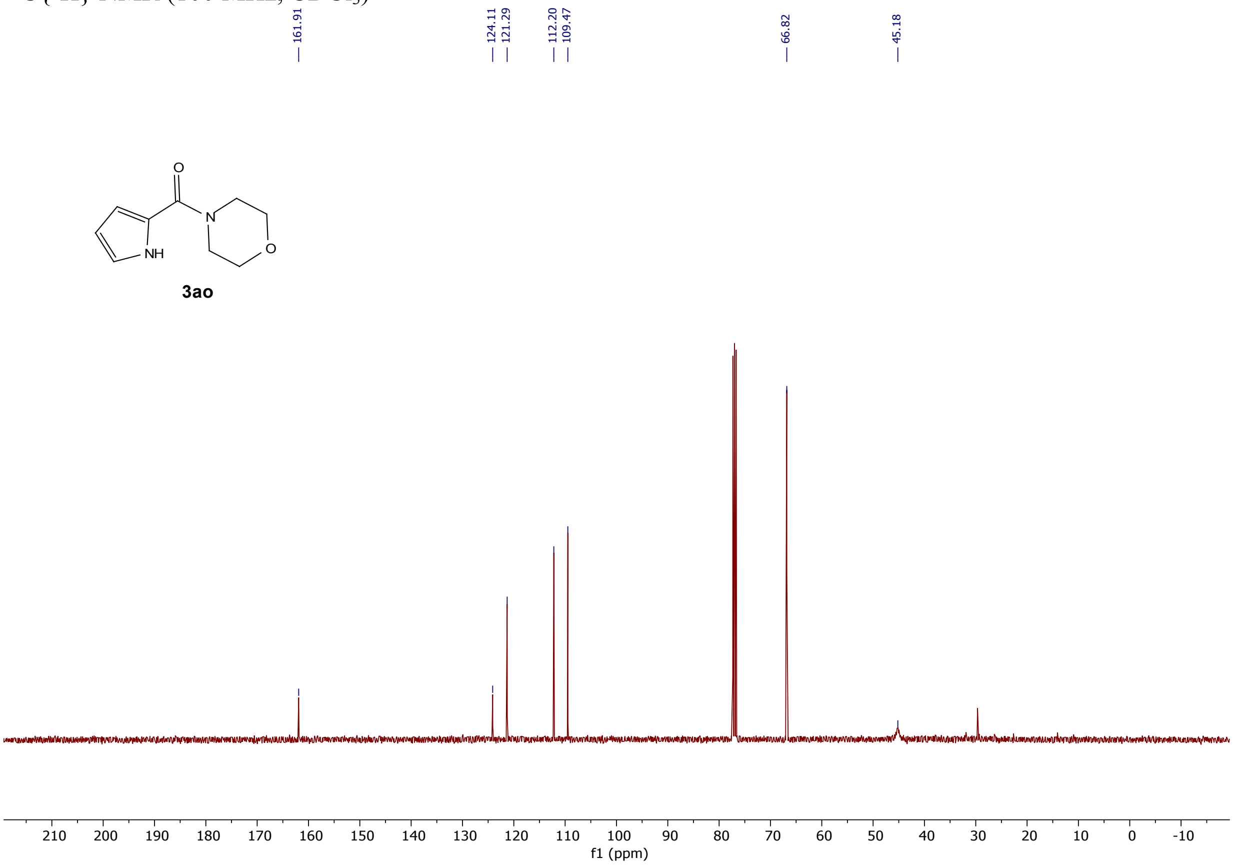

Furan-2-yl(morpholino)methanone (3ap):

^{1}H NMR (400 MHz, $CDCl_3$)

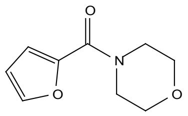

3ap

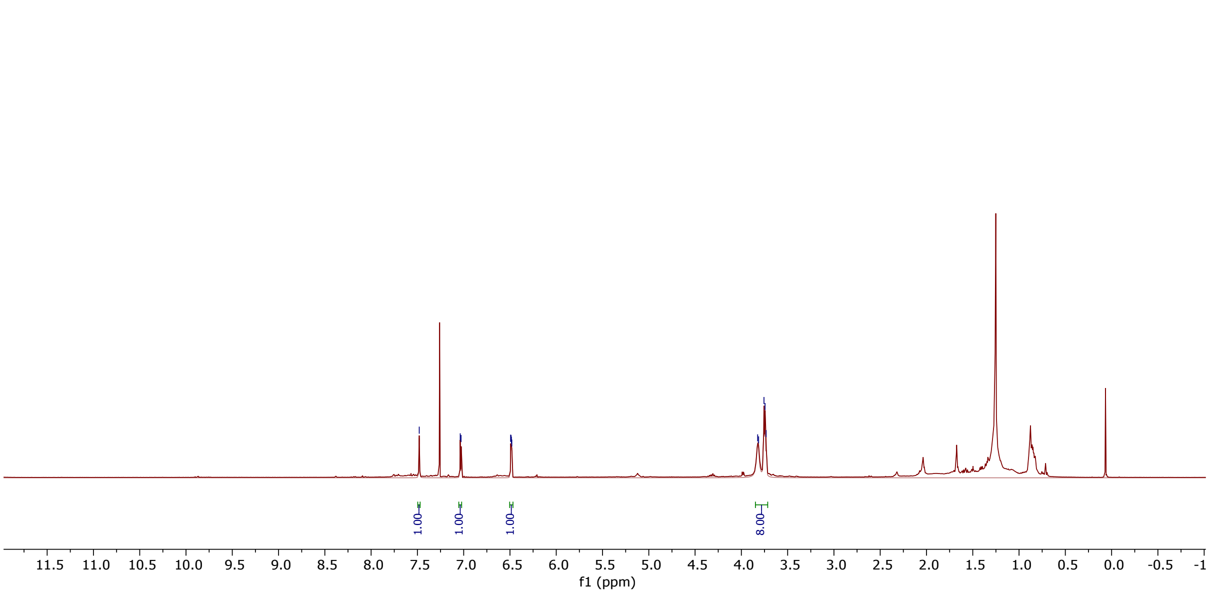

^{13}C{^{1}H} NMR (100 MHz, $CDCl_3$)

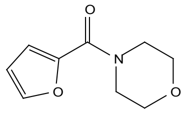

3ap

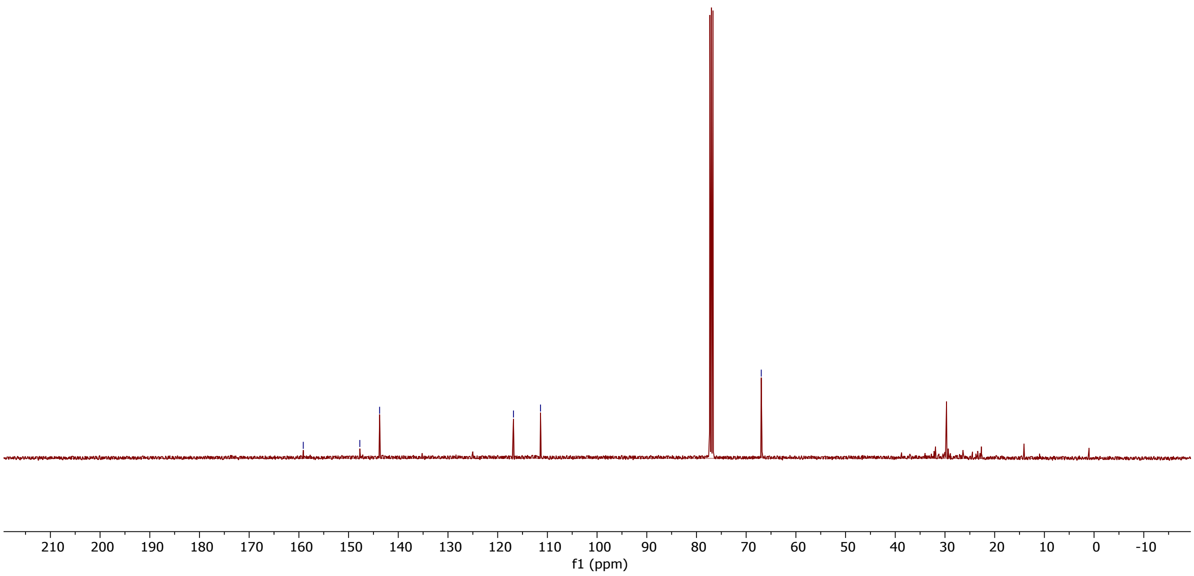

Morpholino(thiophen-2-yl)methanone (3aq):

^{1}H NMR (400 MHz, $CDCl_3$)

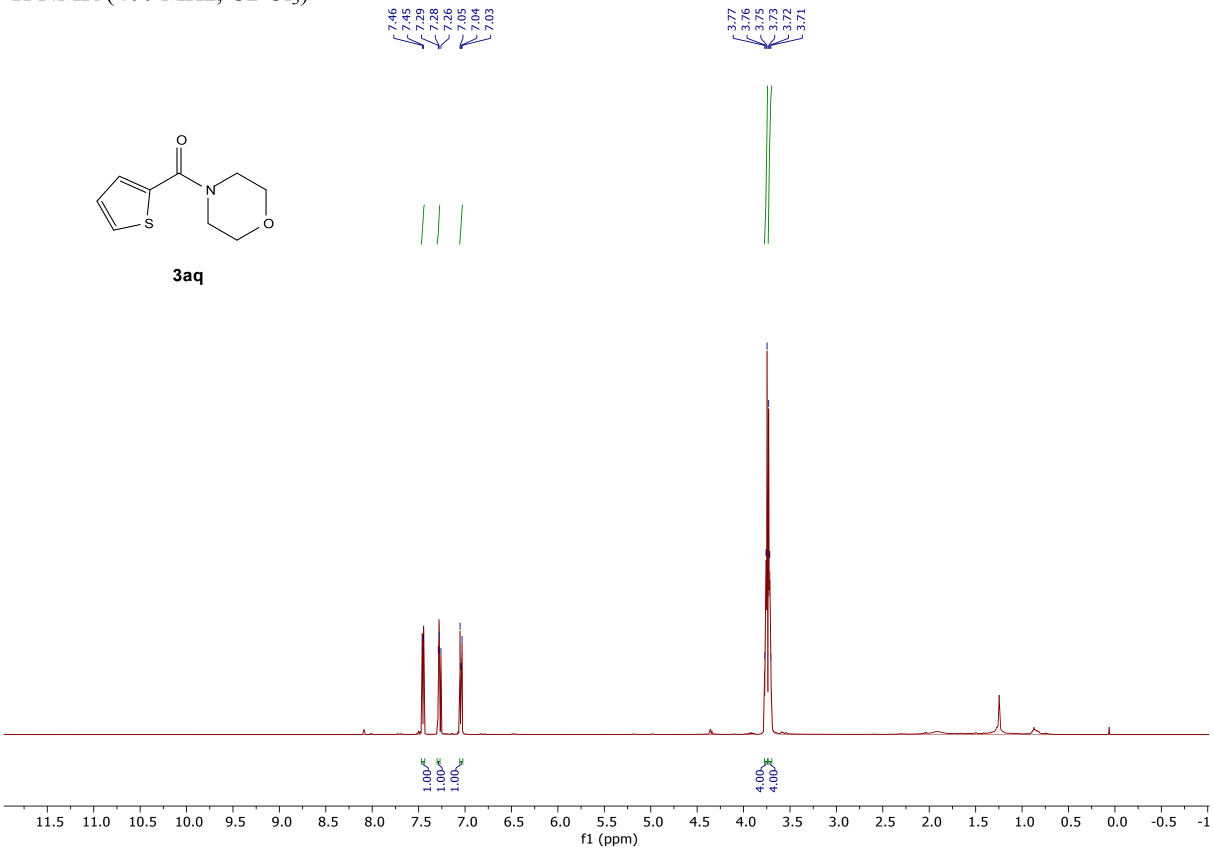

$^{13}C\{^1H\}$ NMR (100 MHz, $CDCl_3$)

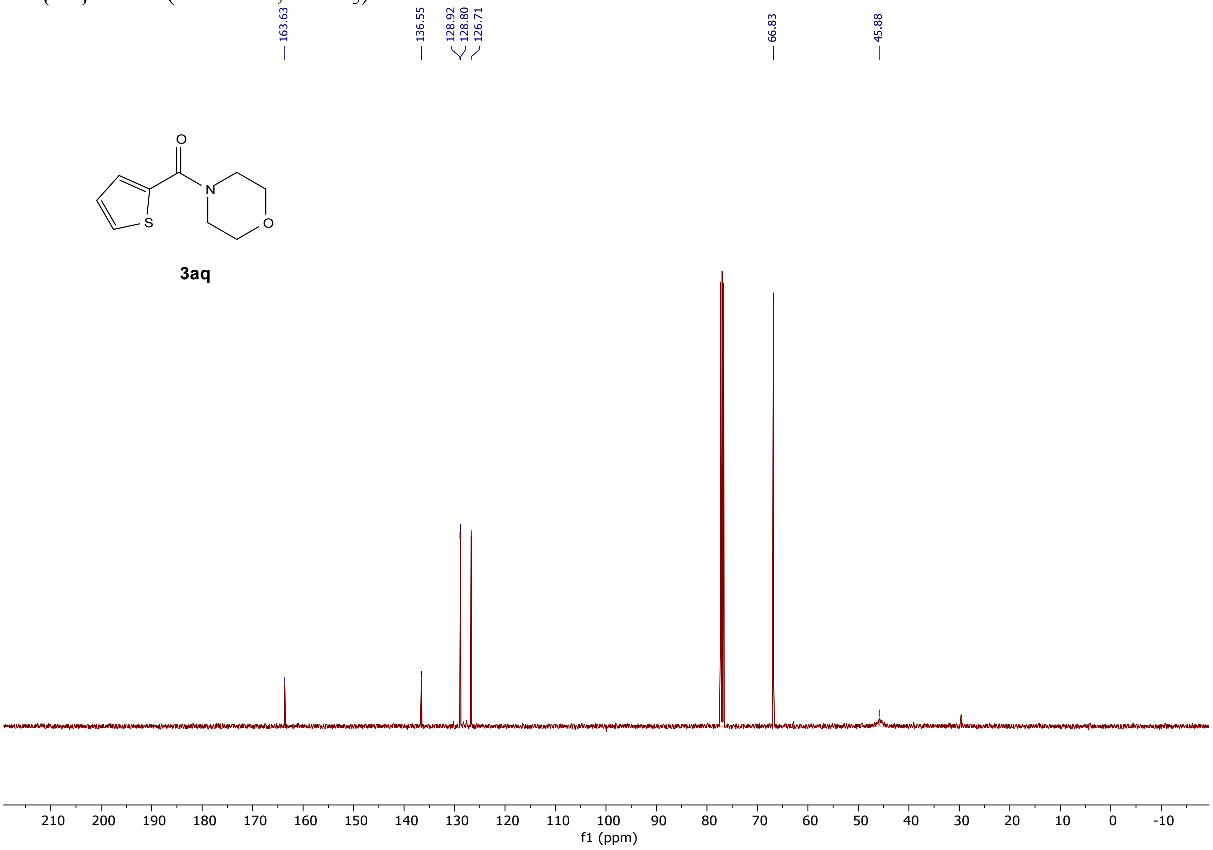

***N*-benzylthiophene-2-carboxamide (3ar):**

^{1}H NMR (400 MHz, $CDCl_3$)

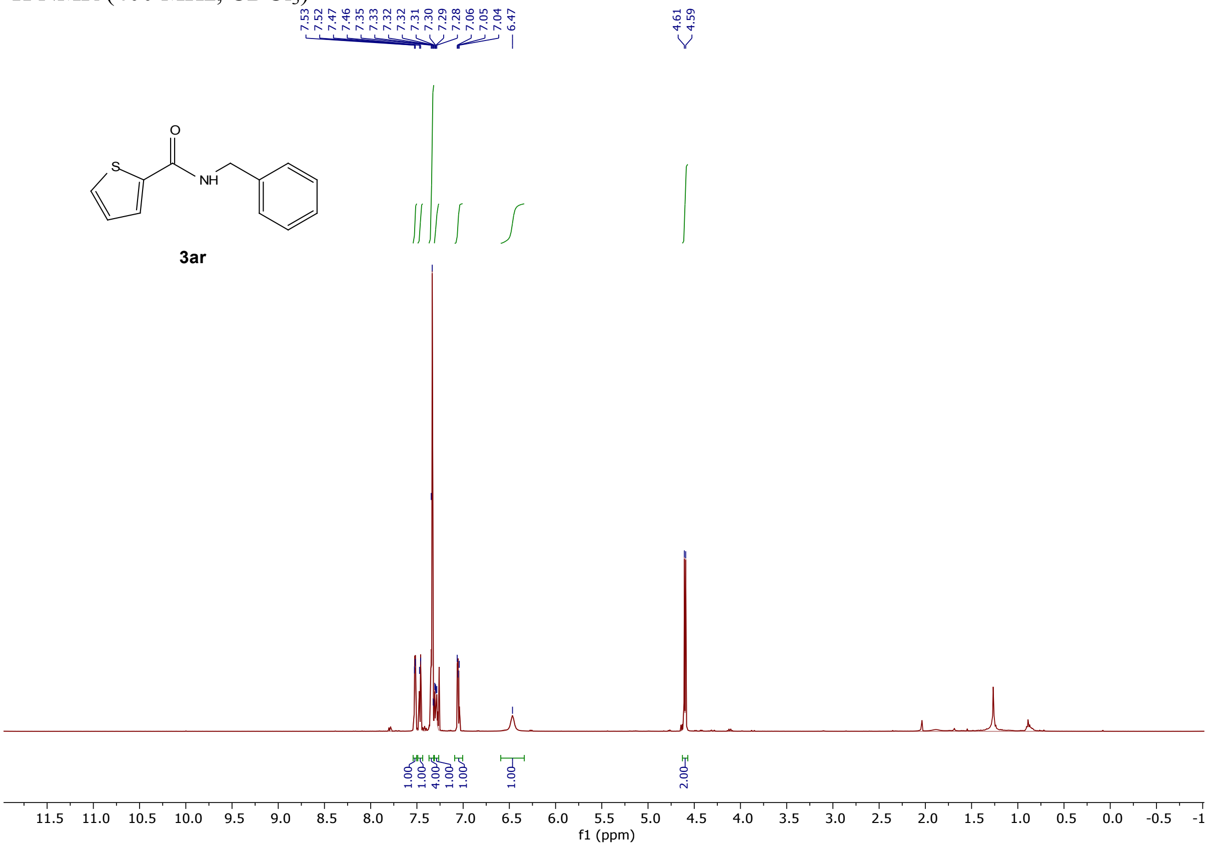

$^{13}C\{^{1}H\}$ NMR (100 MHz, $CDCl_3$)

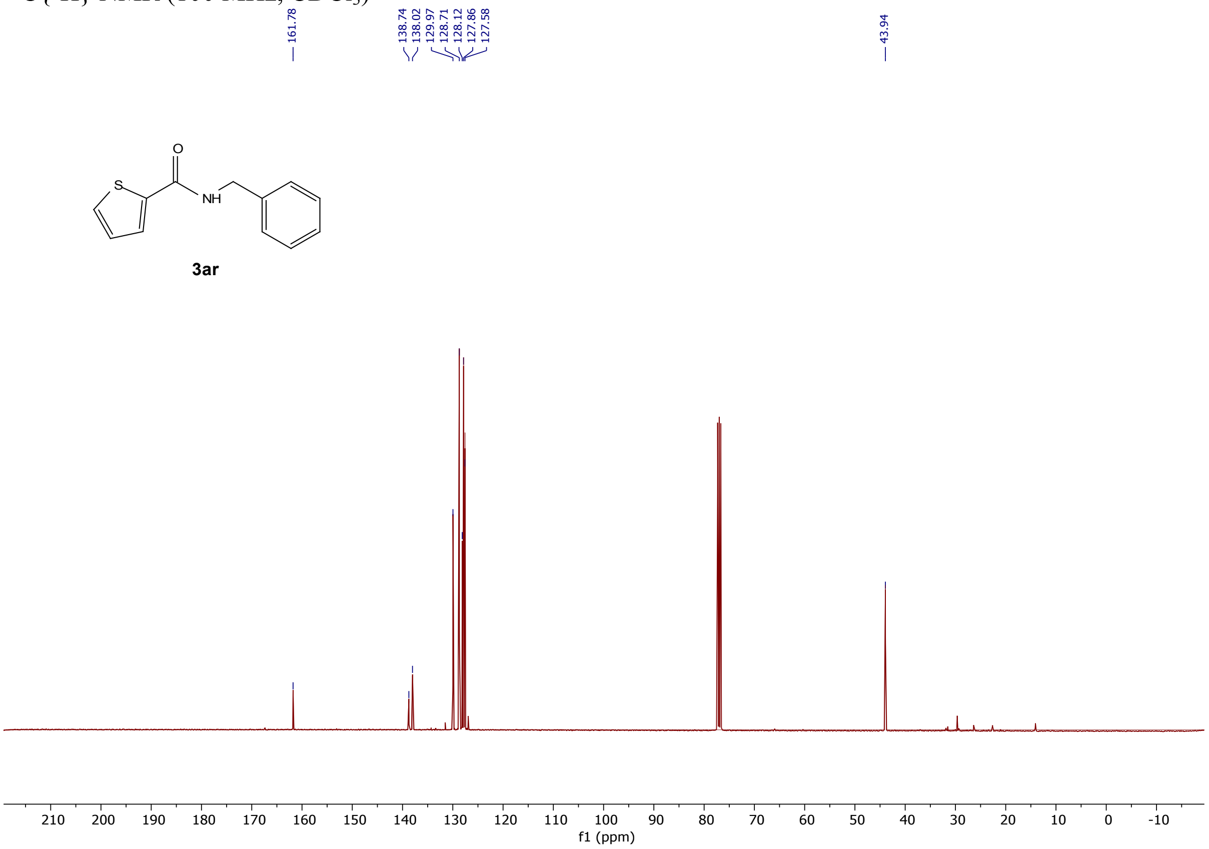

Morpholino(*p*-tolyl)methanone (3as):

^{1}H NMR (400 MHz, $CDCl_3$)

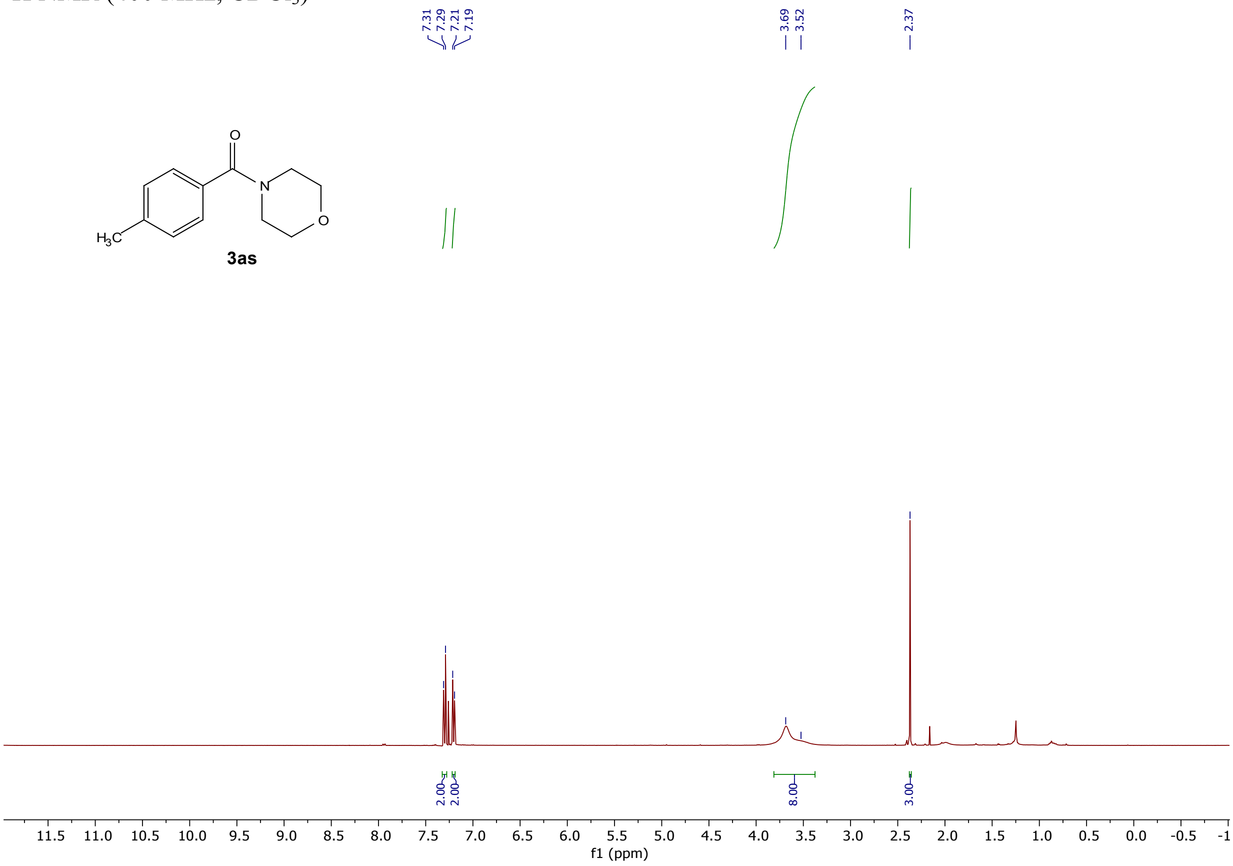

$^{13}C\{^{1}H\}$ NMR (100 MHz, $CDCl_3$)

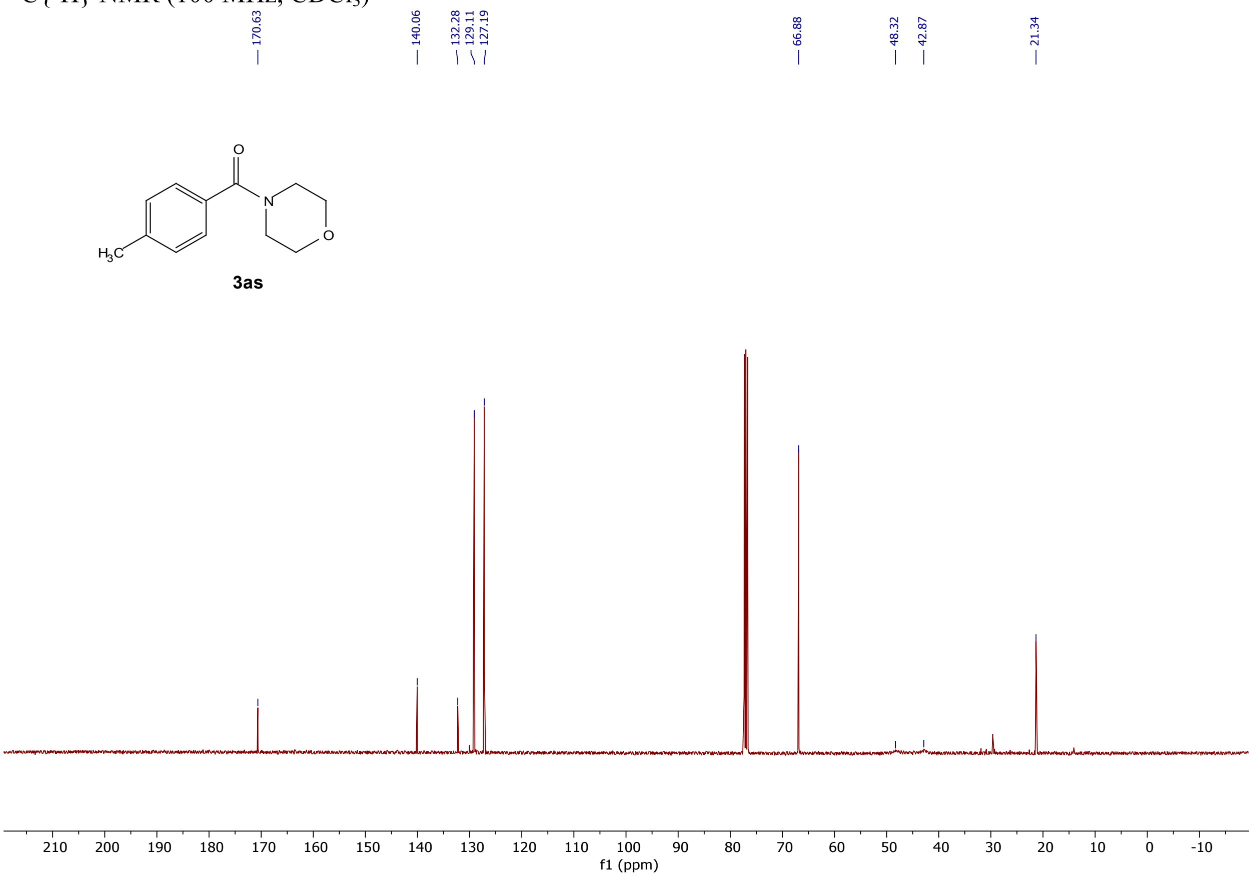

(4-chlorophenyl)(morpholino)methanone (3at):

^{1}H NMR (400 MHz, $CDCl_3$)

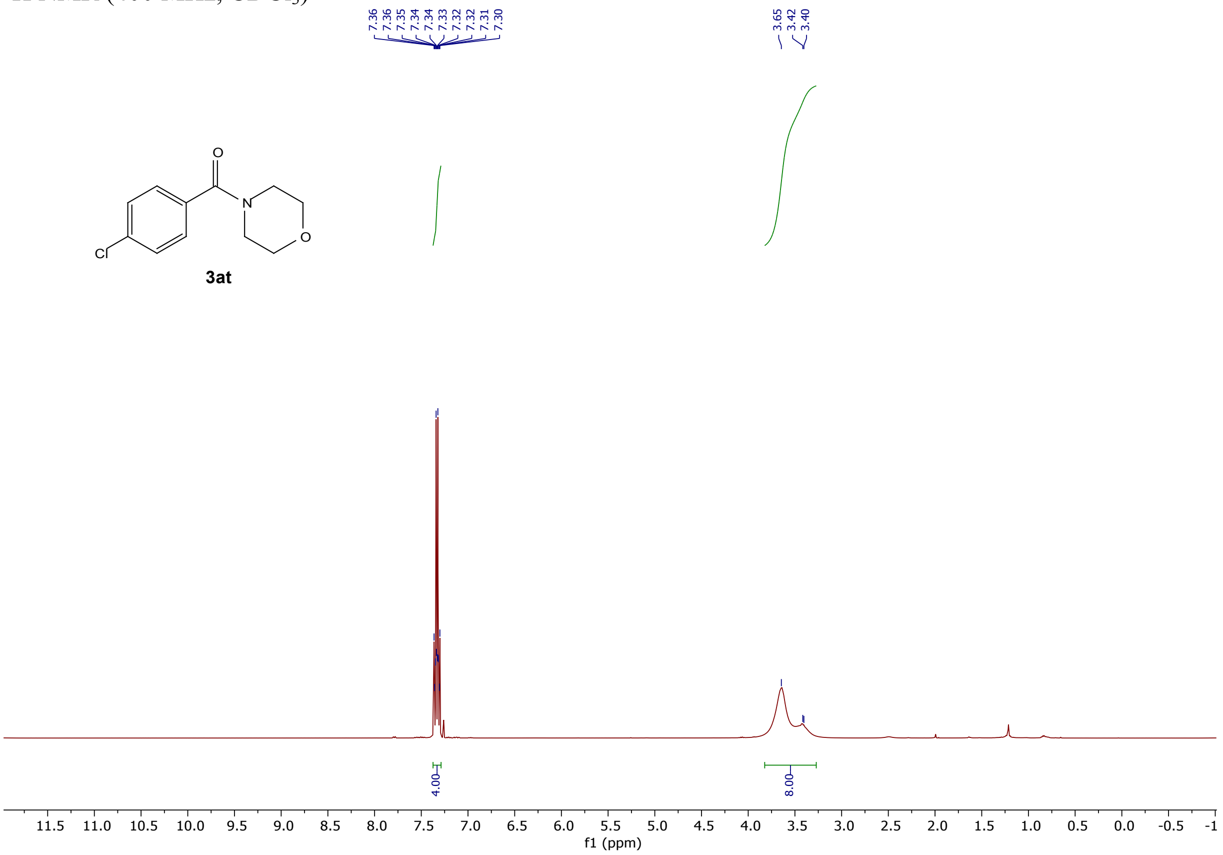

$^{13}C\{^1H\}$ NMR (100 MHz, $CDCl_3$)

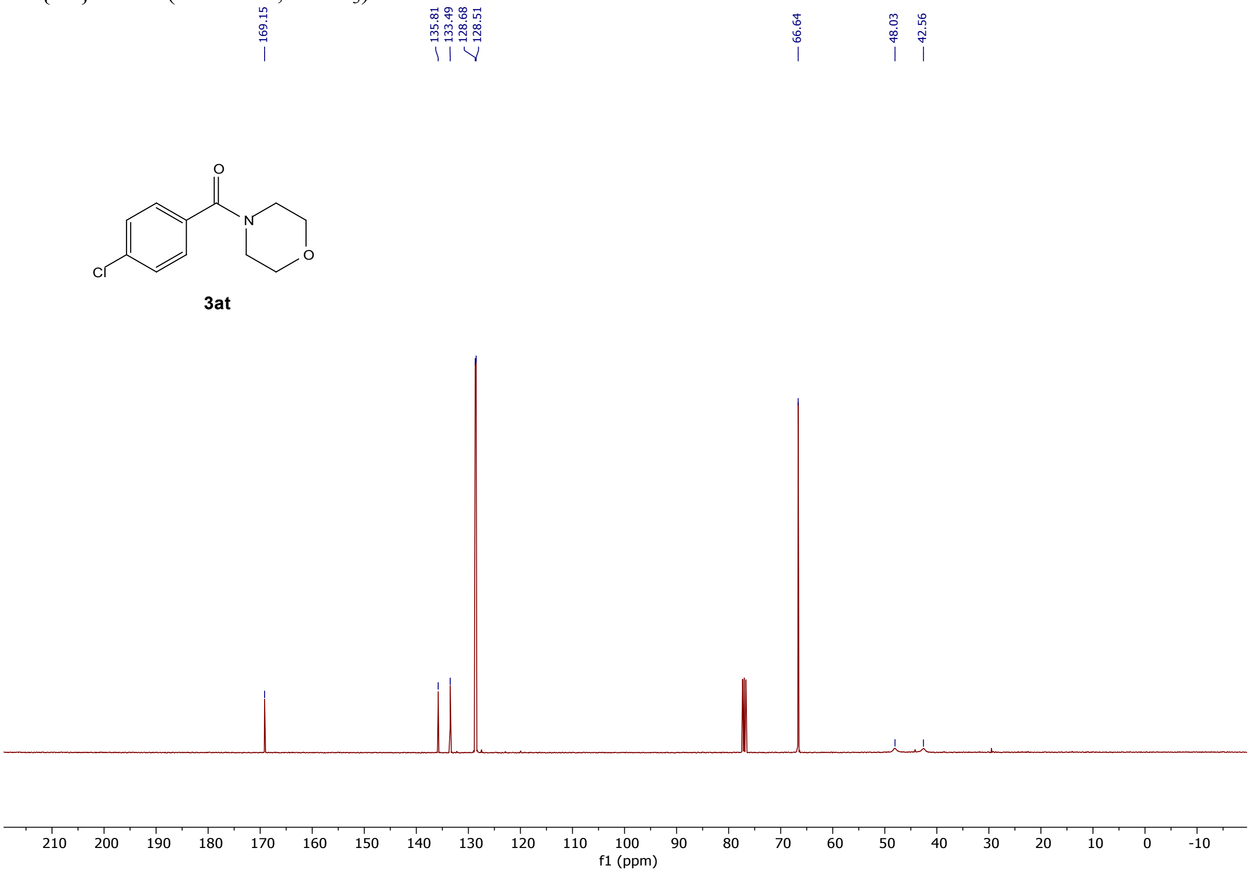

(3-chlorophenyl)(morpholino)methanone (3au):

^{1}H NMR (400 MHz, $CDCl_3$)

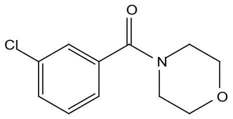

3au

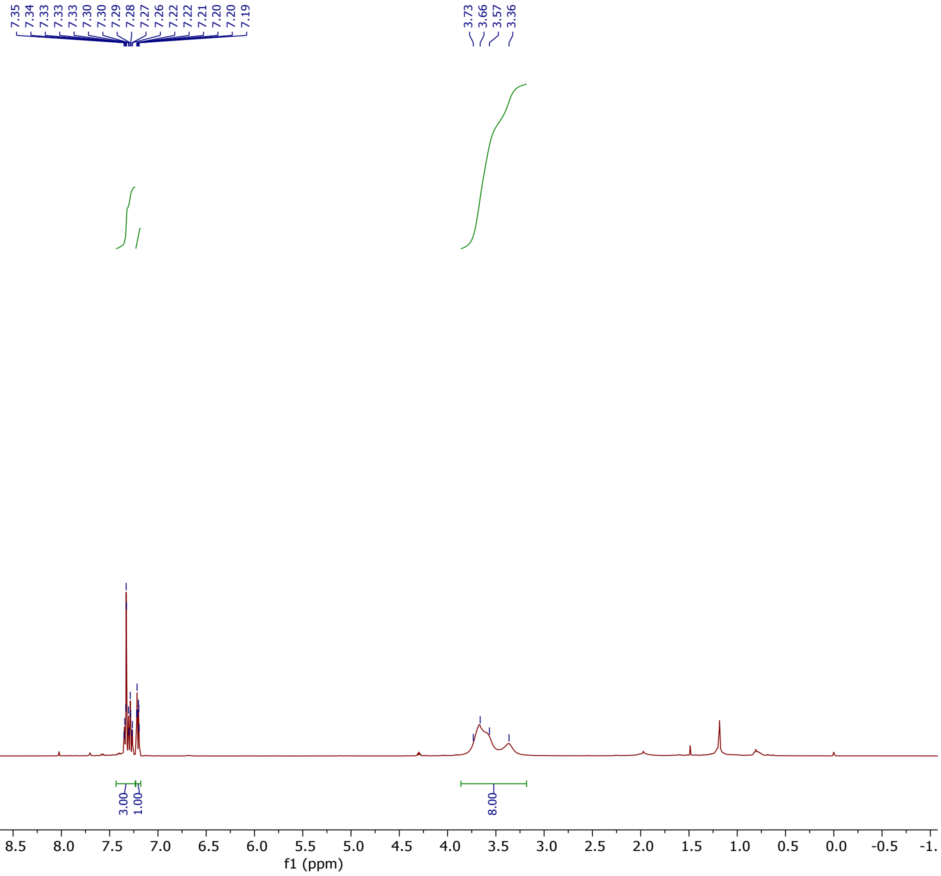

$^{13}C\{^{1}H\}$ NMR (100 MHz, $CDCl_3$)

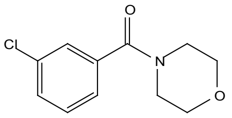

3au

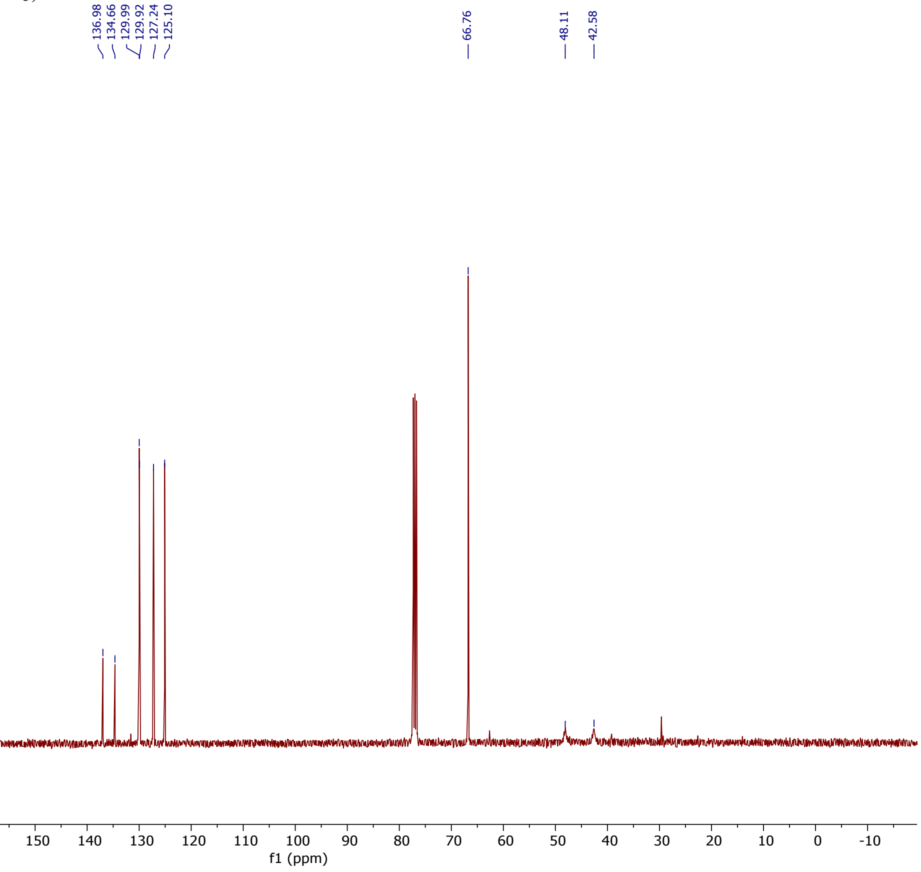

***N*-benzylbenzamide (3av):**

^{1}H NMR (400 MHz, $CDCl_3$)

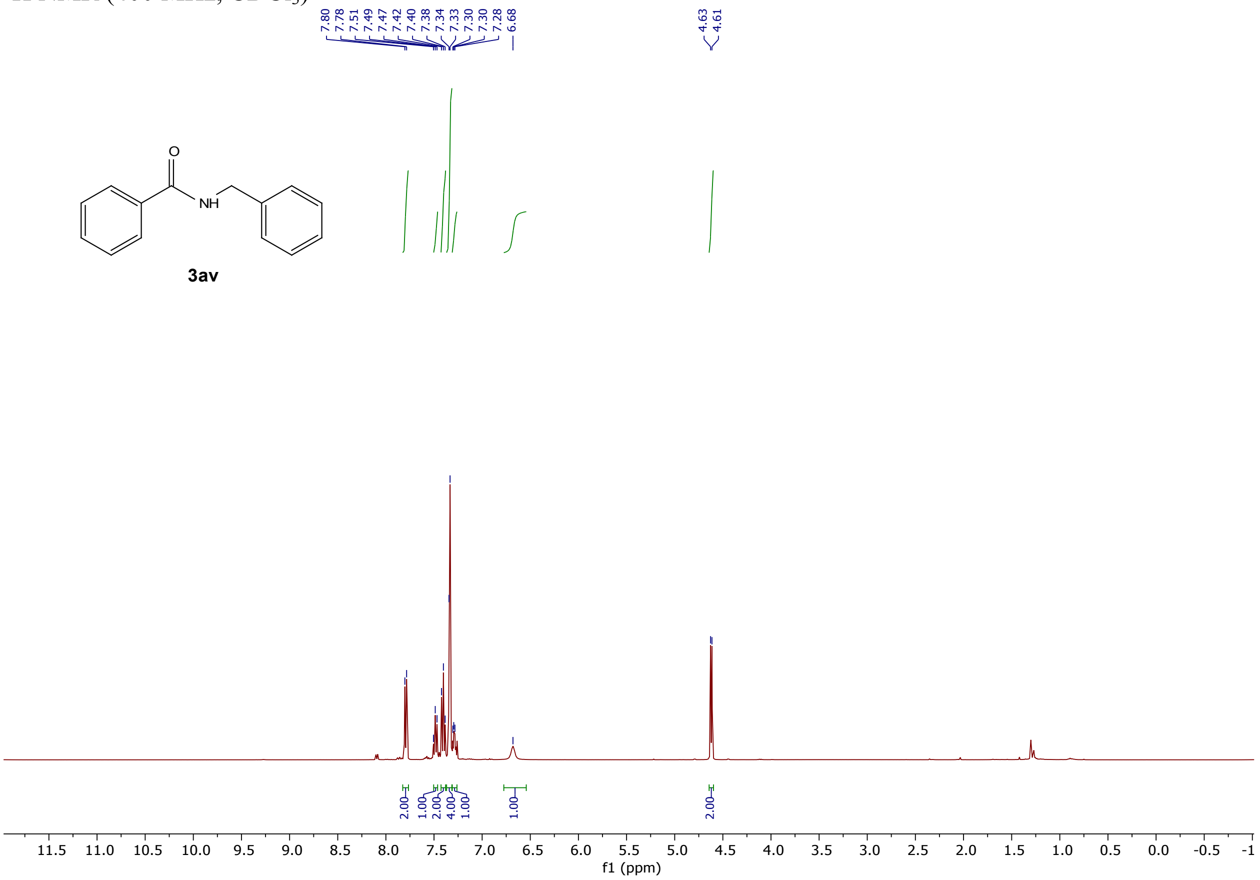

$^{13}C\{^1H\}$ NMR (100 MHz, $CDCl_3$)

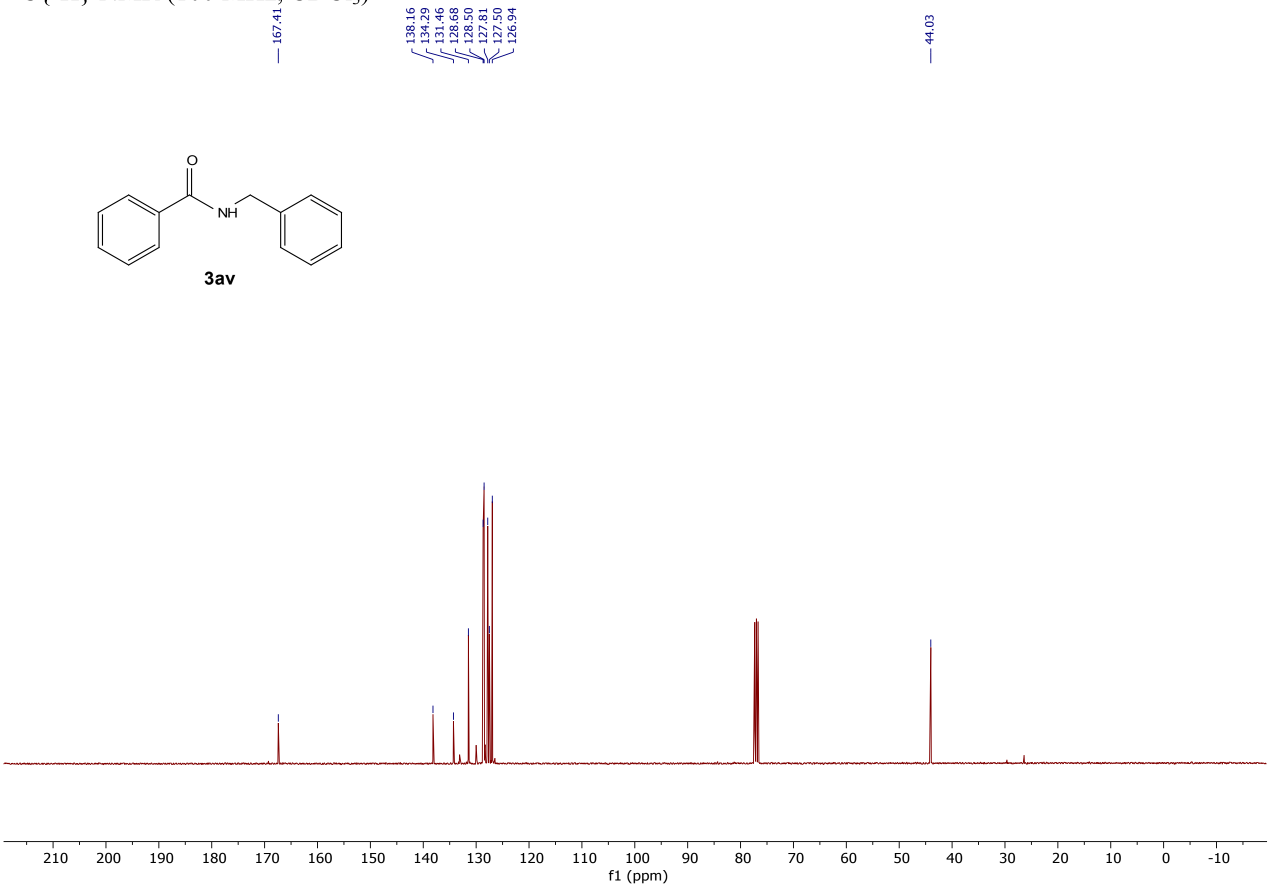

***N*-(4-bromobenzyl)benzamide (3aw):**

^{1}H NMR (400 MHz, $CDCl_3$)

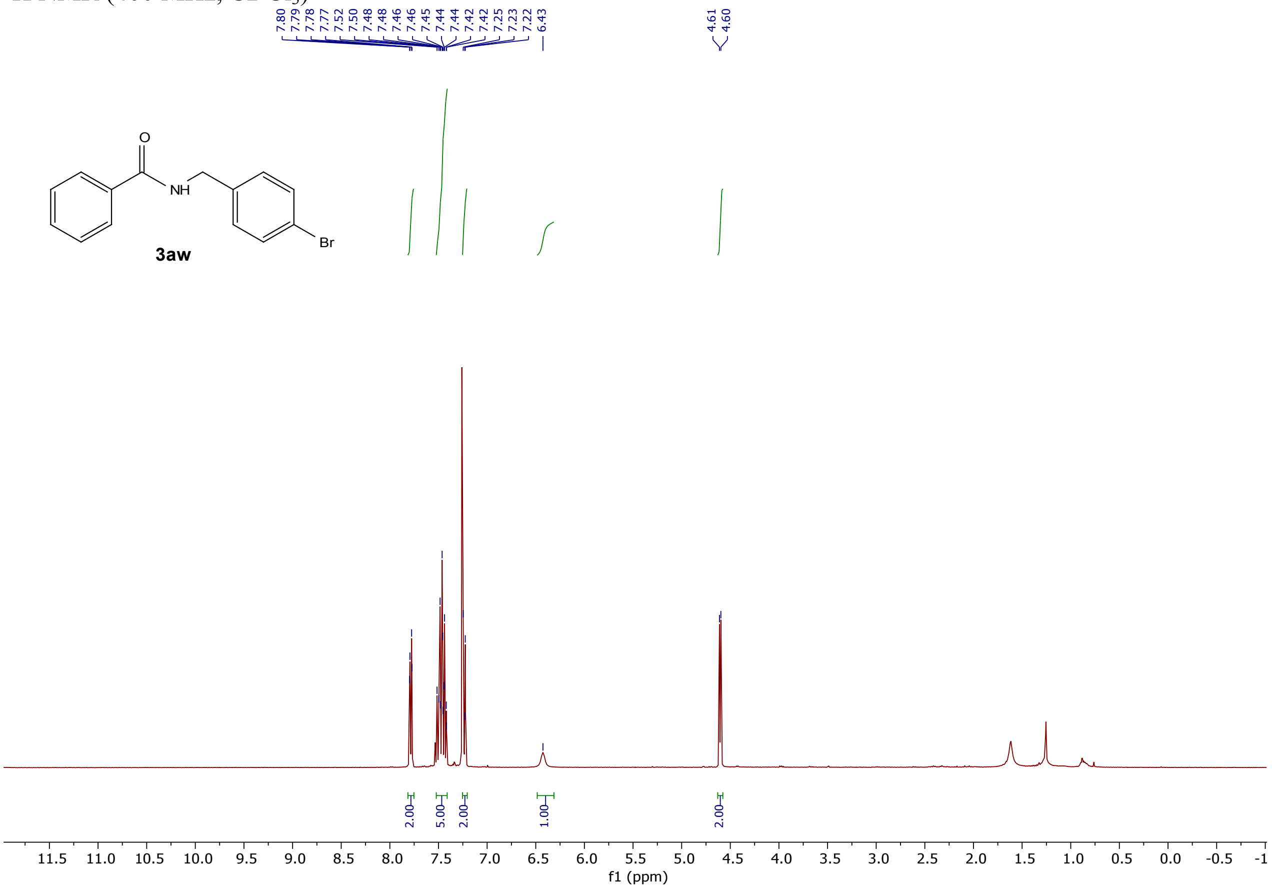

$^{13}C\{^{1}H\}$ NMR (100 MHz, $CDCl_3$)

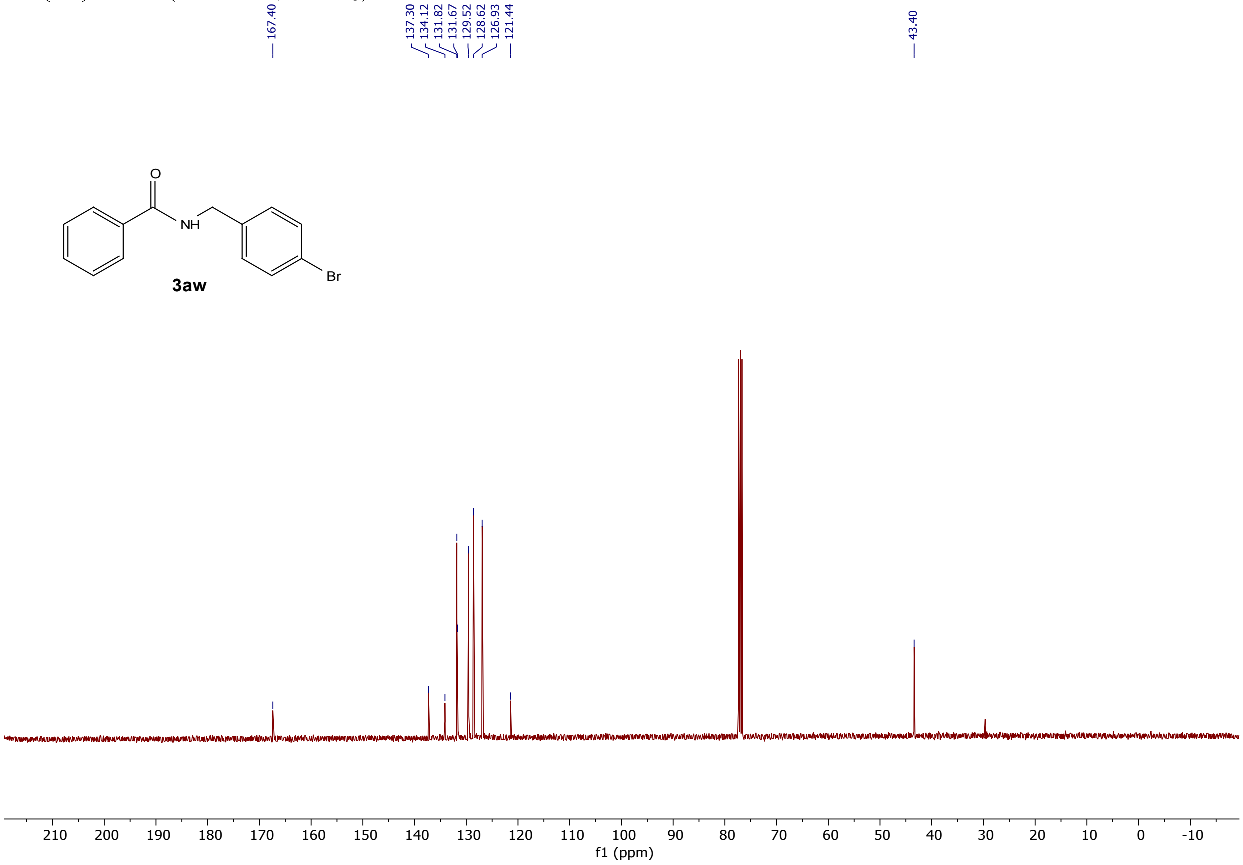

N-(4-bromobenzyl)-4-methoxybenzamide (3ax):

^{1}H NMR (400 MHz, $CDCl_3$)

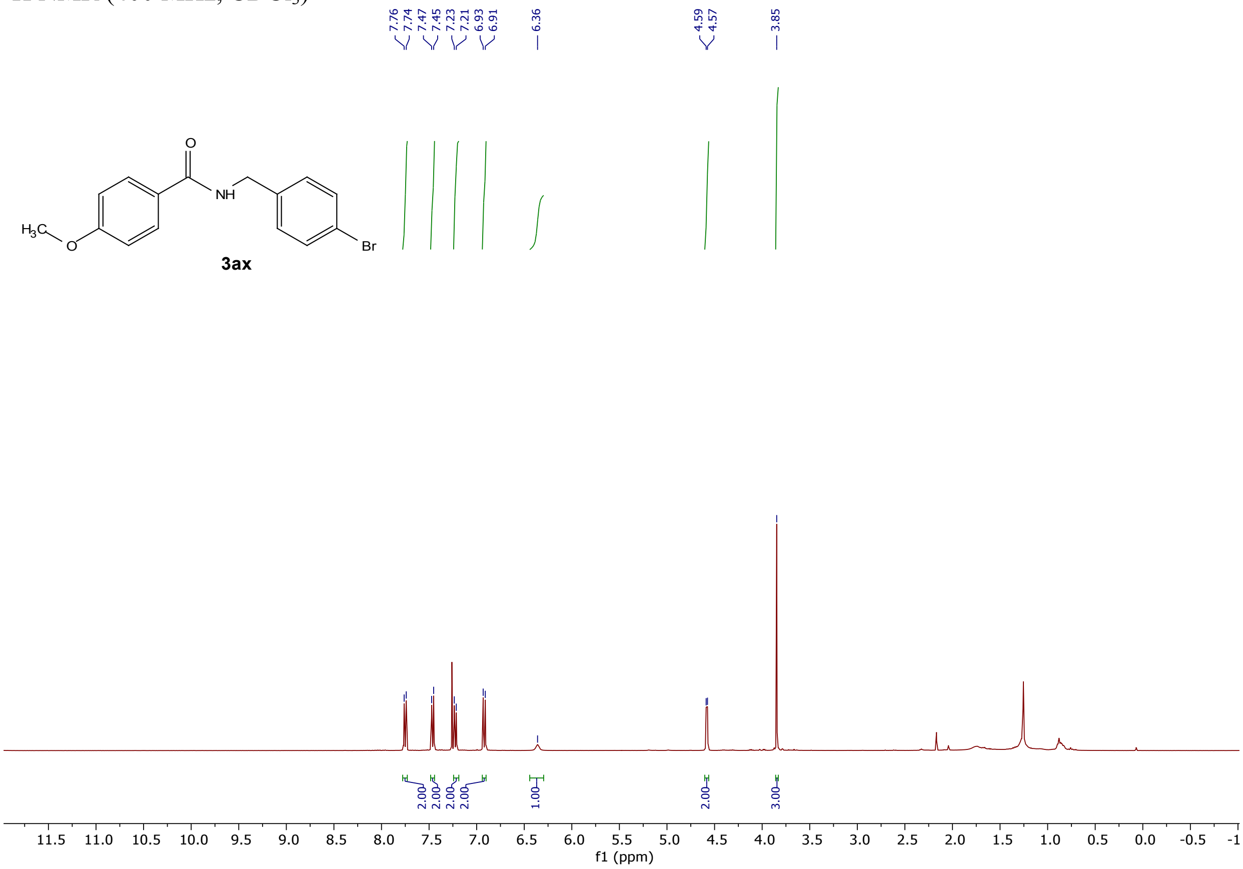

$^{13}C\{^{1}H\}$ NMR (100 MHz, $CDCl_3$)

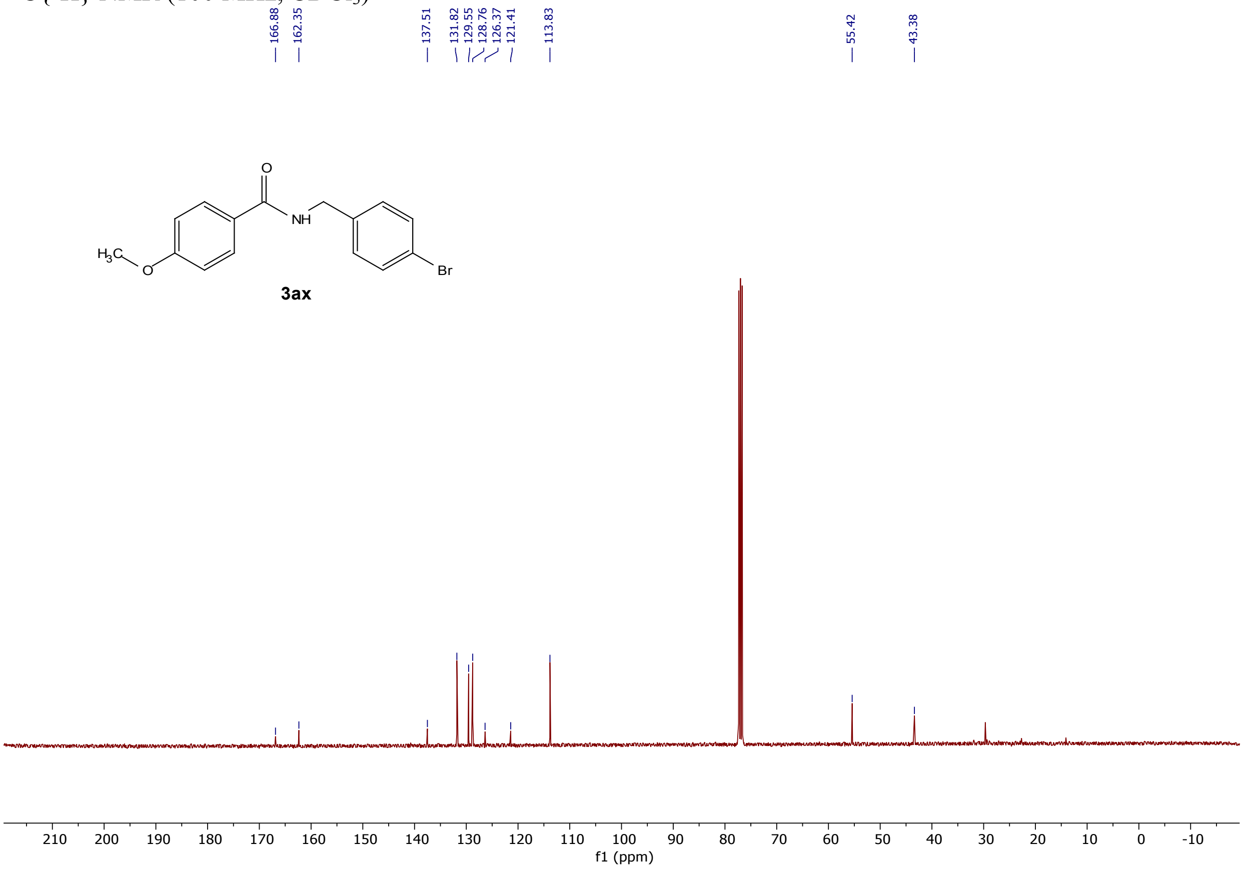

***N*-(2-chloroethyl)-[1,1'-biphenyl]-4-carboxamide (3ay):**

^{1}H NMR (400 MHz, $CDCl_3$)

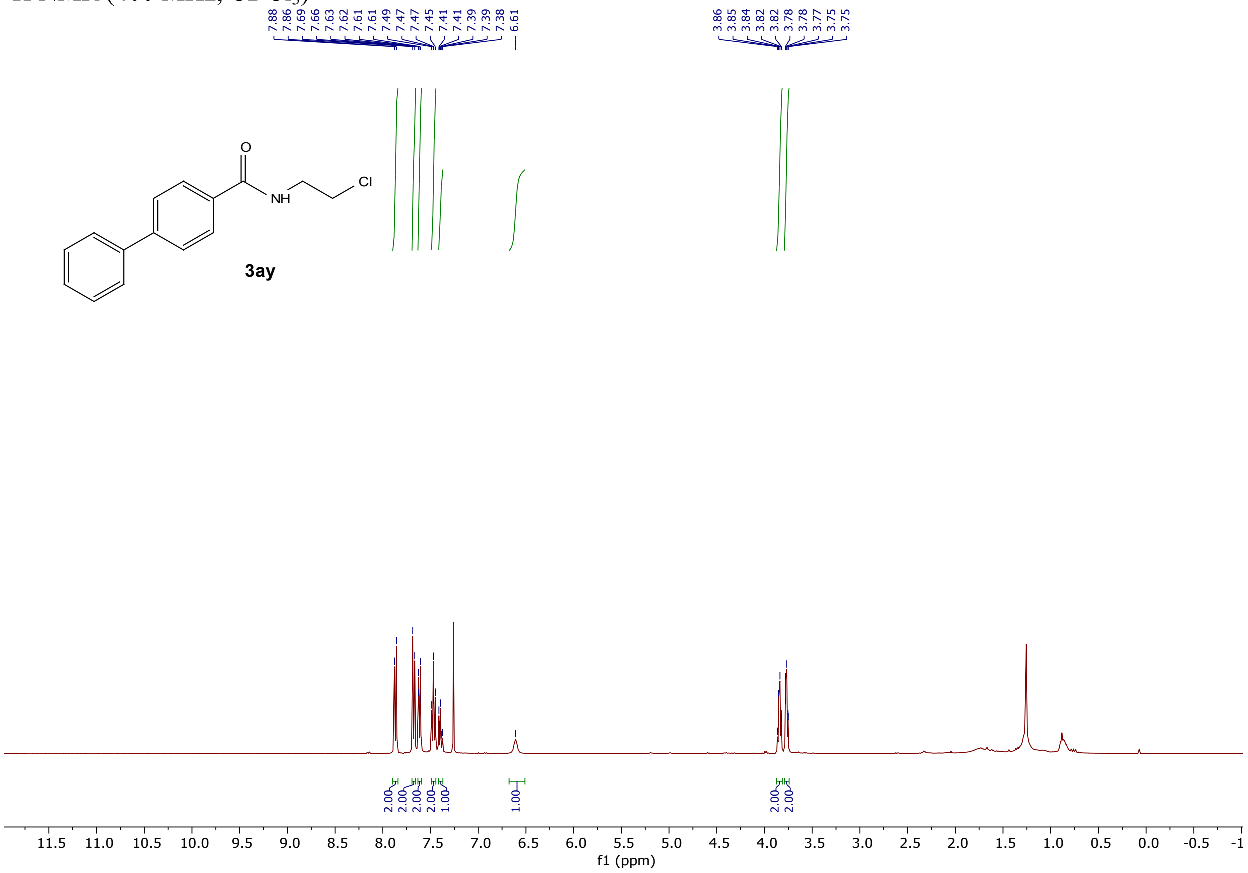

^{13}C{^{1}H} NMR (100 MHz, $CDCl_3$)

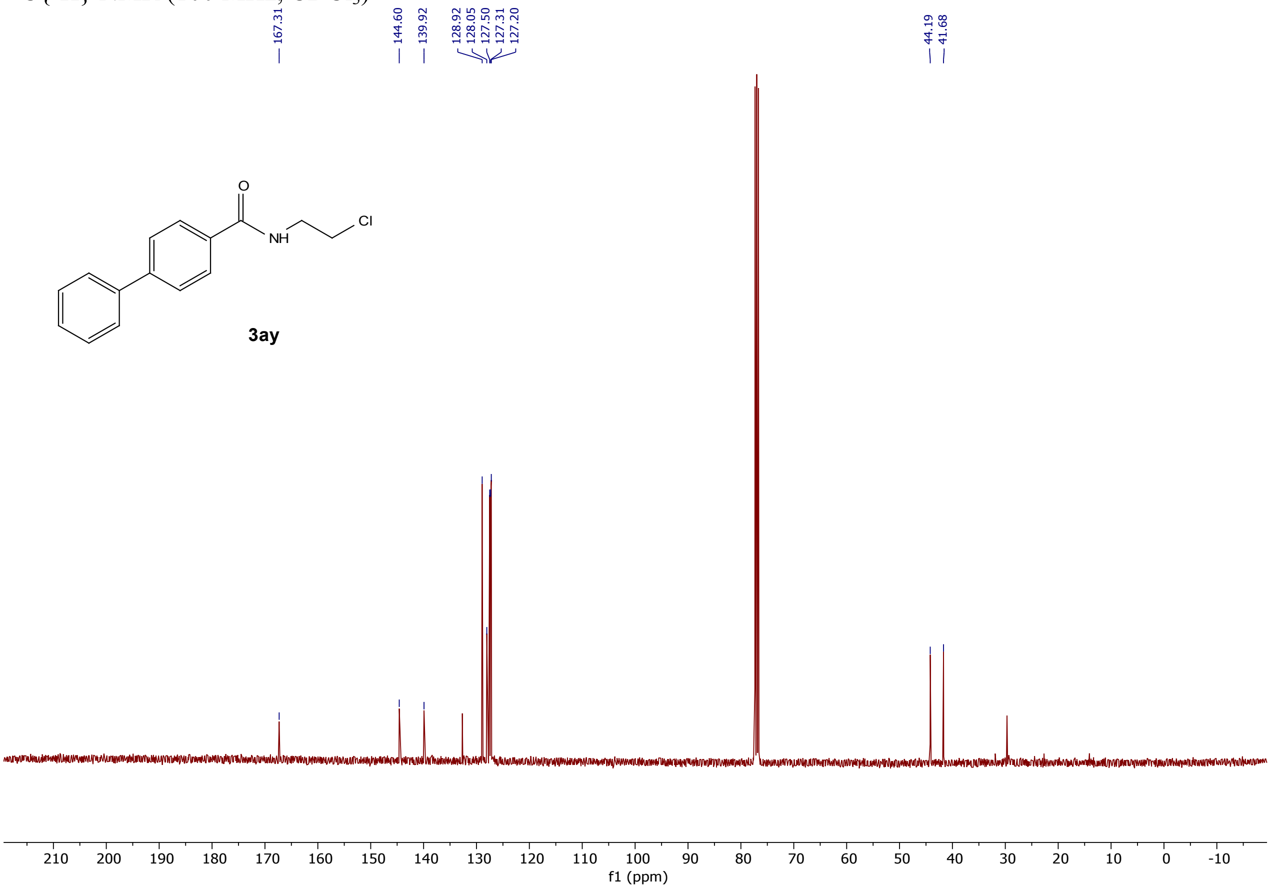

***N*-benzyl-4-chlorobenzamide (3az):**

^{1}H NMR (400 MHz, $CDCl_3$)

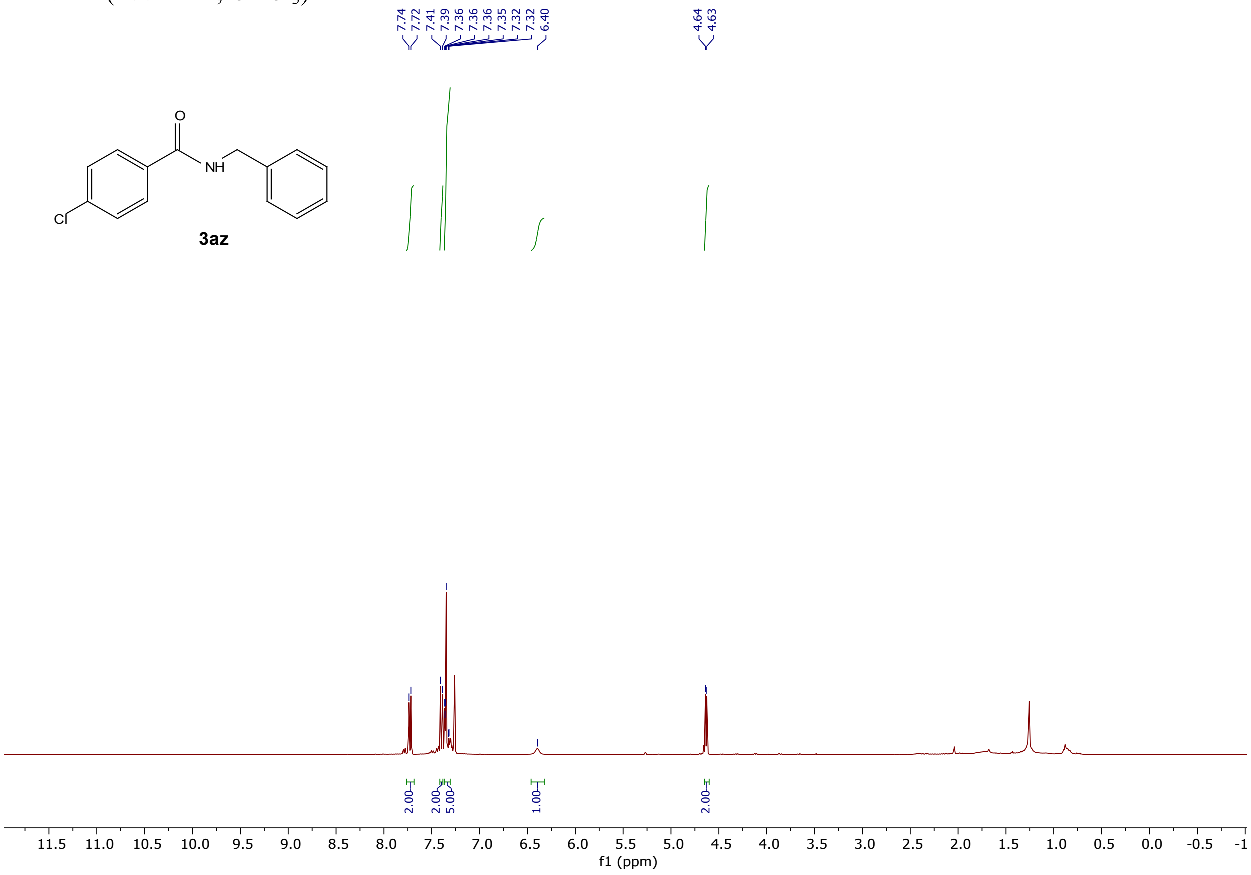

$^{13}C\{^{1}H\}$ NMR (100 MHz, $CDCl_3$)

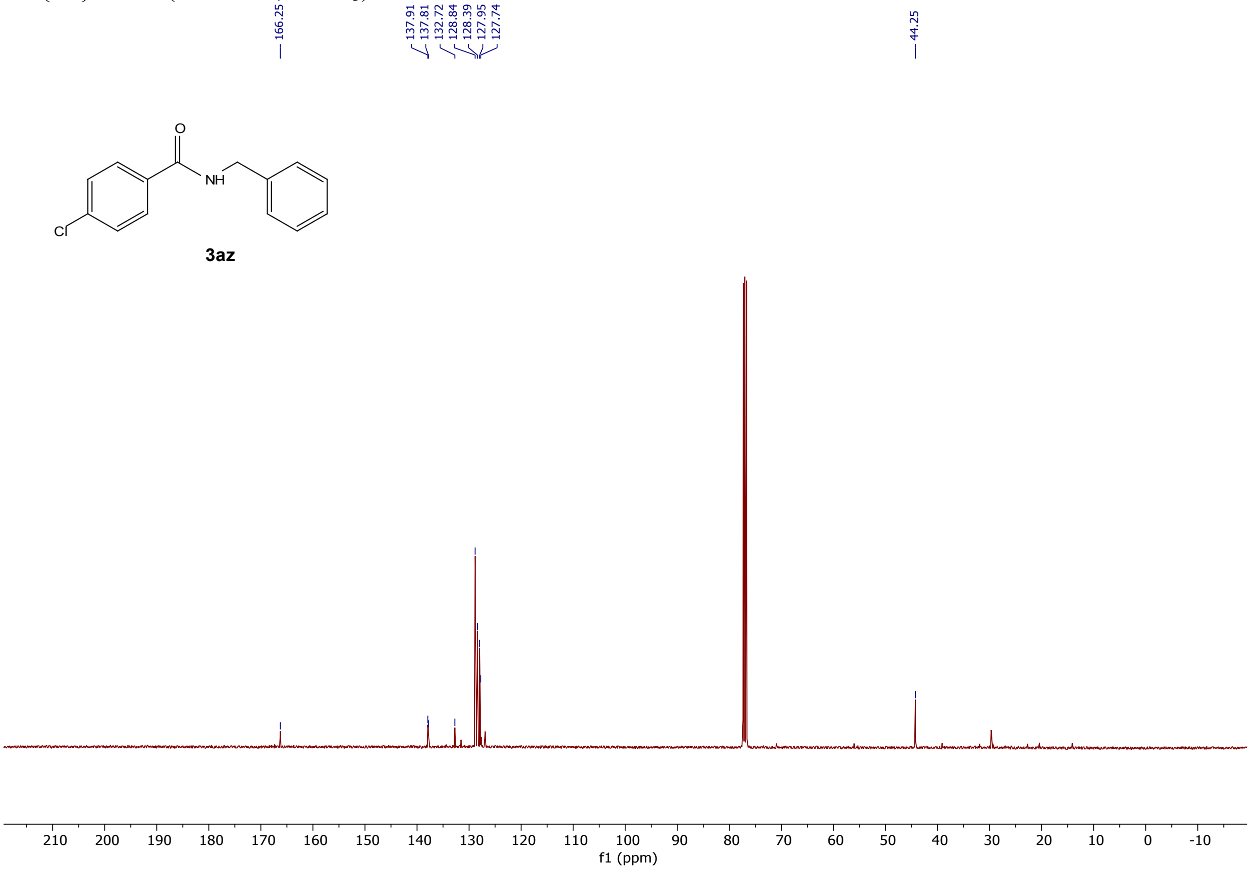

N-(2-chloroethyl)-4-nitrobenzamide (3ba):

^{1}H NMR (400 MHz, $CDCl_3$)

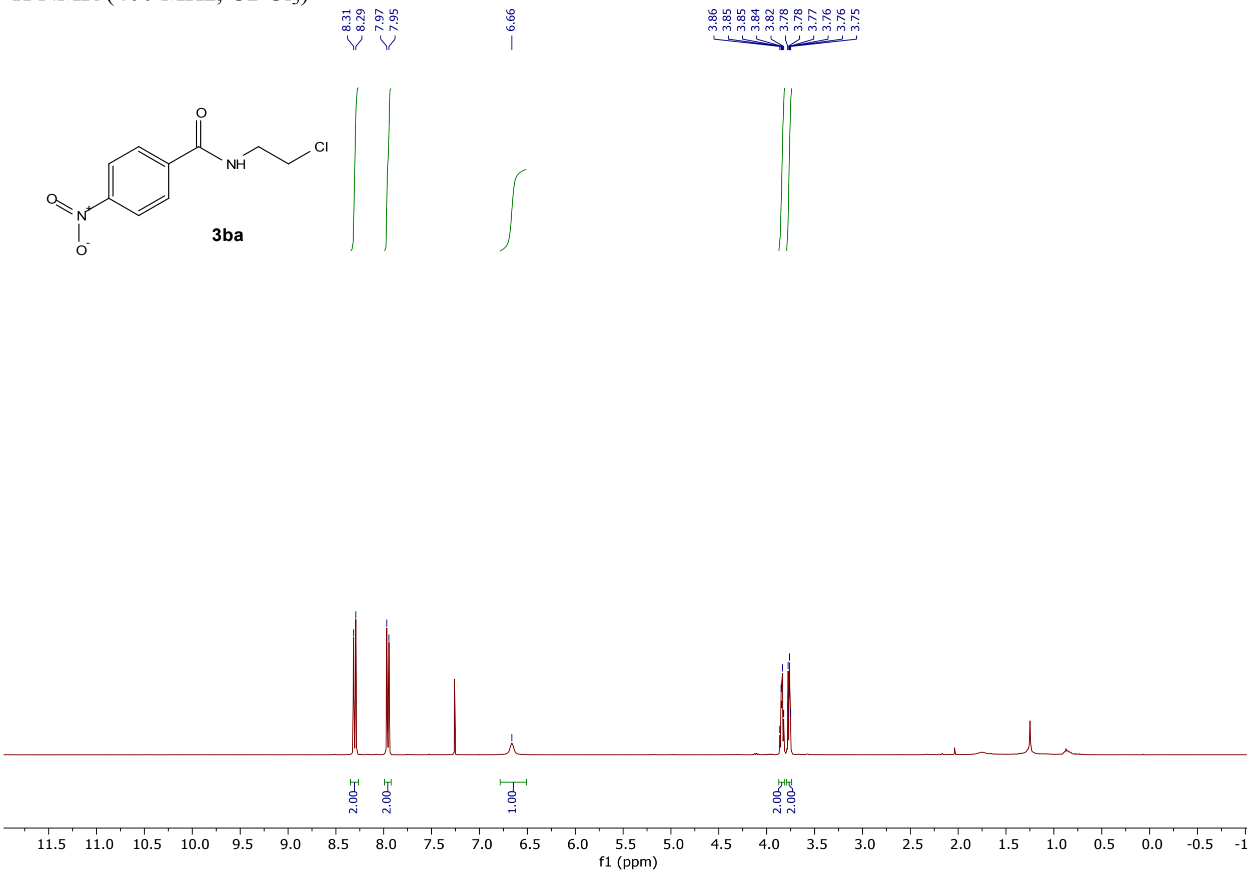

$^{13}C\{^{1}H\}$ NMR (100 MHz, $CDCl_3$)

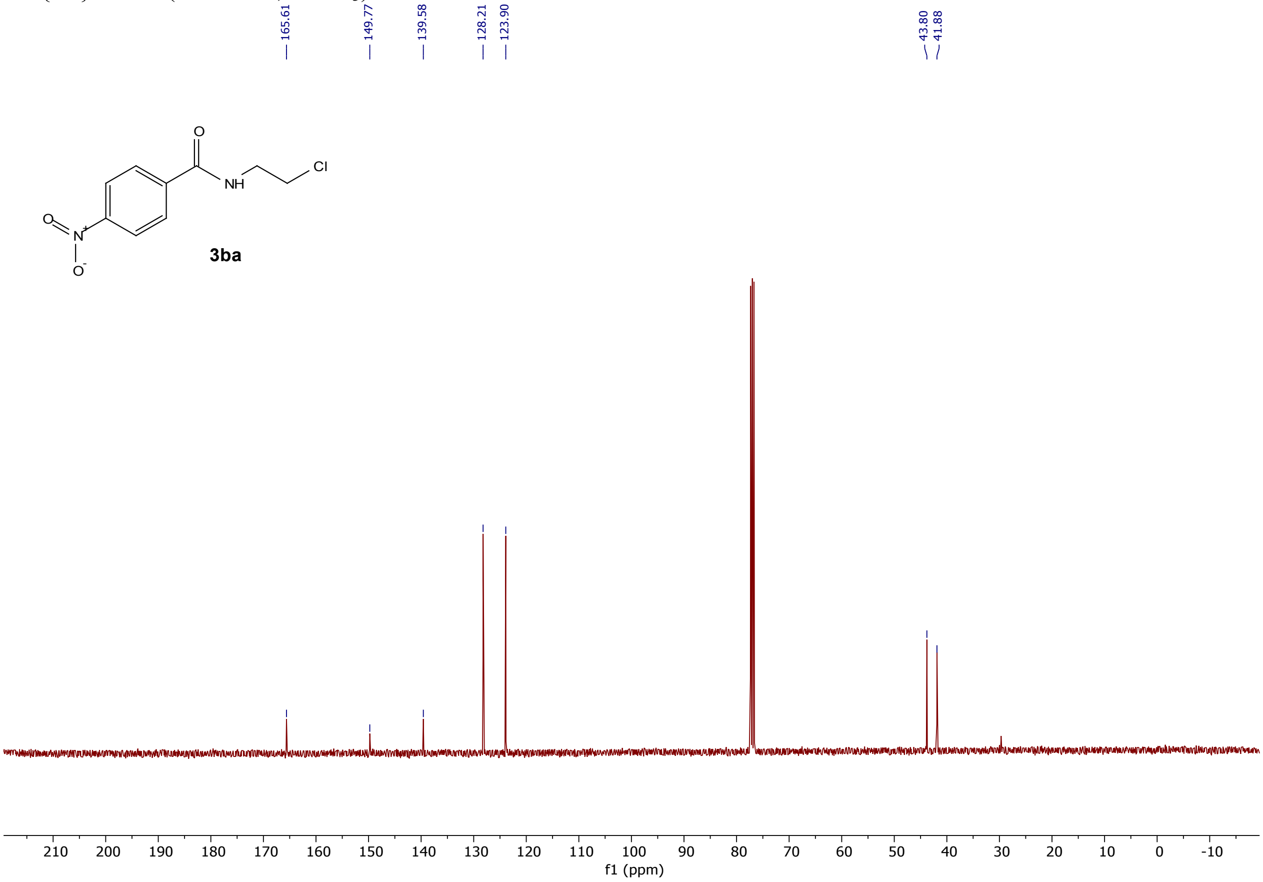

***N*-(*tert*-butyl)benzamide (3bb):**

^1H NMR (400 MHz, $CDCl_3$)

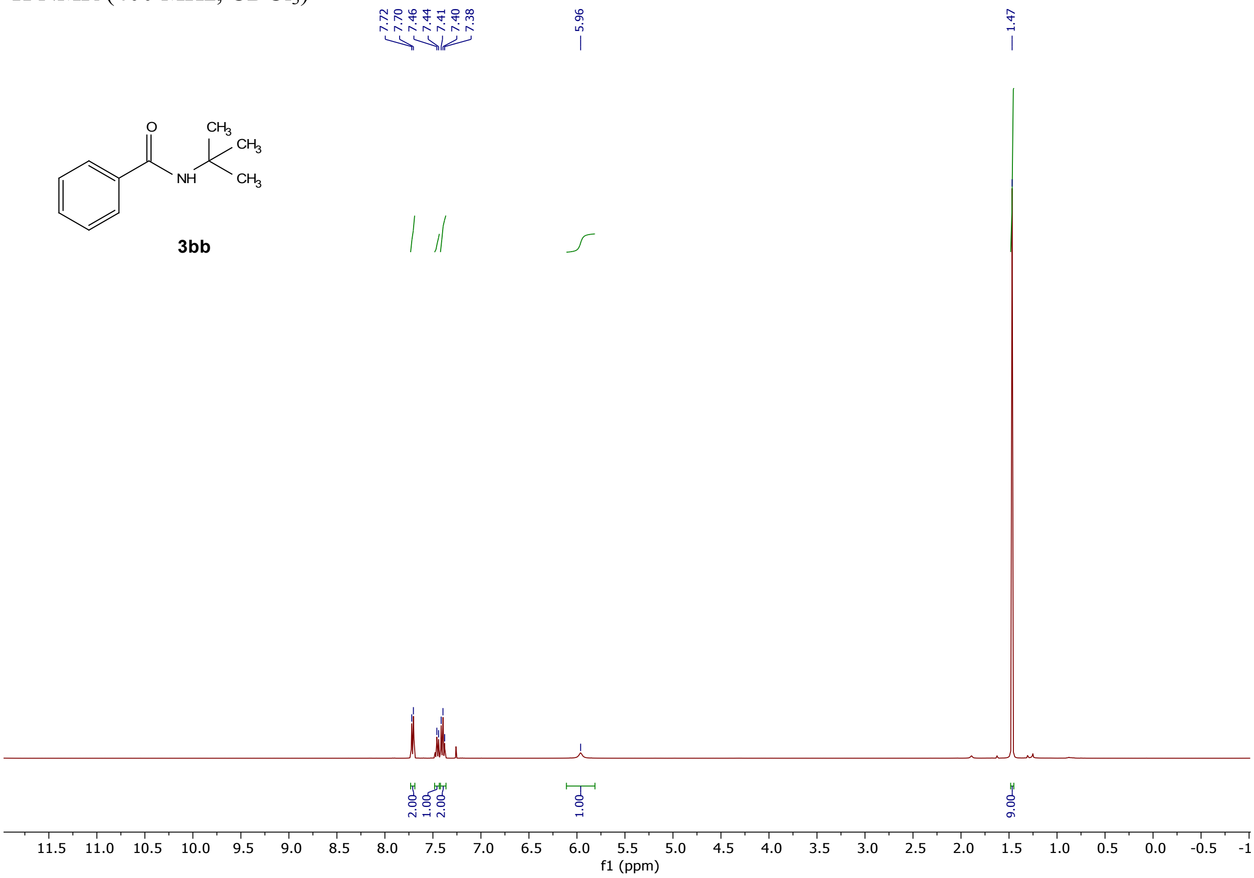

$^{13}C\{^1H\}$ NMR (100 MHz, $CDCl_3$)

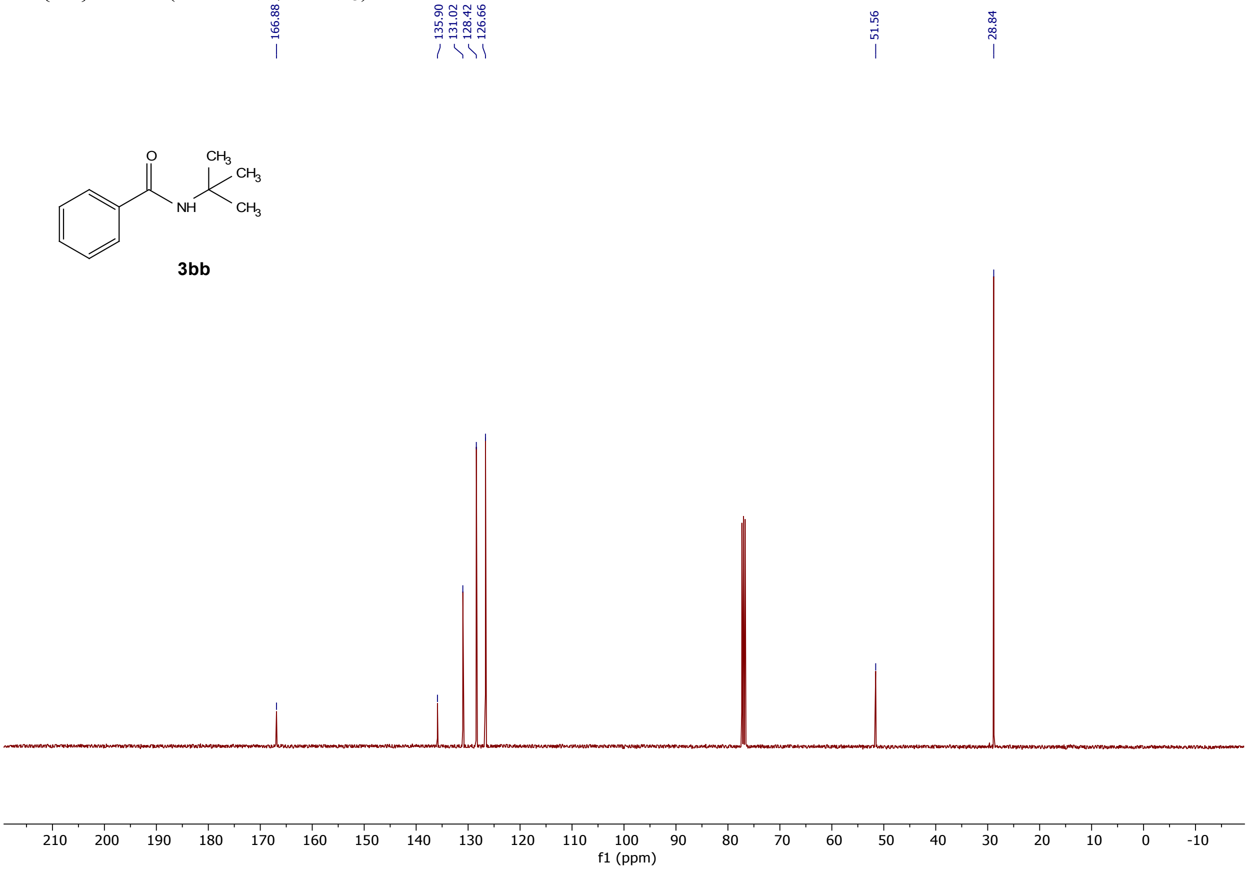

Benzamide (3bc):

^{1}H NMR (400 MHz, $CDCl_3$)

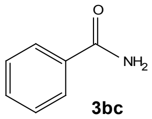

3bc

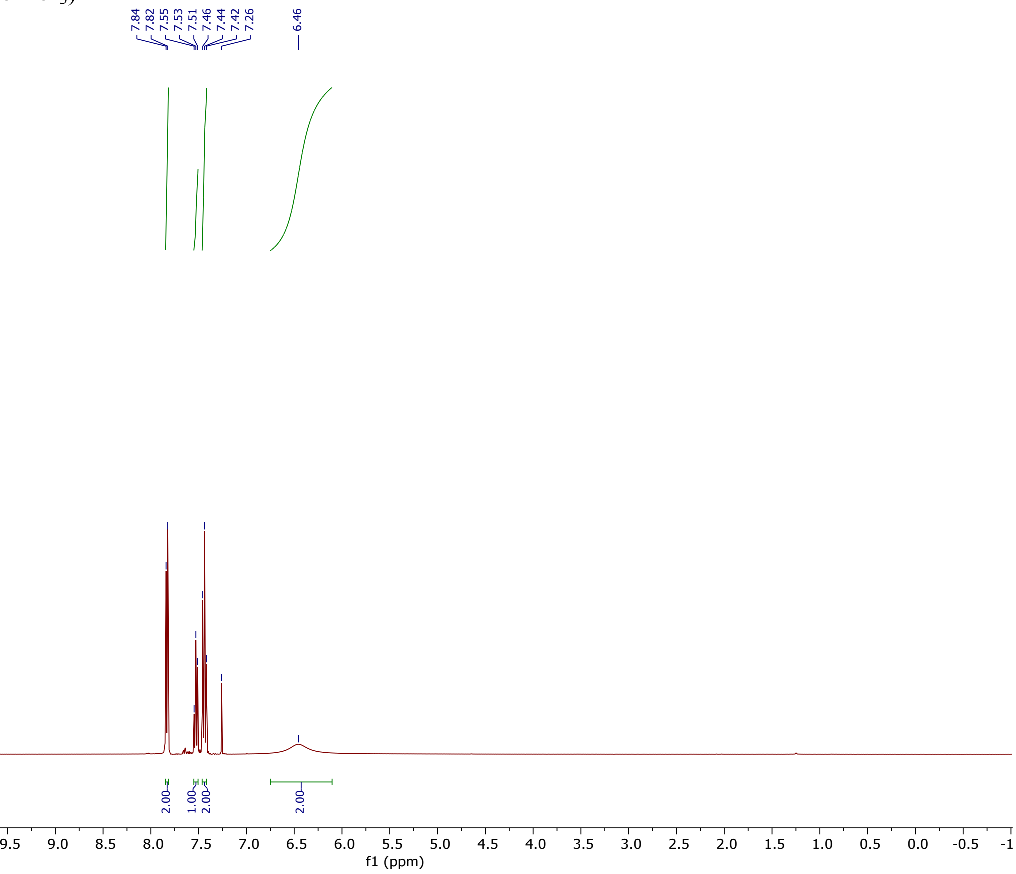

$^{13}C\{^{1}H\}$ NMR (100 MHz, $CDCl_3$)

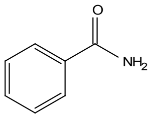

3bc

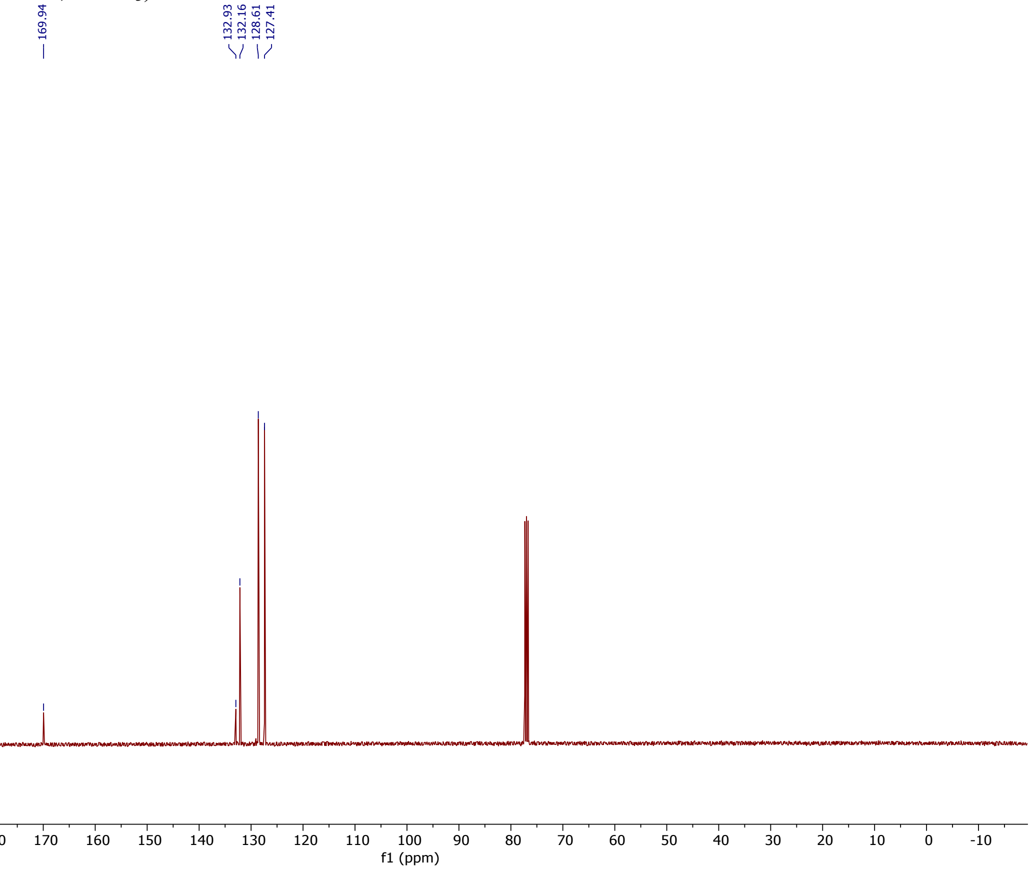

***N*-(4-bromobenzyl)-3-phenylpropanamide (4a):**

^{1}H NMR (400 MHz, $CDCl_3$)

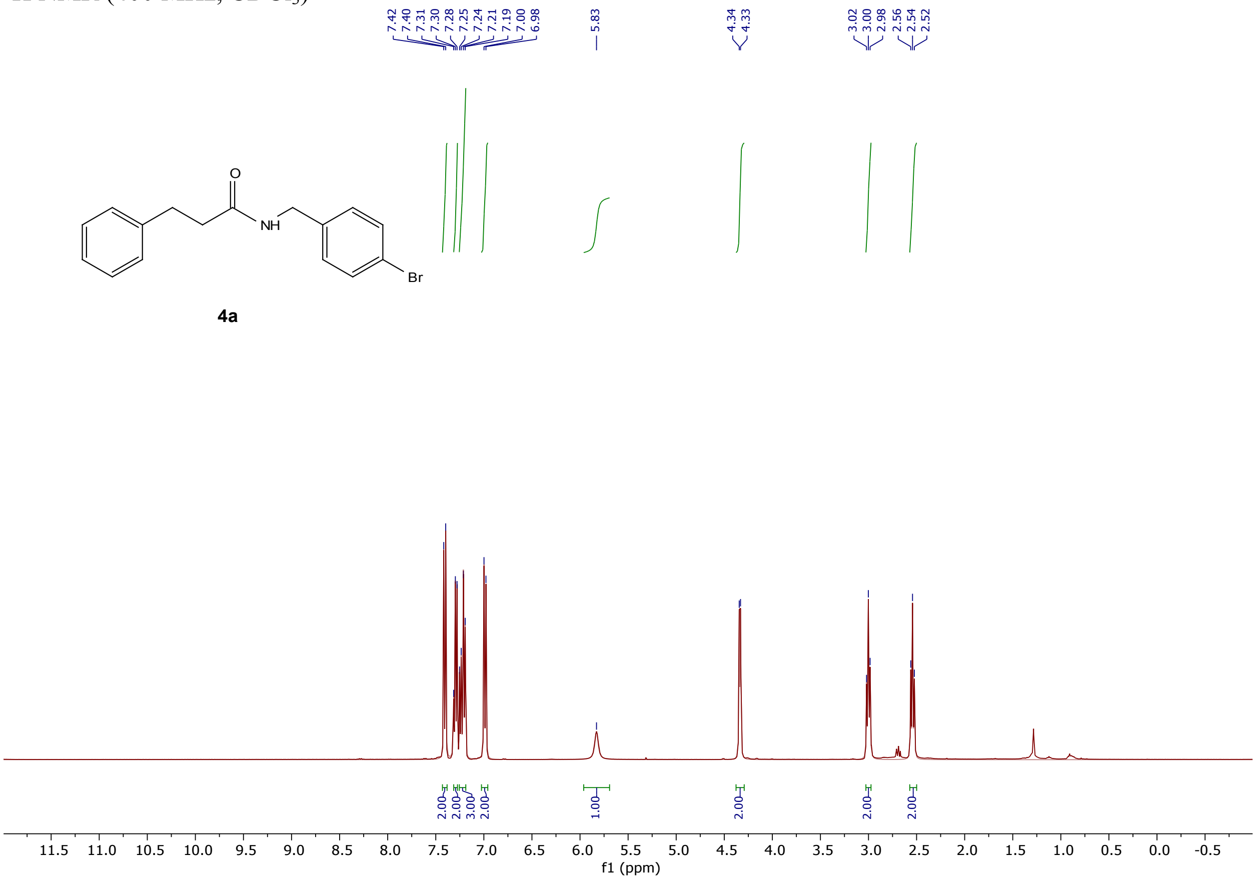

$^{13}C\{^{1}H\}$ NMR (100 MHz, $CDCl_3$)

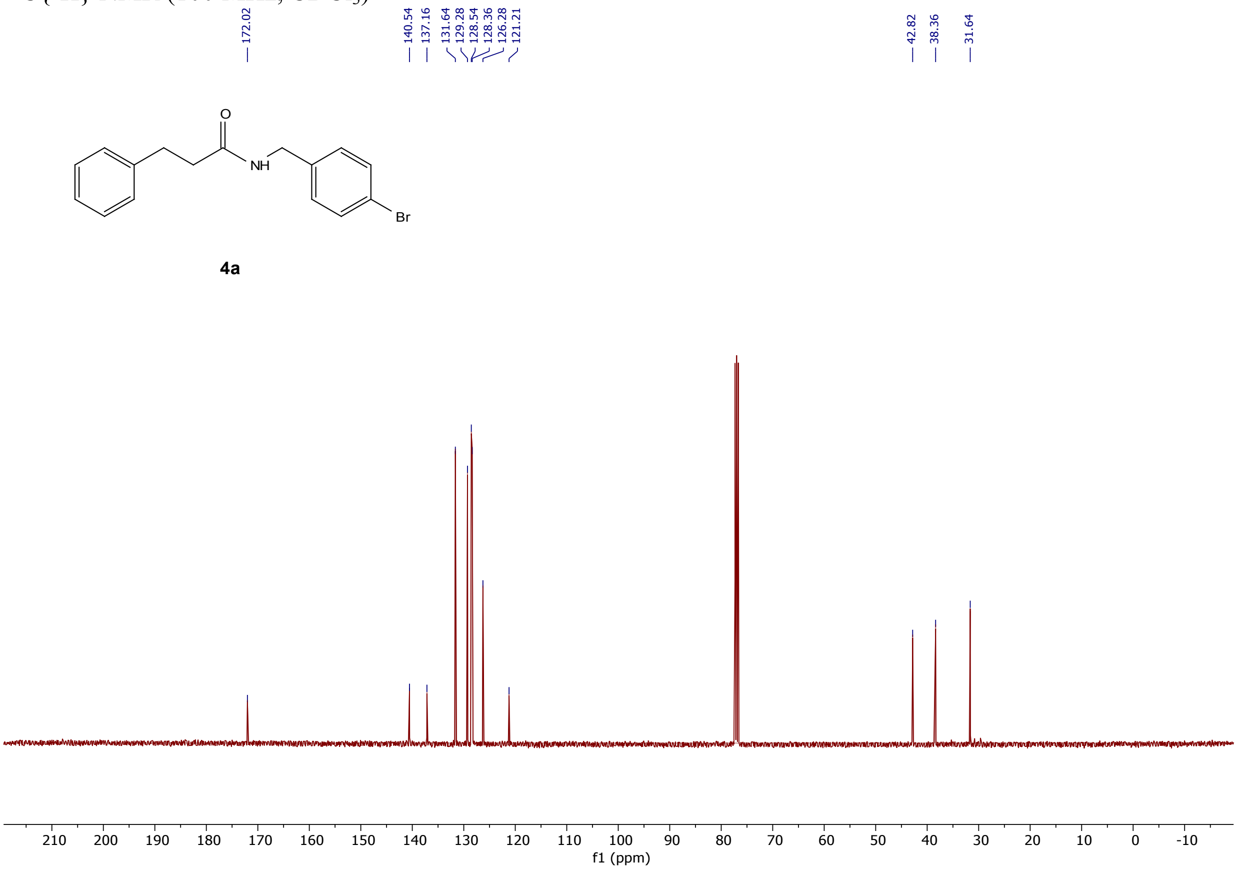

***N*-(2-bromobenzyl)isobutyramide (4b)**:

^{1}H NMR (400 MHz, $CDCl_3$)

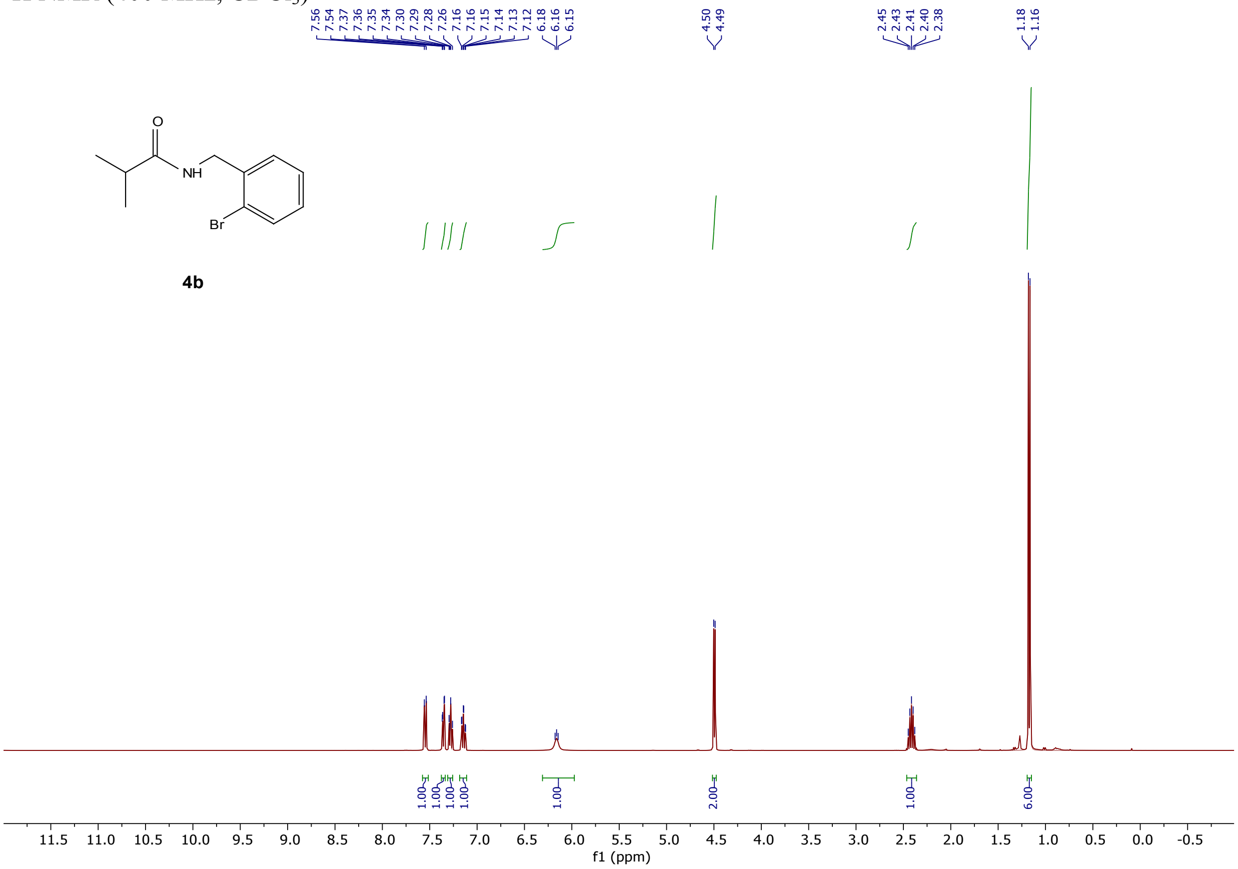

$^{13}C\{^{1}H\}$ NMR (100 MHz, $CDCl_3$)

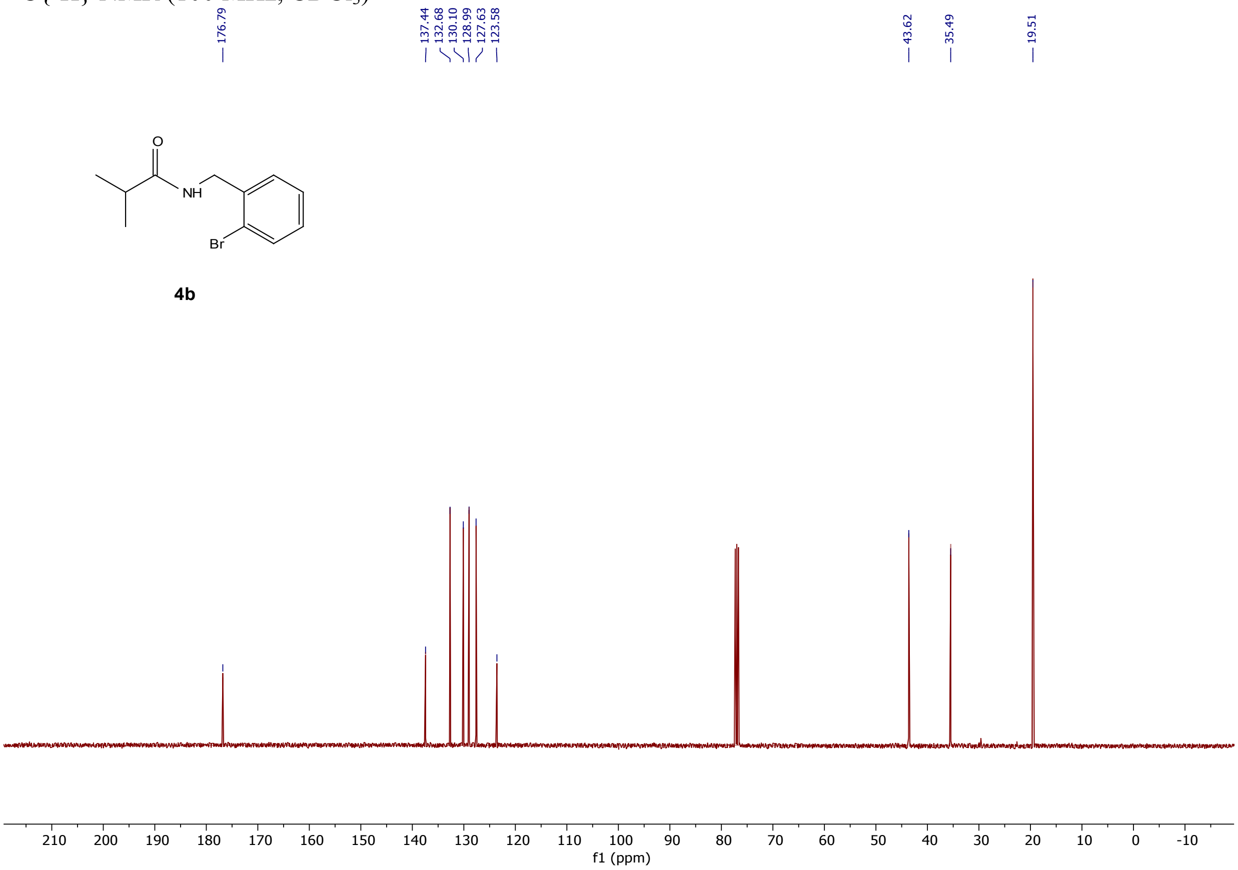

***N*-benzylcyclohexanecarboxamide (4c)**:

^{1}H NMR (400 MHz, $CDCl_3$)

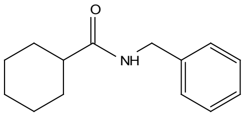

4c

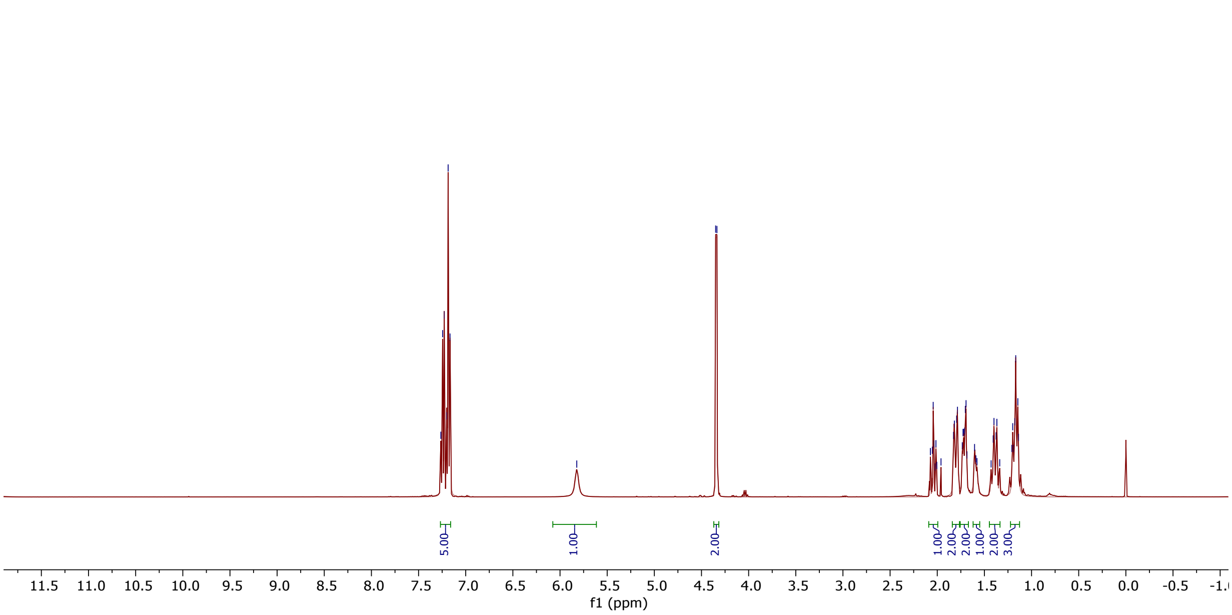

^{13}C{^{1}H} NMR (100 MHz, $CDCl_3$)

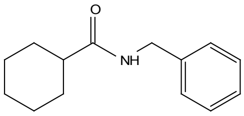

4c

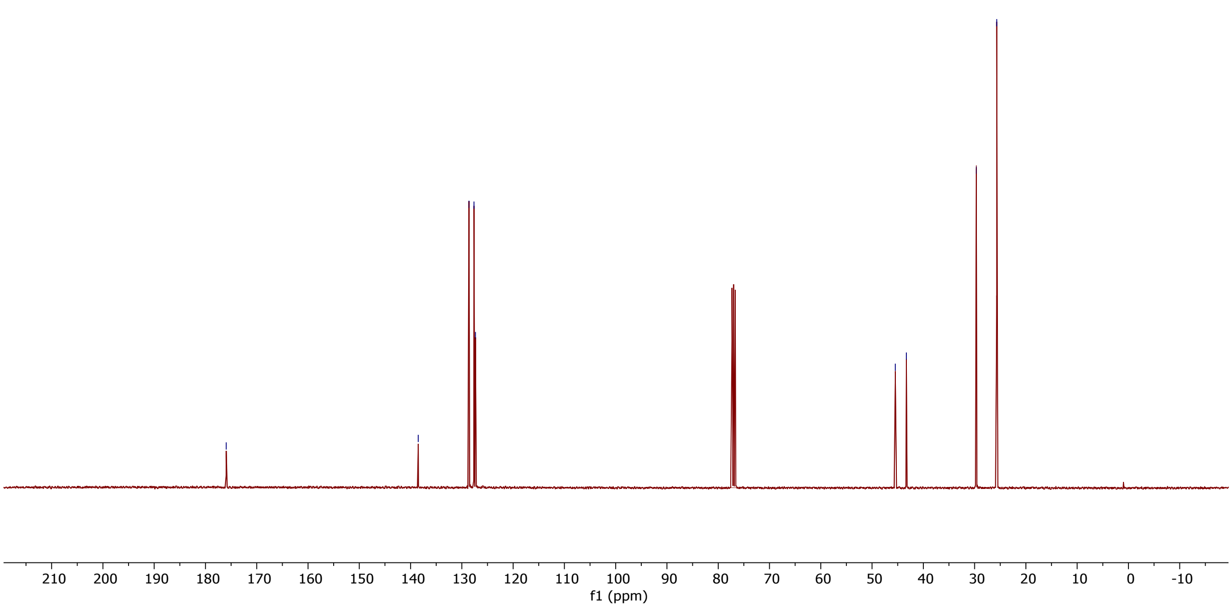

Supplement: Supplementary file 1 — jo0c01320_si_001.pdf [file jo0c01320_si_001.pdf]
